# The Effect of Chronic Methamphetamine Exposure on the Hippocampal and Olfactory Bulb Neuroproteomes of Rats

**DOI:** 10.1371/journal.pone.0151034

**Published:** 2016-04-15

**Authors:** Rui Zhu, Tianjiao Yang, Firas Kobeissy, Tarek H. Mouhieddine, Mohamad Raad, Amaly Nokkari, Mark S. Gold, Kevin K. Wang, Yehia Mechref

**Affiliations:** 1 Department of Chemistry and Biochemistry, Texas Tech University, Lubbock, TX, United States of America; 2 Department of Psychiatry, Center for Neuroproteomics and Biomarkers Research, University of Florida, Gainesville, FL, United States of America; 3 Faculty of Medicine, American University of Beirut Medical Center, Beirut, Lebanon; 4 Faculty of Medicine, Department of Biochemistry and Molecular Genetics, American University of Beirut Medical Center, Beirut, Lebanon; University of Nebraska Medical Center, UNITED STATES

## Abstract

Nowadays, drug abuse and addiction are serious public health problems in the USA. Methamphetamine (METH) is one of the most abused drugs and is known to cause brain damage after repeated exposure. In this paper, we conducted a neuroproteomic study to evaluate METH-induced brain protein dynamics, following a two-week chronic regimen of an escalating dose of METH exposure. Proteins were extracted from rat brain hippocampal and olfactory bulb tissues and subjected to liquid chromatography-mass spectrometry (LC-MS/MS) analysis. Both shotgun and targeted proteomic analysis were performed. Protein quantification was initially based on comparing the spectral counts between METH exposed animals and their control counterparts. Quantitative differences were further confirmed through multiple reaction monitoring (MRM) LC-MS/MS experiments. According to the quantitative results, the expression of 18 proteins (11 in the hippocampus and 7 in the olfactory bulb) underwent a significant alteration as a result of exposing rats to METH. 13 of these proteins were up-regulated after METH exposure while 5 were down-regulated. The altered proteins belonging to different structural and functional families were involved in processes such as cell death, inflammation, oxidation, and apoptosis.

## 1. Introduction

Methamphetamine (METH) has been recognized as one of the most abused drugs in the United States. METH is an illicit drug known to cause psychiatric manifestations such as euphoria, agitation, hallucinations, misperceptions, mood disturbances and long-term cognitive and psychomotor deficits [1–3]. These manifestations are mainly the result of neurotoxicity leading to striatal dopaminergic terminal degeneration [4, 5] and non-dopaminergic striatal pathologies [6]. Several studies have also shown that METH is a potent psychomotor stimulant that affects dopaminergic, glutamatergic and serotonergic systems in the brain [7, 8]. Findings similar to those observed in human brains have been reported in rodents treated with METH. METH has been implicated found to cause neurodegeneration [7, 9], oxidative damage [8], apoptosis [7, 10], and necrosis [11] throughout different brain regions as shown in S1 Fig. These observations may suggest that repeated METH exposure could induce adaptive changes in the brain with alterations in gene and protein expression, as well as structural modifications at dopaminergic, glutamatergic, and serotenergic synapses [12].

The mesocorticolimbic pathway, otherwise known as the reward pathway, is a dopaminergic circuitry connecting the ventral tegmental area, midbrain and nucleus accumbens implicated in structural and molecular changes in many forms of drug addictions, including METH [13]. Even though METH was found to leave long-lasting changes, such as decreased grey matter size, in many brain areas (caudate nucleus, prefrontal cortex, temporal cortex, anterior cingulate, amygdala, insula) [14–18]. Several studies have focused on the changes in the limbic system, specifically the hippocampus, owing to the fact that the main symptoms of METH abuse pertain to the limbic system [19–21]. Chronic METH exposure has been shown to lead to long-lasting cognitive deficits in clinical and in experimental models of METH abuse [22, 23]. In one study, it was found that the cognitive impairments via the hippocampal brain region did not occur during the drug exposure but rather as a later manifestation [24]. Upon using one chronic METH model in mice, it was found out that it was after a long period of drug abstinence that treated mice exhibited a deficit in spatial memory and hippocampal transmission [24]‎. This effect could be explained by cortical and striatal reductions in the dopamine transporter (DAT) and tyrosine hydroxylase [25–27]. This long-term or ‘delayed’ effect could be explained by the hypothesis that the monoamine terminal injury did reach a critical threshold or that during drug exposure compensatory mechanisms are possibly triggered, which mask clinical or physiological symptoms [24]. In support, the decreased striatal dopamine uptake and DAT ligand binding was seen in a high dose of METH administration were blocked when the same amount of METH was introduced over a longer period of time [28]. In addition, METH was even found to have a differential effect on different hippocampal sub-regions, such as inhibiting neurogenesis in the ventral spatial processing area, while blocking apoptosis in the dorsal behavior-regulatory area of the hippocampus [29].

Furthermore, Jayanthi et al. has shown that following two weeks of chronic METH exposure in rats (0.5 mg/kg/day and ending at 10 mg/kg/day), there was a down-regulation of striatal glutamate receptors, mediated *via* epigenetic mechanisms leading to a decreased expression of GluA1 mRNA and protein levels [30]. In another study by Yamamoto et al. it was indicated that mm mice exposed to chronic METH paradigm (a two week exposure of fixed 2mg/kg/day of METH dosage), they exhibited a differential expression of several genes related to glutamatergic neural transmission, including the NDMA receptor channel, in [31]. This genetic expression alteration might be part of the molecular basis of the behavioral sensitization to METH exposure. Furthermore, a two-week chronic and fixed METH exposure selectively increased nerve growth factors, Nur-related 1 and nerve growth factor inducible-B nuclear receptors, in different brain regions. The former is known to modulate development and differentiation of midbrain dopamine phenotypes, possibly through its rate limiting enzyme tyrosine hydroxylase. The latter is constitutively expressed in brain tissue and is a crucial regulator of signaling pathways of dopaminergic neurons and their targets [32]. However, other studies have also shown a dosage increment-dependent effect in a chronic two-week scenario. For example, it was found that a two-week exposure to METH in mice, a fixed METH regimen and not an escalated dose, may model the anxiety-related behavior observed in the dysphoric state during METH withdrawal in humans [33]. Hence, the action of METH on brain function seems to be dosage, interval and duration dependent.

From a clinical sense, a better understanding of the mechanism of METH addiction could provide better insight into the most appropriate way of managing and treating this drug problem. For instance, METH decreases D2/D3 dopamine receptors in addicts, that is in turn associated with a decreased gray matter volume of the misocorticolimbic circuitry (including the hippocampus) and which is negatively correlated with drug craving [34]. Thus, having a proteomic analysis of the genes involved in this consequence may aid in directing therapies for drug abusers and providing personalized prognostic values of successful METH abstinence.

Although very few studies have focused on the relation between the hippocampus and olfactory bulb, it has been shown that there are neuronal connections projecting from area CA1 of the ventral hippocampus to the olfactory bulb and from the latter to the entorhinal cortex, which bridges the hippocampus and the neocortex and has implications for memory formation [35]. One of the evidence of a strong relationship between the hippocampus and olfactory bulb is olfactory dysfunction in early Alzheimer’s disease, which primarily hits the hippocampal area[36]. Furthermore, abnormalities or size deficits in the olfactory bulb are associated with having major depressive disorder (MDD) [37, 38], a disorder that is both linked to the hippocampus [39] and drug addiction [40]. The intimate hippocampal-olfactory bulb connection was further appreciated in depression models of bulbectomized mice, whereby depressive symptoms were alleviated when administering vasoactive intestinal peptide (VIP), a neurotransmitter, into area CA1, which is directly connected to the bulb [41]. Therefore, with this set of data suggesting the anatomic and functional overlap between the hippocampal-olfactory bulb our neuroproteomic study is aimed at uncovering any relevant changes taking place in the region so closely associated with the hippocampus and associating its changes with METH exposure.

In the end, what remains unclear is how METH exposure leads to its clinical symptoms, the delay in their manifestation, and the differences that exist upon a prolonged METH exposure vs. acute high METH exposure. Hence, this study applies an advanced neuroproteomic approach to evaluating METH-induced brain protein dynamics, following a two-week chronic regimen of an escalating dose of chronic METH exposure. Proteins from of two brain regions including the hippocampus and the olfactory bulb were subjected to liquid chromatography-tandem mass spectrometry (LC-MS/MS) analysis. Both qualitative and quantitative data were obtained. Initially, quantification was based on comparing the spectral counts between METH-exposed animals and their control counterparts. Quantitative differences were further confirmed through multiple reaction monitoring (MRM) LC-MS/MS experiments. An advanced system biology approach was performed to extrapolate biological differences from the neuroproteomics data which highlighted a close relationship of the molecular pathways implicated in the hippocampal-olfactory bulb brain regions.

## 2. Methods

### 2.1. Animals

All animal care guidelines and proper approvals of animal committee at the University of Florida were acquired prior to conducting the research. All procedures involving animal handling and processing were done in compliance with guidelines set forth by the University of Florida Institutional Animal Care and Use Committee and the National Institutes of Health guidelines (IACUC). Animals were housed in groups of two per cage and maintained on a 12 h light/dark cycle (lights on 7 AM—7 PM). Food and water were available at libitum. All experiments were carried out on male Sprague Dawley rats which were divided into two groups: experimental drug group and a saline vehicle control group consisting of n = 5. The saline group received a similar injection of physiological saline. In this chronic model, all efforts were made to minimize the number of animals used and their suffering in accordance with the 2011 NIH Guide for the Care and Use of Laboratory Animals and the Guidelines for the Care and Use of Mammals in Neuroscience and Behavioral Research (National Research Council 2003). This chronic dose of METH paradigm shows no signs of distress or pain and is not associated with neurotoxicity. This dose of METH does not cause any neurotoxic effects as much acute doses (40 mg/kg) required to induce pathological changes. Animals were monitored continuously at 1 hour intervals during the drug treatment, i.e. every 1 hr from 7 am to 7 pm. Our lab used a digital thermometer to measure rectal temperature and assess any temperature alteration before each METH injection and 1 h after each successive injection. If the rat body temperature reached 40°C, rats were cooled by moving them in a cage with ice.

### 2.2. Chemicals

Pharmacologic agent (+/-) methamphetamine hydrochloride, dithiothreitol (DTT), iodoacetamide (IAA), ammonium bicarbonate and MS-grade formic acid were purchased from Sigma-Aldrich (St. Louis, MO). Tris was obtained from Shelton Scientific, Inc (Peosta, IA). Urea was purchased from Thermo Scienfic (Rockford, IL). HPLC grade water was acquired from Mallinckrodt Chemicals (Phillipsburg, NJ). HPLC grade acetonitrile was acquired from J.T.Baker (Phillipsburg, NJ). Trypsin Gold, Mass Spectrometry Grade was obtained from Promega (Madison, WI).

### 2.3. Collection of rat brain tissues

Rat brain tissues were collected using a published method [42]. Briefly, the model of chronic METH abuse was designed using male Sprague-Dawley rats infused with METH over a period of 14 days. The concentration and frequency of drug administration were altered to fit a chronic escalating dose of METH exposure. All experiments were performed using male Sprague-Dawley rats that were aged 60 days and weighed between 250 and 275g. Pharmacologic agent (+/-) methamphetamine hydrochloride was dissolved in 0.9% saline. Five rats were intraperitoneally injected (ip) with 0.5 mg/kg of METH on day 1 and gradually increased the dose by 0.5mg/day to end with 8 mg/kg at the end of the second week. Also, 5 rats received physiological saline injections. Two weeks post-ip injection, treated and control animals were briefly anaesthetized with 3–4% isoflurane and sacrificed by decapitation. METH and saline hippocampal samples were rapidly dissected and washed with saline solution, snap-frozen in liquid nitrogen, and stored at -80°C for further processing.

### 2.4. Extraction and tryptic digestion of proteins

Frozen rat brain hippocampal and olfactory bulb tissues were homogenized using VWR® Disposable Pellet Mixers (VWR International, Radnor, PA) in 500-μL extraction buffer (5M urea, 40mM Tris, 0.2%w/v CHAPS). Next, the sample was sonicated for 1 hour at 4°C prior to centrifugation for 45 min at 14,800 rpm and temperature of 4°C. The supernatant was then collected in separate containers. The buffer of the extracted protein was exchanged into 50 mM ammonium bicarbonate using 5kDa MWCO spin concentrators (Agilent Technologies, Santa Clara, CA). This buffer is needed for efficient tryptic digestion.

A 10-μg aliquot of each sample, determined by BCA protein assay (Thermo Scientific/Pierce, Rockford, IL), was diluted to 20-μL by 50 mM ammonium bicarbonate. Thermal denaturation was performed at 65°C for 10 min. A 0.75-μL aliquot of 200mM DTT was added to reduce the sample at 60°C for 45 min. A 3-μL aliquot of 200mM IAA was added to alkylate the sample at 37.5°C for 45 min in the dark. Excess IAA was consumed by the addition of another 0.75-μL aliquot of 200mM DTT and incubation at 37.5°C for 30 min. The tryptic digestion was performed at 37.5°C for 18 hours followed by microwave digestion at 45°C and 50W for 30min. A 0.5-μL aliquot of formic acid was added to quench the digestion. Finally, a 3-μL aliquot of 5 ng/μL reduced and permethylated dextran was added to each sample as an internal standard to offset any potential injection variance.

### 2.5. LC-MS/MS analysis

LC-MS/MS was acquired using Dionex 3000 Ultimate nano-LC system (Dionex, Sunnyvale, CA) interfaced to LTQ Orbitrap Velos and TSQ Vantage mass spectrometers (Thermo Scientific, San Jose, CA) equipped with nano-ESI source.

The separation was attained using Acclaim PepMap RSLC columns (75 μm I.D. x 15 cm, 2 μm particle sizes, 100 Å pore sizes) (Dionex, Sunnyvale, CA) with a flow rate of 350 nL/min. The column compartment was maintained at 29.5 °C. The LC elution gradient of solvent B used in both LC-MS/MS analysis was: 5% over 10 min, 5%-20% over 55 min, 20–30% over 25 min, 30–50% over 20 min, 50%-80% over 1 min, 80% over 4 min, 80%-5% over 1 min and 5% over 4 min. Solvent B consisted of 100% ACN with 0.1% formic acid while solvent A was composed of 2% ACN and 0.1% formic acid.

The LTQ Orbitrap Velos mass spectrometer was operated in positive mode with the ESI voltage set to 1500V. Data dependent acquisition mode was employed to achieve two scan events. The first scan event was a full MS scan of 380–2000 *m/z* at a mass resolution of 15,000. The second scan event was CID MS/MS of parent ions selected from the first scan event with an isolation width of 3.0 *m/z*, at a normalized collision energy (CE) of 35%, and an activation Q value of 0.250. The CID MS/MS scans were performed on the 30 most intense ions observed in the MS scan event. The dynamic exclusion was set to have repeat count of 2, repeat duration of 30 s, exclusion list size of 200 and an exclusion duration of 90 s.

The TSQ Vantage mass spectrometer was operated in positive mode with an ESI voltage of 1800V. Data independent acquisition mode was used for MRM experiment. Predefined precursor and transition ions were monitored to select specifically targeted peptides corresponding to each candidate protein with 10.0 sec chromatogram filter peak width. The MRM experiments were performed at a cycle time of 2.000 sec and a Q1 peak width of 0.70 min for 400–1500 *m/z* mass range. The normalized collision energy value was 30% with a collision gas pressure of 1.5 mTorr in Q2.

### 2.6. Data analysis

LC-ESI-MS/MS data was used to generate mascot generic format file (*.mgf) by Proteome Discover version 1.2 software (Thermo Scientific, San Jose, CA) then searched using SwissProt database (Rattus) in MASCOT version 2.4 (Matrix Science Inc., Boston, MA). Iodoacetamide modification of cysteine was set as a fixed modification while oxidation of methionine was set as a variable modification. An m/z tolerance of 5 ppm was set for the identification of peptides with maximum 2 missed cleavages. Also, tandem MS ion tolerance was set within 0.8 Da with label-free quantification. Scaffold Q+ (Proteome Software, Portland, OR) was employed for spectral counts quantitation. Proteins are shown significant difference (p<0.05, unpaired student t-test) in spectral counts quantification results were confirmed by MRM LC-MS/MS experiment. Each sample was injected three times to make a technical triplicate of MRM experiment. The most intense 1 or 2 peptides, corresponding to each candidate protein, were selected as target peptides. The three transitions of each target peptide were suggested by Pinpoint (Thermo Scientific, San Jose, CA). The MRM experiment results were investigated using Pinpoint. The peak area of each target peptide was normalized by the peak area of the glycan with 4 glucose units (*m/z* = 896.507). The normalized intensity of target peptides corresponding to each candidate protein was summed up to represent the abundance of that specific protein. A one-way ANOVA test (α = 0.05) was performed to evaluate statistical significance. Finally, a systems biology analysis was carried out on the proteins exhibiting significant up- or down-regulations, using PANTHER (Protein Analysis Through Evolutionary Relationships) system and Pathway Studio 8.

## 3. Results

### 3.1. Protein Extraction Efficiency

**Table 1** shows the extraction efficiency of rat brain tissues. In general, the average protein extraction efficiencies are 7.27 ± 0.32%, 7.51 ± 0.32%, 8.31 ± 0.44%, 7.98% ± 0.23% for METH-treated hippocampal tissues, saline-injected hippocampal tissues, METH-treated olfactory bulb tissues, and saline-injected olfactory bulb tissues, representatively. These results show a reproducible protein extraction for a different type of brain tissues.

**Table 1:** Extraction efficiency of rat brain tissues.

Sample	Mass (mg)	Extracted Protein Mass (mg)	Extraction Efficiency
HM2	112.00	7.20	6.43%
HM3	100.03	6.91	6.90%
HM4	99.68	7.34	7.36%
HM5	109.53	7.98	7.28%
HM6	97.96	8.19	8.36%
HS3	107.96	7.50	6.95%
HS4	104.73	7.68	7.34%
HS5	98.48	7.20	7.31%
HS6	110.10	7.92	7.20%
HS7	81.33	7.11	8.75%
OM4	76.66	5.74	7.49%
OM5	78.38	6.03	7.69%
OM6	81.83	6.48	7.92%
OM7	53.43	5.30	9.92%
OM8	76.41	6.52	8.53%
OS2	63.90	5.22	8.17%
OS3	88.76	6.97	7.85%
OS4	89.12	6.39	7.17%
OS5	87.27	7.12	8.15%
OS6	92.11	7.87	8.55%

HM: METH-treated hippocampal tissue, HS: Saline-injected hippocampal tissue, OM: METH-treated olfactory bulb tissue, OS: Saline-injected olfactory bulb tissue. The extracted protein mass was determined by a BCA protein assay, and extraction efficiency was calculated using the expression. Extraction Efficiency = (Extracted protein/mass Sample mass) x 100.

### 3.2. Shotgun Proteomics

LC-MS/MS (2hrs) analyses of the hippocampal proteome extracted from rats permitted the total identification of 1829 unique peptides corresponding to 302 proteins at 0.1% FDR. For the METH-treated group (N = 5), a total of 1628 unique peptides corresponding to 301 proteins were identified. One sample of the control group was not sucessfully analyzed due to the injection issue, thus, only 1481 unique peptides corresponding to 298 proteins were identified for the control group (N = 4). A total of 297 proteins and 1260 unique peptides were identified in both groups. There were 4 proteins unique to the METH-treated group while there was only one protein unique to the control group.

On the other hand, LC-MS/MS (2hrs) analyses of the olfactory bulb proteome extracted from rats permitted the identification of 1975 unique peptides corresponding to 336 proteins at 0.1% FDR. For the METH-treated group (N = 5), a total of 1750 unique peptides corresponding to 335 proteins were identified, while 1640 unique peptides corresponding to 333 proteins were identified in the control group (N = 5). Consequently, 332 proteins and 1415 peptides were identified in both groups. There were 3 proteins unique to the METH-treated group while only one protein unique to the control group.

### 3.3. Spectral Count Quantification

Spectral counts of identified proteins were used to perform a preliminary label free quantification. Among the total 302 protein identified in the hippocampal tissues, 42 proteins were found to be significantly different based on an unpaired student t-test (p< 0.05). According to the spectral count data, 31 proteins were up-regulated while 11 proteins were down-regulated following METH treatment. Similarly, for the 335 protein identified in the olfactory bulb tissues, 25 proteins were found to be significantly different based on an independent t-test (p< 0.05). Fourteen proteins were up-regulated while 11 proteins were down-regulated after METH treatment. The entire list of identified proteins of the hippocampal and olfactory bulb proteome, quantitative spectra counts value and numbers of unique peptides corresponding to each identified proteins are listed in **S1–S4 Tables**.

**Fig 1:**
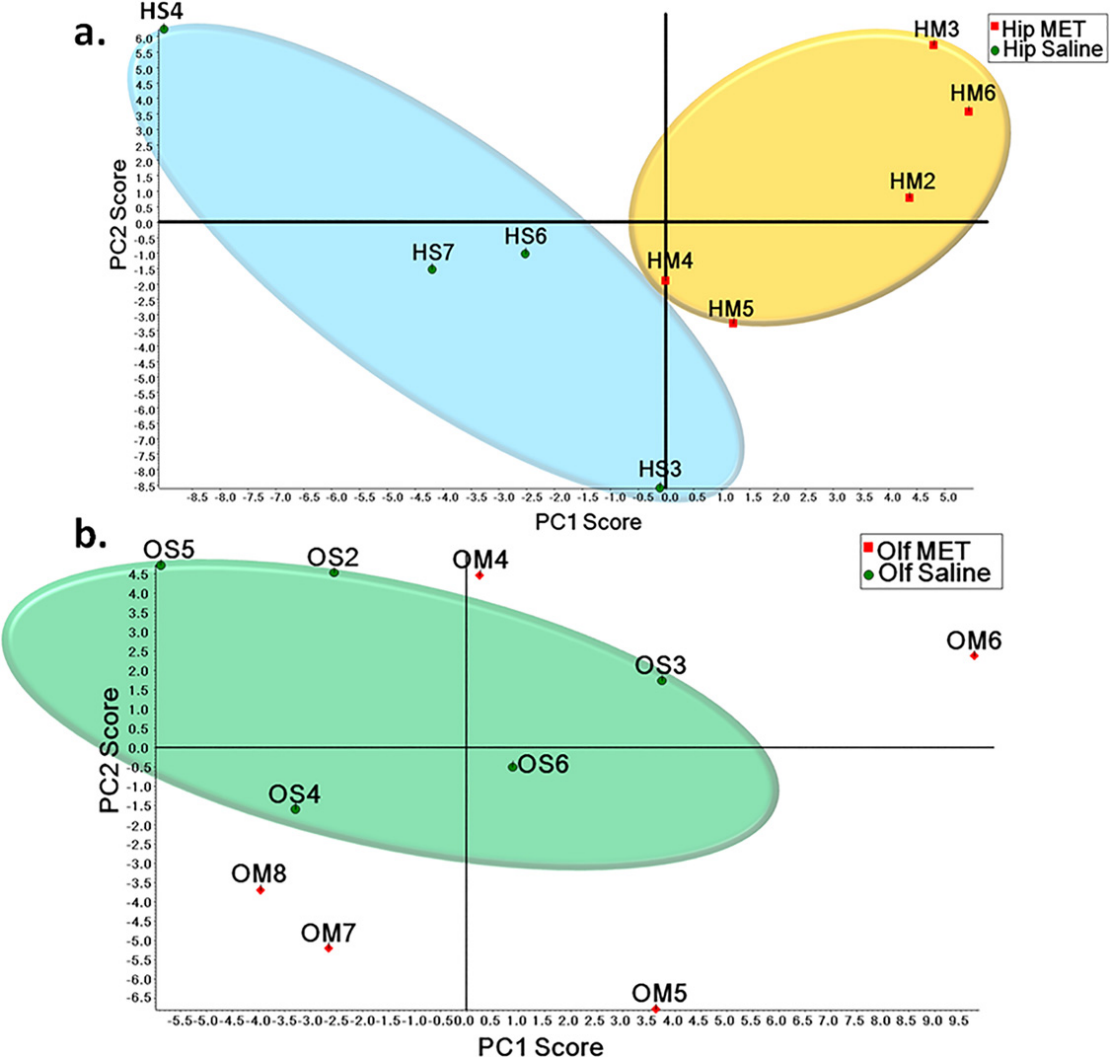
Principal component analysis (PCA) of spectral count data. (a) PCA of spectral count data of hippocampal proteome. (b) PCA of spectral count data of olfactory bulb proteome.

Also, a principal component analysis (PCA) was performed on the spectral count data to investigate internal variation between the METH-treated group and control group. **Fig 1** depicts the PCA scoring plots of the hippocampal and olfactory bulb proteome. According to PCA, the experimental and control groups of the hippocampal proteome were clearly non-overlapping. For the PCA plot of the olfactory bulb proteome, we were able to cluster together the control group, while those of the experimental group were scattered around the control group. This might suggest that METH treatment has more influence on the hippocampal rather than olfactory bulb proteomes.

### 3.4. Targeted Proteomics

In order to further confirm the quantification of proteins, all proteins that showed a significant difference in expression between the experimental and control groups, were subjected to MRM experiments. A list of target proteins and transitions are shown in **S5 Table** and **S6 Table**. The MRM quantification was based on the normalized peak area of transitions. A one-way ANOVA test was used to evaluate the statistical significance between the experimental and control groups. For hippocampal proteins, 79 peptides corresponding to the aforementioned 42 proteins were subjected to the MRM experiment. 37 proteins had 2 peptides corresponding to each of them while 5 proteins had 1. The MRM experiment detected 73 peptides corresponding to 41 proteins, 33 of them had 2 peptides corresponding to each one while 9 of them had 1. According to the MRM quantification, 11 proteins were shown to be significantly different between the experimental and control group, 7 of them were up-regulated while 4 were down-regulated. For the olfactory bulb proteins, 46 peptides corresponding to the aforementioned 25 proteins were subjected to the MRM experiment. Twenty-one proteins had 2 corresponding peptides while 4 proteins had 1. In this case, the MRM experiment detected 34 peptides corresponding to 22 proteins, 12 of them had 2 peptides while 10 proteins had 1. According to the MRM quantification, 7 proteins were shown to be significantly different between the experimental and control group, 6 of which were up-regulated while 1 was down-regulated.

**Fig 2:**
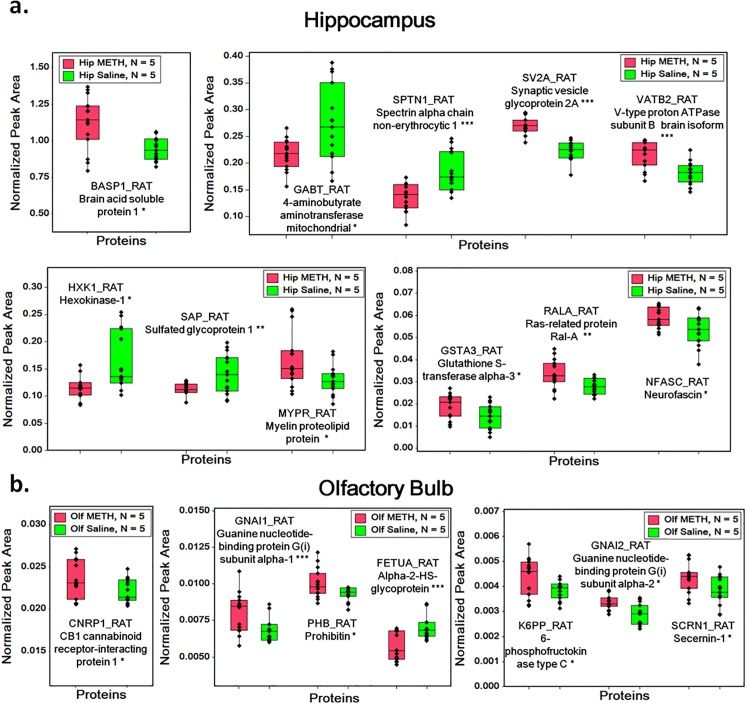
Box graphs of MRM quantification results of proteins shown significant differences. (a) Box graph of MRM quantification results of hippocampal proteins. (b) Box graph of MRM quantification results of olfactory bulb proteins. *: 0.005<p<0.05, **: 0.001<p<0.005, ***: p<0.001.

The box graphs shown in **Fig 2** summarize the MRM quantification results of proteins that exhibited significant changes as a result of METH exposure. Among the affected hippocampal proteins, synaptic vesicle glycoprotein 2A exhibited the most significant up-regulation (p = 0.0001 and +18%) while spectrin alpha chain non-erythrocytic 1 exhibited the most significant down-regulation (p = 0.0002 and -26%). As for the olfactory bulb, guanine nucleotide-binding protein G (i) subunit alpha-1 exhibited the most significant up-regulation (p = 0.001 and +18%) while Alpha-2-HS-glycoprotein exhibited the most significant down-regulation (p = 0.0005 and -20%).

### 3.5. Systems Biology Assessment

**Fig 3:**
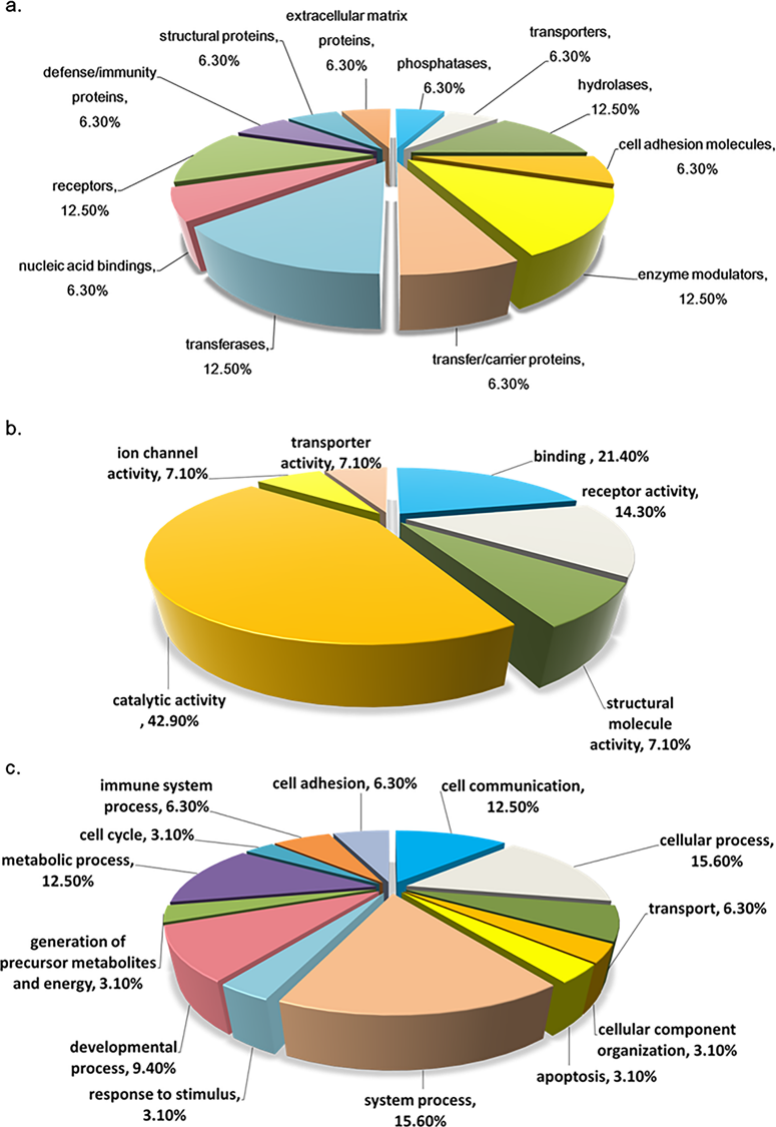
Gene ontology of METH-induced altered proteins. (a) Genes profile protein class of METH-induced altered proteins. (b) Genes profile molecular function of METH-induced altered proteins. (c) Genes profile biological process of METH-induced altered proteins.

For a more comprehensive understanding of the classes of differentially expressed proteins found in both brain regions following METH treatment, PANTHER analysis (http://www.pantherdb.org/genes/batchIdSearch.jsp) was used to classify proteins, via a rat protein ontology database, into distinct categories of molecular function, biological process, and protein class. The gene ontology of METH–induced altered proteomes in both brain regions is depicted in **Fig 3**. According to **Fig 3A**, the most common METH-altered molecular function is catalytic activity; 42.90% of the proteins were found to have this function. According to **Fig 3B,** cell communication, and system process are the two biological processes most involved in the METH–induced altered proteins, whereby each one of these processes constitutes 15.9% of the METH–induced altered proteins. According to **Fig 3C**, the METH–induced altered proteins can be evenly classified into different protein classes, such as receptor (12.50%), hydrolase (12.50%), enzyme modulator (12.50%) and transferase (12.50%).

**Fig 4:**
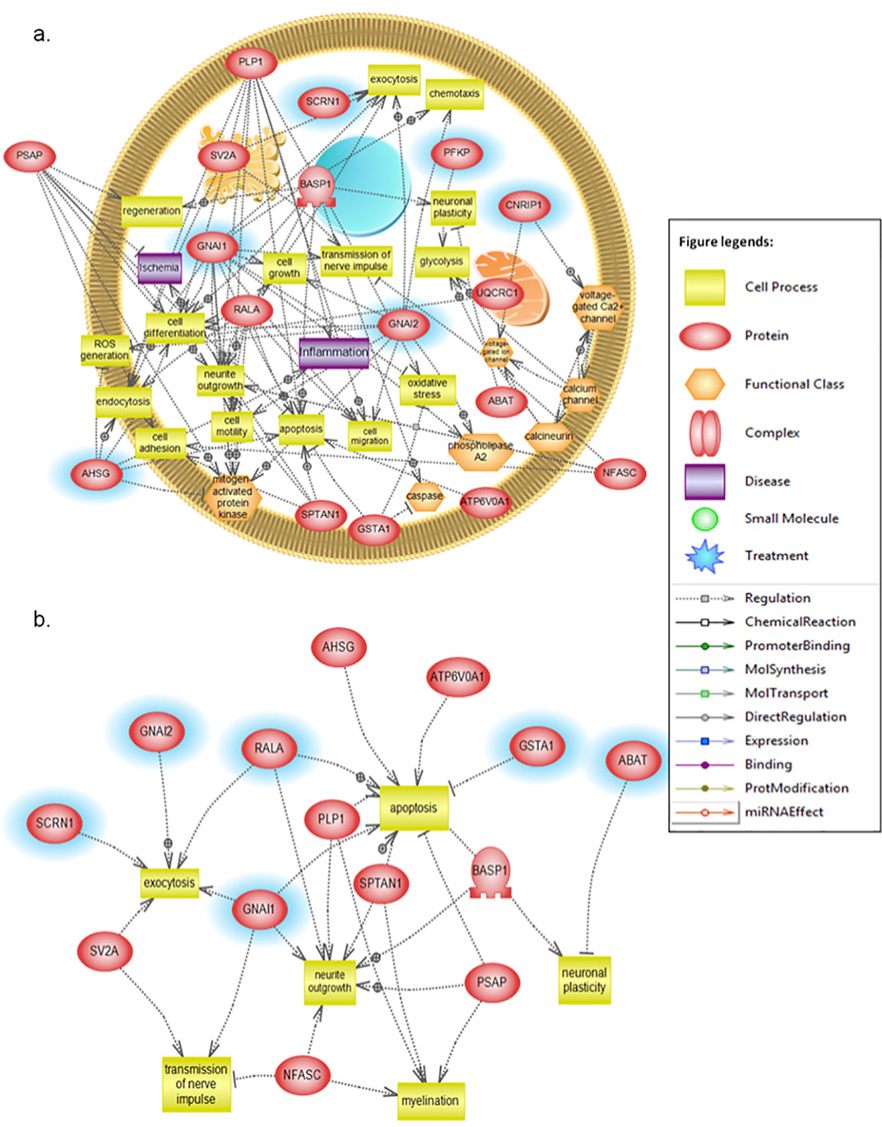
Pathway study of METH-induced altered proteins. The shape of a given protein is indicative of its functional class as shown in the legend. The definition of the lines connecting two proteins is included in the legend. (a) Global interaction proteome. (b) Enriched pathways and networks.

The Pathway Studio 8 (2011) software was used to search for possible protein-protein interactions, common regulators, cell processes, and related pathways for associations with METH–induced altered proteins. The network was generated using Shortest Path algorithm to map interactions between the affected proteins. A simplified picture of their interactions is shown in **Fig 4**. Through this approach, we found that proteins belonging to different structural and functional families were involved in processes such as cell death, inflammation, oxidation, and apoptosis.

## 4. Discussions

Profiling of the proteome of rat hippocampus and olfactory bulb was attained through shotgun proteomics using LC-ESI-MS/MS. Protein quantification was first performed employing label-free spectral count method and was further confirmed by MRM experiment. This quantization strategy successfully revealed a significant alteration in the expression of 18 proteins (11 in the hippocampal proteome and 7 in the olfactory bulb proteome) as a result of exposure of rats to METH. Thirteen of these proteins were up-regulated after METH exposure while 5 were down-regulated. It has been hypothesized that METH induced dopamine redistribution to produce reactive oxygen species (ROS) that may be responsible for its neurotoxicity, and thus, resulting in clinical psychosis [43, 44]. Following two weeks of exposure to METH in rats, Jayanthi et al. demonstrated a downregulation of striatal glutamate receptors via diverse epigenetic mechanisms, mainly histone hypoacetylation [30]. In another study, Yamamoto et al. also demonstrated a possible molecular basis of the behavioral sensitization to METH through an alteration in several genes related to glutamatergic neural transmission [31]. In addition, METH has been shown to increase ROS production by up-regulating pro-apoptotic genes such c-*Jun*, c-*myc* and L-*myc* [45–47]. In support, METH also induced apoptosis in a CNS-derived catecholaminergic cell line [48] and an immortalized rat striatal cell line [49]. Hence, we expect and as our results show through the above-mentioned protein expression, up- and down-regulation of many biological pathways involved in cell death, neuronal repair, inflammation, oxidation, and apoptosis. The results of this study enabled a better understanding of the effect of METH exposure on rat brain proteome. The findings of this study may help in devising a plan to overcome substance abuse.

### 4.1. Hippocampal Tissue

In a previous study, METH was shown to cause dopaminergic and some serotonergic nerve terminal degeneration in the hippocampus [50]. The down-regulation of 4-aminobutyrate aminotransferase and spectrin, alongside the up-regulation of BASP1, point towards an increased cell death rate. Glutamate and the increase of intracellular calcium have been implicated in neuro-toxic cell death [51–53]. Furthermore, accelerating the metabolism of glutamate into succinic acid via GABA shunt enzymes, including 4-aminobutyrate aminotransferase, was neuroprotective against ischemia and reperfusion injury [54]. Hence, METH-induced down-regulation of 4-aminobutyrate aminotransferase (p = 0.013) may indicate a possible pathway of neurotoxicity and neuronal cell death. BASP1 (up-regulated; p = 0.01) controls several processes by binding calcium calmodulin and affecting its interaction with downstream targets [55, 56]. It is interesting to note that an overproduction of BASP1 in one study resulted in apoptosis of neuronal cells [57], possibly through an enhanced extra-cellular calcium influx that triggers an apoptotic cascade within the cell. Thus, we hypothesize that a METH-induced decrease in 4-aminobutyrate aminotransferase could be leading to un-opposed hippocampal tissue death via calcium influx, which is activating a cellular defense mechanism by up-regulating BASP1 that binds calmodulin, preventing the activation of downstream apoptotic signaling via the increased intracellular calcium levels. Spectrin (down-regulated; p = 0.0002) has been found to be a marker of traumatic brain injury and neurodegeneration in numerous studies [58–60]. Its proteolysis is thought to involve calpain [61], and may be physiologically involved in synaptic remodeling, long-term potentiation, and memory formation [62]. In another study, METH was shown to produce a significant increase in calpain- and caspase-cleaved alpha II-spectrin, suggesting cell injury or death [61, 63]. These findings are in accordance with a high calcium influx that is activating cellular calpains, which in turn are degrading the spectrin protein [63]. It appears that calcium plays a major role in the observed molecular and clinical manifestations following METH exposure. Therefore, calcium seems to be a potential therapeutic target to decrease the long-lasting detrimental effects of METH on the hippocampus, and other brain regions, of METH addicts. Yet, further studies focusing on the mechanistic role of calcium in neuronal injury, after drug exposure, are warranted before concrete conclusions could be established.

In opposition to the above-mentioned neuronal injury pathways, increased GSTP1 and Ral and the down-regulation of hexokinase, point towards neurodegenerative processes. Many GST isoenzymes have been implicated in cell signaling and cell cycle control and apoptosis [64–66]. Increased GSTP levels have been correlated with decreased apoptosis, possibly by suppressing the JNK pathway-dependent apoptosis [67]. GSTP1 has also been shown to inhibit directly cyclin dependent kinase 5 and ROS that eventually result in apoptosis. In another study, GSTP1 was shown to be neuroprotective under neurotoxic conditions [68] implying that GSTP1 levels may modulate Cdk5 signaling, eliminate oxidative stress, and prevent neurodegeneration, as in the case of our study. Similarly, inhibition of hexokinase (down-regulated; p = 0.013) has been shown to lead to neuroprotection in hippocampal slice culture [69]. Ral (up-regulated; p = 0.0036) a neuronal protein involved in controlling postsynaptic plasticity has been shown to take part of neuro-regeneration and dendritic growth [70]. As a conclusion, the change in expression level of the above mentioned proteins could be due to a counteractive reaction towards the neuronal insult of METH as the neuronal cells of the hippocampal region are activating anti-apoptotic pathways to induce regeneration of the disrupted circuitry, leading to a high turnover of hippocampal tissue. This turnover could also be deduced from the fact that we found the myelin proteolipid protein (PLP) to be up-regulated. PLP is known to affect the stabilization of the myelin sheath and was shown to be down-regulated in rat brains upon prolonged ethanol administration [71].

Neurofascin is a cell adhesion molecule involved in axonal guidance, synaptogenesis, and neuronal interactions and is known to trigger presynaptic stabilization via trans-synaptic adhesion to presynaptic ligands [72]. Moreover, neurofascin is well known to contribute to the localization of inhibitory synapse through the induction of gephyrin clusters. It is unclear how an up-regulation of neurofascin would act in the pathology of METH on the hippocampus, but it could possibly be up-regulated, as in the case of Ral, to guide the newly produced outgrowths and neuronal interactions needed to adopt proper connections. It is also unclear how high SV2A contributes to the pathophysiology seen in METH treatments but since low SV2A has been shown to contribute to epileptogenesis and seizure propagation [73], it seems that up-regulating SV2A could be an anti-epileptic defense mechanism against the disrupted neuronal connections that can serve as epileptogenic foci.

### 4.2. Olfactory Bulb

The olfactory bulb is a brain region that takes part in a psychological performance in both the normal and pathological states [74, 75] via the limbic system [76]. For example, the olfactory function is imperative in animal courtship, mating, and reproduction [77, 78]. A reduced olfactory bulb size is observed in schizophrenic patients [79] and associated with hyposomnia in patients who suffer from Parkinson’s disease [80, 81]. Interestingly, olfactory bulbectomy has often been used as an animal model of depression [74]. Rodents undergoing bulbectomy have been used to model psychomotor depression and aggressive behavior such as in-cage aggression towards other animals [82, 83].

Interestingly, these behaviors are in parallel with the bite attacks reported in mice chronically treated with METH [84]. Thus, it might be worthwhile to evaluate whether METH actually causes neuronal loss or damage in the olfactory bulb to mimic what was observed in the bulbectomized mice. Only two studies looked into the effect of METH on neuronal cells in the olfactory bulb. The study demonstrated that METH led to a loss of olfactory bulb dopaminergic terminals and death of dopaminergic neurons [85]. In another study, METH was shown to lead to postnatal neurodevelopmental deficits in activity and olfactory function [86]. Put together, the significantly altered olfactory bulb proteins in our results point to altered cell division properties, neuroprotection, and a paradoxical increase and decrease in oxidative stress pathways. This suggests that METH might induce neuronal cell death as well as corrective repair mechanisms [87]. 6-phosphofructokinase type C (up-regulated; p = 0.012) is an enzyme involved in the glycolytic pathway. When up-regulated, the enzyme has been shown to increase the glycolytic pathway and decrease the pentose phosphate pathway, increasing oxidative stress, and apoptotic neuronal death [88, 89]. Alpha-2-HS-glycoprotein, also known as fetuin-A (down-regulated; p < 0.0005), is a neuroprotective protein the function of which has been demonstrated in a rat stroke model [90] and in early brain ischemic injury, whereby it attenuates the brain inflammatory response [91]. It has also been shown to play a part in brain development [90]. Hence, the up-regulation of 6-phosphofructokinase type C and down-regulation of fetuin-A suggest an increased oxidative stress in the olfactory bulb, which may explain the increased neuronal cell death, as previously demonstrated by Deng et al. [85].

Contrary to the above results, and as seen in the hippocampal tissue, prohibitin, an essential mitochondrial protein implicated in various cellular functions, was up-regulated (p = 0.042). Even though its function is suggestive of oxidative stress protection, its exact role, and mechanism in neuroprotection is still to be validated. Its neuroprotective function was elucidated when it was found to be up-regulated in an *in vivo* model of transient ischemia and oxygen-glucose deprivation in neuronal cultures [92, 93]. When silenced, prohibitin was shown to increase neuronal vulnerability, which may be associated with the loss of mitochondrial membrane potential and increased ROS. In another study, up-regulation of prohibitin was related to reduced ROS [94]. Hence, the significant up-regulation of prohibitin in our study suggests the induction of reparative processes through a decreased oxidative stress.

Up-regulated Guanine nucleotide-binding protein G(i) subunit alpha-1, Secernin-1, Guanine nucleotide-binding protein G(i) subunit alpha-2 have all been associated with cell division and cancer [95–98]. However, it is still unclear whether this effect on cellular signaling and cell division is in favor of protection or death of the olfactory bulb neurons upon METH administration.

Therefore, we found that METH induced both neuroprotective and neurodetrimental signaling pathways within the neurons of the hippocampus and olfactory bulb, but which were conducted via different proteins. This differential proteome alteration could be attributed to the structural and functional differences between the two regions and knowledge of the altered proteins can thus aid us in administering brain region-specific therapies for METH addicts.

### 5. Concluding Remarks

In this work, shotgun proteomics allowed us to profile successfully the proteome of the rat hippocampus and olfactory bulb. As a result of chronic METH exposure, protein alteration was efficiently interpreted by target proteomics using MRM experiments. In total, 18 proteins (11 in the hippocampal and 7 in olfactory bulb proteome) were significantly altered as a result of METH exposure on rats and among those altered proteins, 13 were up-regulated while 5 were down-regulated. The altered proteins are related to many biological pathways such as cell death, neuronal repair, inflammation, oxidation, and apoptosis. The findings of this study enabled a better understanding of the biological function of these altered proteins and how they affect rat brain proteome after METH exposure (S1 Fig). Furthermore, this study will help in studying and overcoming METH abuse in humans, as we predict an individualized prognostic outcome for METH addicts and devise brain region- and altered protein-specific therapies that reduce the METH-induced burden.

## Supporting Information

S1 Fig A heat map of the differential Olfactory bulb vs. hippocampus proteome along with the Protein Class analysis. Differential analysis of the protein class indicate the upregulation of apoptotic, cytoskeletal and chaperone proteins in the hippocampus compared to the differential changes in the protease, oxidoreductase and hydrolase enzymes detected in the olfactory bulb brain regions. This analysis was conducted utilizing R software and panther database bioinformatics software.
(TIF)

S1 Table Entire identified proteins list and quantitative spectral counts value of METH-treated hippocampal tissues and control. (PDF)

S2 Table Number of identified unique peptides corresponding to each identified protein of METH-treated hippocampal tissues and control. (PDF)

S3 Table Entire identified proteins list and quantitative spectral counts value of METH-treated olfactory bulb tissues and control. (PDF)

S4 Table Number of identified unique peptides corresponding to each identified protein of METH-treated olfactory bulb tissues and control. (PDF)

S5 Table Target proteins and transitions of the hippcampal proteome. (PDF)

S6 Table Target proteins and transitions of olfactory bulb proteome. (PDF)

S7 Table Table of annotations, indicating the relationship and regulation type among proteins. (PDF)

## Abbreviations

DAT: Dopamine transporter
METH: Methamphetamine
OB: Olfactory bulb
Hipp: Hippocampus
MRM: multiple reactions monitoring
IAA: iodoacetamide
