# Supplementary figures and images for: The Effect of Chronic Methamphetamine Exposure on the Hippocampal and Olfactory Bulb Neuroproteomes of Rats
Source: PLoS One. 2016 Apr 15;11(4):e0151034. doi: 10.1371/journal.pone.0151034 (PMC4833297; doi:10.1371/journal.pone.0151034)

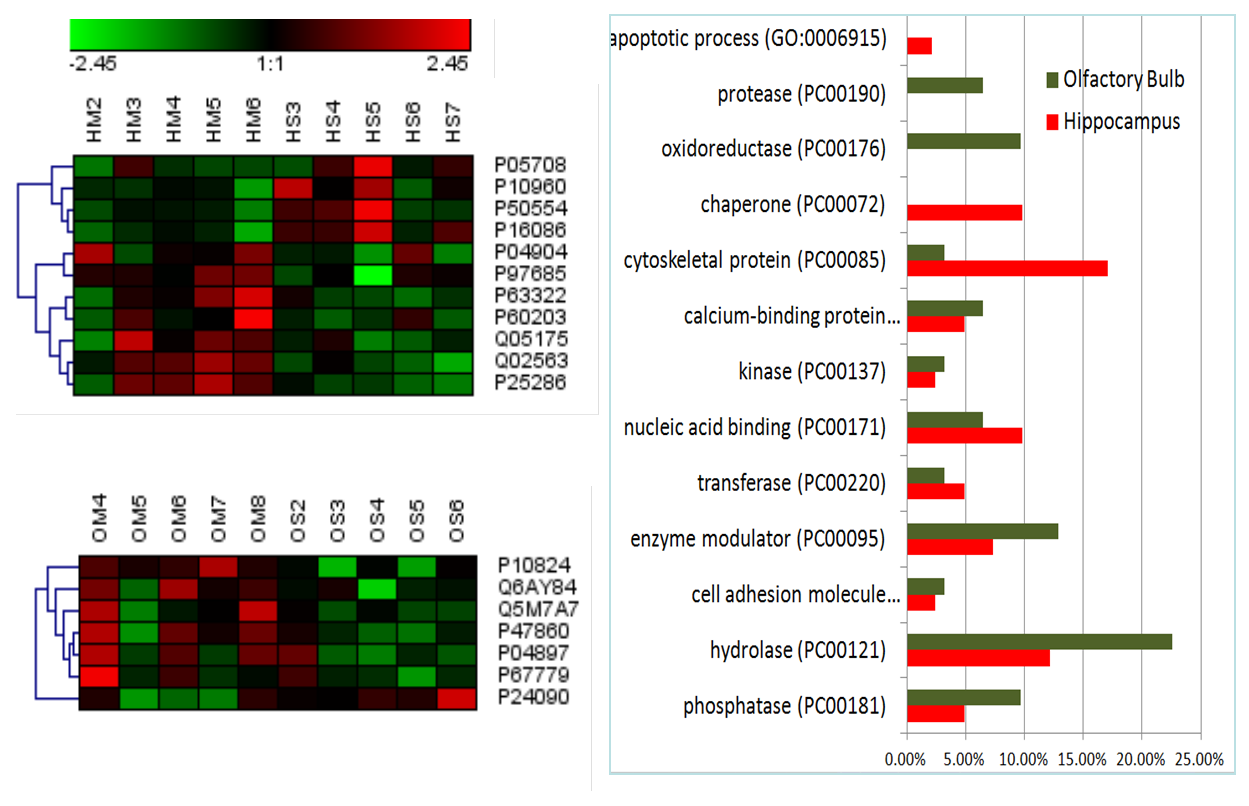

Supplement: S1 Fig — Differential analysis of the protein class indicate the upregulation of apoptotic, cytoskeletal and chaperone proteins in the hippocampus compared to the differential changes in the protease, oxidoreductase and hydrolase enzymes detected in the olfactory bulb brain regions. This analysis was conducted utilizing R software and panther database bioinformatics software. (TIF) [file pone.0151034.s001.tif]
