# Supplementary material for: The Effect of Chronic Methamphetamine Exposure on the Hippocampal and Olfactory Bulb Neuroproteomes of Rats
Source: PLoS One. 2016 Apr 15;11(4):e0151034. doi: 10.1371/journal.pone.0151034 (PMC4833297; doi:10.1371/journal.pone.0151034)
Supplement: S1 Table — (PDF) [file pone.0151034.s002.pdf]

Table S1. Entire identified proteins list and quantitative spectra counts value of METH treated hippocampal tissues and control

	Quantitative value of spectra counts								
	METH treated Hipp samples					Control			
Identified Proteins (302)	HM2	HM3	HM4	HM5	HM6	HS3	HS4	HS6	HS7
Spectrin alpha chain, non-erythrocytic 1 OS=Rattus norvegicus GN=Sptan1 PE=1 SV=2	47.75	43.17	45.16	52.30	41.15	45.98	55.91	56.22	59.65
Tubulin alpha-1B chain OS=Rattus norvegicus GN=Tuba1b PE=1 SV=1	32.47	27.75	39.52	29.61	31.11	41.38	46.37	34.52	45.67
Tubulin beta-2B chain OS=Rattus norvegicus GN=Tubb2b PE=1 SV=1	40.11	31.87	42.34	38.49	46.16	41.38	47.73	37.48	39.15
Clathrin heavy chain 1 OS=Rattus norvegicus GN=Cltc PE=1 SV=3	43.93	28.78	30.11	37.50	28.10	33.10	38.19	35.51	15.85
Hemoglobin subunit alpha-1/2 OS=Rattus norvegicus GN=Hba1 PE=1 SV=3	37.25	50.37	32.93	32.57	28.10	32.18	23.18	44.38	38.21
Actin, cytoplasmic 1 OS=Rattus norvegicus GN=Actb PE=1 SV=1	26.74	35.98	35.76	30.59	32.11	33.10	30.00	27.62	29.83
Sodium/potassium-transporting ATPase subunit alpha-3 OS=Rattus norvegicus GN=Atp1a3 PE=1 SV=2	22.92	34.95	30.11	29.61	42.15	33.10	36.82	28.60	35.42
Dihydropyrimidinase-related protein 2 OS=Rattus norvegicus GN=Dpysl2 PE=1 SV=1	20.06	14.39	20.70	26.65	20.07	22.99	23.18	24.66	22.37
ATP synthase subunit beta, mitochondrial OS=Rattus norvegicus GN=Atp5b PE=1 SV=2	28.65	21.59	28.23	24.67	33.12	22.07	20.46	26.63	26.10
Microtubule-associated protein 2 OS=Rattus norvegicus GN=Map2 PE=1 SV=3	25.79	26.73	26.35	19.74	26.09	19.31	21.82	25.64	22.37
Hemoglobin subunit beta-1 OS=Rattus norvegicus GN=Hbb PE=1 SV=3	19.10	23.64	21.64	24.67	21.08	17.47	25.91	25.64	26.10
Tubulin alpha-1A chain OS=Rattus norvegicus GN=Tuba1a PE=1 SV=1	39.16	41.12	41.40	36.51	32.11	42.30	49.10	40.44	53.13
Creatine kinase B-type OS=Rattus norvegicus GN=Ckb PE=1 SV=2	20.06	18.50	10.35	12.83	16.06	13.79	10.91	13.81	13.05
Serum albumin OS=Rattus norvegicus GN=Alb PE=1 SV=2	19.10	27.75	31.05	16.78	23.08	21.15	16.37	31.56	20.51
Heat shock cognate 71 kDa protein OS=Rattus norvegicus GN=Hspa8 PE=1 SV=1	21.97	23.64	21.64	22.70	21.08	24.83	27.28	23.67	23.30
Pyruvate kinase isozymes M1/M2 OS=Rattus norvegicus GN=Pkm PE=1 SV=3	18.15	21.59	27.29	22.70	24.09	22.99	20.46	24.66	17.71
Heat shock protein HSP 90-alpha OS=Rattus norvegicus GN=Hsp90aa1 PE=1 SV=3	12.42	18.50	20.70	15.79	16.06	13.79	25.91	12.82	16.78
Aconitate hydratase, mitochondrial OS=Rattus norvegicus GN=Aco2 PE=1 SV=2	15.28	15.42	14.11	23.69	14.05	17.47	19.09	20.71	16.78
2',3'-cyclic-nucleotide 3'-phosphodiesterase OS=Rattus norvegicus GN=Cnp PE=1 SV=2	16.24	16.45	16.94	18.75	21.08	18.39	16.37	16.77	12.12
Alpha-internexin OS=Rattus norvegicus GN=Ina PE=1 SV=2	17.19	18.50	16.94	17.76	14.05	22.99	21.82	21.70	20.51
Dynamin-1 OS=Rattus norvegicus GN=Dnm1 PE=1 SV=2	13.37	16.45	17.88	15.79	16.06	16.55	17.73	15.78	19.57
Microtubule-associated protein 1B OS=Rattus norvegicus GN=Map1b PE=1 SV=2	10.51	17.48	14.11	11.84	14.05	12.87	15.00	17.75	20.51
Gamma-enolase OS=Rattus norvegicus GN=Eno2 PE=1 SV=2	16.24	12.34	14.11	15.79	16.06	13.79	15.00	16.77	13.05
Syntaxin-binding protein 1 OS=Rattus norvegicus GN=Stxbp1 PE=1 SV=1	8.60	16.45	13.17	13.82	9.03	18.39	15.00	13.81	17.71
ATP synthase subunit alpha, mitochondrial OS=Rattus norvegicus GN=Atp5a1 PE=1 SV=2	14.33	19.53	16.00	13.82	10.04	11.03	21.82	11.84	13.05
14-3-3 protein zeta/delta OS=Rattus norvegicus GN=Ywhaz PE=1 SV=1	18.15	17.48	14.11	19.74	17.06	21.15	19.09	17.75	17.71
Vesicle-fusing ATPase OS=Rattus norvegicus GN=Nsf PE=1 SV=1	12.42	12.34	14.11	12.83	15.05	14.71	16.37	12.82	14.91
Spectrin beta chain, non-erythrocytic 2 OS=Rattus norvegicus GN=Sptbn2 PE=1 SV=2	9.55	15.42	9.41	7.89	7.03	9.20	10.91	10.85	9.32
14-3-3 protein epsilon OS=Rattus norvegicus GN=Ywhae PE=1 SV=1	10.51	12.34	13.17	14.80	11.04	10.12	13.64	14.80	10.25
Synapsin-1 OS=Rattus norvegicus GN=Syn1 PE=1 SV=3	10.51	8.22	9.41	12.83	10.04	11.03	12.27	11.84	13.98
Calcium/calmodulin-dependent protein kinase type II subunit alpha OS=Rattus norvegicus GN=Camk2a PE=1 SV=1	7.64	11.31	11.29	7.89	16.06	7.36	10.91	11.84	12.12
Glial fibrillary acidic protein OS=Rattus norvegicus GN=Gfap PE=1 SV=2	14.33	8.22	11.29	10.86	14.05	12.87	15.00	14.80	15.85
Hexokinase-1 OS=Rattus norvegicus GN=Hk1 PE=1 SV=4	14.33	10.28	10.35	13.82	9.03	11.95	6.82	8.88	10.25
Fructose-bisphosphate aldolase A OS=Rattus norvegicus GN=Aldoa PE=1 SV=2	13.37	14.39	10.35	10.86	12.04	11.95	16.37	12.82	11.18
Malate dehydrogenase, mitochondrial OS=Rattus norvegicus GN=Mdh2 PE=1 SV=2	10.51	10.28	13.17	10.86	12.04	11.03	16.37	12.82	7.46
Ras-related protein Rab-3A OS=Rattus norvegicus GN=Rab3a PE=1 SV=1	8.60	13.36	7.53	12.83	10.04	12.87	9.55	7.89	9.32
Microtubule-associated protein 1A OS=Rattus norvegicus GN=Map1a PE=1 SV=1	6.69	6.17	7.53	6.91	10.04	4.60	6.82	8.88	7.46
Calmodulin OS=Rattus norvegicus GN=Calm1 PE=1 SV=2	6.69	11.31	9.41	9.87	12.04	8.28	8.18	10.85	10.25
Rab GDP dissociation inhibitor alpha OS=Rattus norvegicus GN=Gdi1 PE=1 SV=1	8.60	11.31	11.29	7.89	8.03	8.28	9.55	12.82	10.25
Glyceraldehyde-3-phosphate dehydrogenase OS=Rattus norvegicus GN=Gapdh PE=1 SV=3	8.60	5.14	9.41	7.89	9.03	11.03	9.55	4.93	10.25
Aspartate aminotransferase, cytoplasmic OS=Rattus norvegicus GN=Got1 PE=1 SV=3	8.60	9.25	7.53	7.89	5.02	9.20	8.18	8.88	6.52
Synaptotagmin-1 OS=Rattus norvegicus GN=Syt1 PE=1 SV=3	5.73	9.25	9.41	6.91	8.03	7.36	8.18	9.86	2.80
Synapsin-2 OS=Rattus norvegicus GN=Syn2 PE=1 SV=1	7.64	3.08	8.47	5.92	7.03	5.52	9.55	3.95	6.52
Heat shock protein HSP 90-beta OS=Rattus norvegicus GN=Hsp90ab1 PE=1 SV=4	9.55	11.31	12.23	13.82	7.03	7.36	20.46	8.88	17.71
Ubiquitin carboxyl-terminal hydrolase isozyme L1 OS=Rattus norvegicus GN=Uchl1 PE=1 SV=2	10.51	9.25	10.35	9.87	11.04	9.20	15.00	8.88	8.39

	Quantitative value of spectra counts								
	METH treated Hipp samples					Control			
Identified Proteins (302)	HM2	HM3	HM4	HM5	HM6	HS3	HS4	HS6	HS7
Guanine nucleotide-binding protein G(o) subunit alpha OS=Rattus norvegicus GN=Gnao1 PE=1 SV=2	7.64	7.20	8.47	8.88	8.03	6.44	13.64	8.88	7.46
Triosephosphate isomerase OS=Rattus norvegicus GN=Tpi1 PE=1 SV=2	8.60	9.25	7.53	7.89	7.03	11.95	16.37	5.92	9.32
Neurofilament light polypeptide OS=Rattus norvegicus GN=Nefl PE=1 SV=3	12.42	13.36	9.41	9.87	9.03	10.12	15.00	8.88	17.71
AP-2 complex subunit beta OS=Rattus norvegicus GN=Ap2b1 PE=1 SV=1	10.51	7.20	9.41	13.82	9.03	7.36	6.82	10.85	6.52
Malate dehydrogenase, cytoplasmic OS=Rattus norvegicus GN=Mdh1 PE=1 SV=3	7.64	8.22	9.41	8.88	10.04	7.36	6.82	7.89	8.39
Pyruvate dehydrogenase E1 component subunit beta, mitochondrial OS=Rattus norvegicus GN=Pdhb PE=1 SV=2	6.69	9.25	7.53	4.93	10.04	5.52	6.82	2.96	8.39
Alpha-enolase OS=Rattus norvegicus GN=Eno1 PE=1 SV=4	11.46	12.34	15.06	14.80	15.05	14.71	15.00	15.78	12.12
Tubulin beta-3 chain OS=Rattus norvegicus GN=Tubb3 PE=1 SV=1	25.79	25.70	25.41	27.63	29.10	22.07	25.91	27.62	18.64
14-3-3 protein beta/alpha OS=Rattus norvegicus GN=Ywhab PE=1 SV=3	11.46	12.34	11.29	13.82	14.05	10.12	10.91	10.85	11.18
Sodium/potassium-transporting ATPase subunit beta-1 OS=Rattus norvegicus GN=Atp1b1 PE=1 SV=1	0.96	1.03	6.59	6.91	4.01	3.68	4.09	5.92	6.52
Excitatory amino acid transporter 2 OS=Rattus norvegicus GN=Slc1a2 PE=1 SV=2	7.64	11.31	8.47	5.92	9.03	7.36	6.82	9.86	6.52
Phosphoglycerate kinase 1 OS=Rattus norvegicus GN=Pgk1 PE=1 SV=2	6.69	7.20	6.59	5.92	4.01	8.28	9.55	4.93	8.39
Glucose-6-phosphate isomerase OS=Rattus norvegicus GN=Gpi PE=1 SV=1	5.73	5.14	4.70	3.95	8.03	3.68	4.09	4.93	5.59
60 kDa heat shock protein, mitochondrial OS=Rattus norvegicus GN=Hspd1 PE=1 SV=1	7.64	4.11	5.65	1.97	4.01	6.44	5.46	11.84	10.25
Cofilin-1 OS=Rattus norvegicus GN=Cfl1 PE=1 SV=3	6.69	5.14	6.59	6.91	10.04	8.28	9.55	5.92	8.39
NAD-dependent protein deacetylase sirtuin-2 OS=Rattus norvegicus GN=Sirt2 PE=1 SV=1	5.73	8.22	8.47	8.88	6.02	7.36	1.36	7.89	7.46
Fructose-bisphosphate aldolase C OS=Rattus norvegicus GN=Aldoc PE=1 SV=3	7.64	9.25	6.59	6.91	7.03	9.20	5.46	7.89	5.59
Tenascin-R OS=Rattus norvegicus GN=Tnr PE=1 SV=1	4.78	8.22	7.53	9.87	4.01	4.60	8.18	3.95	10.25
Cytochrome b-c1 complex subunit 1, mitochondrial OS=Rattus norvegicus GN=Uqcrc1 PE=1 SV=1	5.73	8.22	5.65	4.93	8.03	9.20	5.46	4.93	3.73
Peptidyl-prolyl cis-trans isomerase A OS=Rattus norvegicus GN=Ppia PE=1 SV=2	11.46	8.22	5.65	7.89	9.03	6.44	5.46	3.95	7.46
Brain acid soluble protein 1 OS=Rattus norvegicus GN=Basp1 PE=1 SV=2	5.73	5.14	5.65	6.91	7.03	5.52	5.46	4.93	3.73
Alpha-actinin-1 OS=Rattus norvegicus GN=Actn1 PE=1 SV=1	3.82	3.08	5.65	1.97	5.02	7.36	2.73	4.93	8.39
Elongation factor 1-alpha 1 OS=Rattus norvegicus GN=Eef1a1 PE=1 SV=1	4.78	5.14	4.70	4.93	7.03	5.52	8.18	10.85	5.59
V-type proton ATPase subunit B, brain isoform OS=Rattus norvegicus GN=Atp6v1b2 PE=1 SV=1	9.55	8.22	4.70	6.91	9.03	5.52	2.73	3.95	1.86
Neurochondrin OS=Rattus norvegicus GN=Ncdn PE=1 SV=2	5.73	7.20	6.59	7.89	9.03	3.68	2.73	6.90	3.73
Myelin proteolipid protein OS=Rattus norvegicus GN=Plp1 PE=1 SV=2	6.69	6.17	10.35	7.89	11.04	7.36	4.09	6.90	7.46
Beta-soluble NSF attachment protein OS=Rattus norvegicus GN=Napb PE=1 SV=1	4.78	4.11	5.65	6.91	5.02	6.44	5.46	4.93	8.39
Neurofilament medium polypeptide OS=Rattus norvegicus GN=Nefm PE=1 SV=4	5.73	5.14	7.53	9.87	8.03	11.03	9.55	7.89	9.32
L-lactate dehydrogenase A chain OS=Rattus norvegicus GN=Ldha PE=1 SV=1	4.78	10.28	2.82	3.95	3.01	5.52	8.18	5.92	6.52
Guanine nucleotide-binding protein G(I)/G(S)/G(T) subunit beta-1 OS=Rattus norvegicus GN=Gnb1 PE=1 SV=4	4.78	3.08	1.88	3.95	4.01	7.36	2.73	5.92	5.59
Glycogen phosphorylase, brain form (Fragment) OS=Rattus norvegicus GN=Pygb PE=1 SV=3	6.69	9.25	2.82	4.93	6.02	5.52	1.36	1.97	6.52
Tubulin beta-5 chain OS=Rattus norvegicus GN=Tubb5 PE=1 SV=1	41.07	34.95	40.46	41.45	43.15	37.70	40.91	40.44	37.28
14-3-3 protein theta OS=Rattus norvegicus GN=Ywhaq PE=1 SV=1	10.51	10.28	8.47	12.83	11.04	10.12	9.55	14.80	11.18
AP-2 complex subunit alpha-2 OS=Rattus norvegicus GN=Ap2a2 PE=1 SV=3	5.73	6.17	4.70	5.92	4.01	7.36	4.09	5.92	1.86
NADH-ubiquinone oxidoreductase 75 kDa subunit, mitochondrial OS=Rattus norvegicus GN=Ndufs1 PE=1 SV=1	3.82	3.08	6.59	5.92	7.03	5.52	2.73	8.88	5.59
14-3-3 protein eta OS=Rattus norvegicus GN=Ywhah PE=1 SV=2	10.51	14.39	9.41	10.86	11.04	9.20	9.55	11.84	10.25
Visinin-like protein 1 OS=Rattus norvegicus GN=Vsnl1 PE=1 SV=2	6.69	8.22	7.53	8.88	5.02	6.44	5.46	5.92	5.59
Myelin basic protein S OS=Rattus norvegicus GN=Mbp PE=1 SV=3	4.78	2.06	2.82	1.97	11.04	4.60	4.09	3.95	1.86
Aspartate aminotransferase, mitochondrial OS=Rattus norvegicus GN=Got2 PE=1 SV=2	6.69	8.22	7.53	6.91	4.01	6.44	6.82	6.90	3.73
Neuron-specific calcium-binding protein hippocalcin OS=Rattus norvegicus GN=Hpca PE=1 SV=2	5.73	8.22	5.65	7.89	6.02	4.60	4.09	7.89	5.59
Myristoylated alanine-rich C-kinase substrate OS=Rattus norvegicus GN=Marcks PE=1 SV=2	7.64	3.08	6.59	5.92	6.02	6.44	9.55	4.93	3.73
Glutamine synthetase OS=Rattus norvegicus GN=Glul PE=1 SV=3	8.60	6.17	7.53	3.95	4.01	3.68	9.55	5.92	7.46
Amphiphysin OS=Rattus norvegicus GN=Amph PE=1 SV=1	4.78	6.17	3.76	3.95	4.01	8.28	8.18	3.95	4.66
Contactin-1 OS=Rattus norvegicus GN=Cntn1 PE=1 SV=2	0.00	5.14	3.76	4.93	5.02	3.68	2.73	2.96	0.93
Microtubule-associated protein tau OS=Rattus norvegicus GN=Mapt PE=1 SV=3	7.64	6.17	6.59	3.95	3.01	5.52	5.46	4.93	4.66
Septin-11 OS=Rattus norvegicus GN=Sept11 PE=1 SV=1	2.87	4.11	4.70	1.97	7.03	4.60	2.73	2.96	5.59

	Quantitative value of spectra counts								
	METH treated Hipp samples					Control			
Identified Proteins (302)	HM2	HM3	HM4	HM5	HM6	HS3	HS4	HS6	HS7
Serine/threonine-protein phosphatase 2B catalytic subunit alpha isoform OS=Rattus norvegicus GN=Ppp3ca PE=1 SV=1	7.64	3.08	3.76	4.93	3.01	8.28	5.46	8.88	3.73
Endophilin-A1 OS=Rattus norvegicus GN=Sh3gl2 PE=1 SV=2	4.78	0.00	4.70	2.96	3.01	4.60	1.36	2.96	4.66
78 kDa glucose-regulated protein OS=Rattus norvegicus GN=Hspa5 PE=1 SV=1	11.46	10.28	7.53	7.89	7.03	7.36	13.64	6.90	8.39
Phosphatidylethanolamine-binding protein 1 OS=Rattus norvegicus GN=Pebp1 PE=1 SV=3	7.64	7.20	6.59	6.91	3.01	4.60	6.82	4.93	5.59
Peroxiredoxin-6 OS=Rattus norvegicus GN=Prdx6 PE=1 SV=3	5.73	6.17	1.88	2.96	2.01	6.44	5.46	4.93	5.59
Brevican core protein OS=Rattus norvegicus GN=Bcan PE=1 SV=2	2.87	5.14	3.76	5.92	2.01	2.76	5.46	2.96	6.52
ATP synthase-coupling factor 6, mitochondrial OS=Rattus norvegicus GN=Atp5j PE=1 SV=1	3.82	4.11	4.70	5.92	4.01	5.52	4.09	4.93	3.73
Astrocytic phosphoprotein PEA-15 OS=Rattus norvegicus GN=Pea15 PE=1 SV=1	3.82	3.08	2.82	3.95	3.01	1.84	4.09	5.92	3.73
V-type proton ATPase subunit C 1 OS=Rattus norvegicus GN=Atp6v1c1 PE=2 SV=1	4.78	4.11	4.70	6.91	4.01	7.36	0.00	5.92	3.73
Protein kinase C and casein kinase substrate in neurons protein 1 OS=Rattus norvegicus GN=Pacsin1 PE=1 SV=1	7.64	4.11	2.82	5.92	5.02	3.68	4.09	4.93	3.73
Calreticulin OS=Rattus norvegicus GN=Calr PE=1 SV=1	2.87	1.03	4.70	4.93	2.01	1.84	4.09	3.95	1.86
Tubulin alpha-4A chain OS=Rattus norvegicus GN=Tuba4a PE=2 SV=1	29.61	24.67	33.87	27.63	29.10	39.54	40.91	32.55	40.08
Glutamate dehydrogenase 1, mitochondrial OS=Rattus norvegicus GN=Glud1 PE=1 SV=2	6.69	6.17	5.65	2.96	5.02	4.60	2.73	3.95	7.46
Calcineurin subunit B type 1 OS=Rattus norvegicus GN=Ppp3r1 PE=1 SV=2	4.78	5.14	2.82	0.00	1.00	2.76	4.09	0.00	1.86
Citrate synthase, mitochondrial OS=Rattus norvegicus GN=Cs PE=1 SV=1	4.78	4.11	2.82	3.95	3.01	5.52	8.18	5.92	2.80
Neuromodulin OS=Rattus norvegicus GN=Gap43 PE=1 SV=1	6.69	4.11	3.76	2.96	2.01	3.68	4.09	2.96	1.86
Cullin-associated NEDD8-dissociated protein 1 OS=Rattus norvegicus GN=Cand1 PE=1 SV=1	3.82	1.03	4.70	3.95	3.01	3.68	1.36	0.99	1.86
Heterogeneous nuclear ribonucleoprotein K OS=Rattus norvegicus GN=Hnrnpk PE=1 SV=1	5.73	3.08	4.70	5.92	6.02	2.76	1.36	1.97	2.80
Neuronal membrane glycoprotein M6-a OS=Rattus norvegicus GN=Gpm6a PE=1 SV=1	3.82	4.11	2.82	4.93	3.01	1.84	5.46	3.95	4.66
Tropomyosin alpha-3 chain OS=Rattus norvegicus GN=Tpm3 PE=1 SV=2	5.73	5.14	5.65	5.92	6.02	5.52	1.36	4.93	3.73
Tubulin beta-4B chain OS=Rattus norvegicus GN=Tubb4b PE=1 SV=1	41.07	33.92	39.52	42.44	42.15	37.70	40.91	33.54	40.08
Ubiquitin-like modifier-activating enzyme 1 OS=Rattus norvegicus GN=Uba1 PE=1 SV=1	3.82	2.06	3.76	4.93	4.01	6.44	4.09	0.99	3.73
Synaptophysin OS=Rattus norvegicus GN=Syp PE=1 SV=1	2.87	4.11	5.65	3.95	2.01	5.52	1.36	1.97	0.93
L-lactate dehydrogenase B chain OS=Rattus norvegicus GN=Ldhb PE=1 SV=2	4.78	7.20	5.65	5.92	4.01	7.36	2.73	5.92	3.73
Coactosin-like protein OS=Rattus norvegicus GN=Cotl1 PE=1 SV=1	3.82	2.06	0.94	1.97	5.02	1.84	1.36	1.97	0.93
6-phosphofructokinase, muscle type OS=Rattus norvegicus GN=Pfkm PE=2 SV=3	2.87	2.06	2.82	4.93	4.01	3.68	1.36	3.95	4.66
Superoxide dismutase [Cu-Zn] OS=Rattus norvegicus GN=Sod1 PE=1 SV=2	3.82	5.14	3.76	2.96	3.01	3.68	2.73	4.93	1.86
10 kDa heat shock protein, mitochondrial OS=Rattus norvegicus GN=Hspe1 PE=1 SV=3	5.73	4.11	1.88	2.96	2.01	3.68	4.09	3.95	2.80
Neural cell adhesion molecule 1 OS=Rattus norvegicus GN=Ncam1 PE=1 SV=1	1.91	2.06	3.76	5.92	5.02	1.84	2.73	5.92	1.86
Glutaminase kidney isoform, mitochondrial OS=Rattus norvegicus GN=Gls PE=1 SV=2	4.78	1.03	2.82	0.00	1.00	2.76	0.00	3.95	0.00
Syntaxin-1B OS=Rattus norvegicus GN=Stx1b PE=1 SV=1	3.82	4.11	4.70	2.96	3.01	1.84	5.46	2.96	0.93
Guanine deaminase OS=Rattus norvegicus GN=Gda PE=1 SV=1	5.73	5.14	3.76	1.97	4.01	2.76	4.09	4.93	5.59
Voltage-dependent anion-selective channel protein 1 OS=Rattus norvegicus GN=Vdac1 PE=1 SV=4	2.87	4.11	4.70	5.92	6.02	4.60	2.73	2.96	4.66
Serotransferrin OS=Rattus norvegicus GN=Tf PE=1 SV=3	1.91	7.20	4.70	2.96	1.00	1.84	1.36	7.89	3.73
Cytochrome b-c1 complex subunit 2, mitochondrial OS=Rattus norvegicus GN=Uqcrc2 PE=1 SV=2	4.78	5.14	3.76	4.93	4.01	4.60	1.36	0.99	3.73
Vesicle-associated membrane protein 2 OS=Rattus norvegicus GN=Vamp2 PE=1 SV=2	4.78	5.14	4.70	3.95	4.01	5.52	5.46	4.93	2.80
Microtubule-associated protein 6 OS=Rattus norvegicus GN=Map6 PE=1 SV=1	0.96	3.08	3.76	2.96	3.01	5.52	1.36	2.96	4.66
Peroxiredoxin-5, mitochondrial OS=Rattus norvegicus GN=Prdx5 PE=1 SV=1	2.87	2.06	5.65	2.96	3.01	4.60	4.09	3.95	3.73
Isocitrate dehydrogenase [NAD] subunit beta, mitochondrial OS=Rattus norvegicus GN=Idh3B PE=1 SV=1	1.91	0.00	3.76	3.95	3.01	3.68	1.36	3.95	4.66
Thy-1 membrane glycoprotein OS=Rattus norvegicus GN=Thy1 PE=1 SV=1	1.91	3.08	2.82	3.95	3.01	5.52	5.46	1.97	3.73
V-type proton ATPase 116 kDa subunit a isoform 1 OS=Rattus norvegicus GN=Atp6v0a1 PE=2 SV=1	2.87	3.08	0.94	2.96	5.02	0.92	2.73	0.99	0.00
V-type proton ATPase subunit E 1 OS=Rattus norvegicus GN=Atp6v1e1 PE=1 SV=1	3.82	4.11	3.76	1.97	2.01	2.76	5.46	2.96	3.73
Dihydropyrimidinase-related protein 1 OS=Rattus norvegicus GN=Crmp1 PE=1 SV=1	4.78	4.11	6.59	5.92	4.01	6.44	6.82	6.90	7.46
Dihydrolipoyl dehydrogenase, mitochondrial OS=Rattus norvegicus GN=Dld PE=1 SV=1	2.87	4.11	2.82	1.97	1.00	2.76	1.36	0.00	2.80
4-aminobutyrate aminotransferase, mitochondrial OS=Rattus norvegicus GN=Abat PE=1 SV=3	4.78	4.11	3.76	4.93	4.01	3.68	0.00	3.95	1.86
Drebrin OS=Rattus norvegicus GN=Dbn1 PE=2 SV=3	2.87	3.08	4.70	2.96	2.01	3.68	0.00	1.97	1.86

	Quantitative value of spectra counts								
	METH treated Hipp samples					Control			
Identified Proteins (302)	HM2	HM3	HM4	HM5	HM6	HS3	HS4	HS6	HS7
Fascin OS=Rattus norvegicus GN=Fscn1 PE=1 SV=2	2.87	3.08	1.88	3.95	3.01	1.84	1.36	1.97	3.73
Carbonic anhydrase 2 OS=Rattus norvegicus GN=Ca2 PE=1 SV=2	2.87	2.06	2.82	2.96	2.01	2.76	2.73	3.95	2.80
Synaptosomal-associated protein 25 OS=Rattus norvegicus GN=Snap25 PE=1 SV=1	3.82	4.11	4.70	2.96	6.02	2.76	5.46	1.97	1.86
Phosphoglycerate mutase 1 OS=Rattus norvegicus GN=Pgam1 PE=1 SV=4	2.87	3.08	2.82	0.99	3.01	2.76	6.82	1.97	3.73
Complement component 1 Q subcomponent-binding protein, mitochondrial OS=Rattus norvegicus GN=C1qbp PE=1 SV=2	1.91	2.06	4.70	2.96	4.01	2.76	4.09	2.96	2.80
Cytochrome c, somatic OS=Rattus norvegicus GN=Cycs PE=1 SV=2	3.82	3.08	2.82	2.96	3.01	5.52	5.46	2.96	3.73
Succinate dehydrogenase [ubiquinone] flavoprotein subunit, mitochondrial OS=Rattus norvegicus GN=Sdha PE=1 SV=1	1.91	1.03	2.82	2.96	3.01	2.76	2.73	5.92	1.86
Synaptojanin-1 OS=Rattus norvegicus GN=Synj1 PE=1 SV=3	1.91	2.06	0.94	1.97	1.00	4.60	4.09	1.97	0.00
2-oxoglutarate dehydrogenase, mitochondrial OS=Rattus norvegicus GN=Ogdh PE=1 SV=1	3.82	2.06	0.94	4.93	4.01	1.84	2.73	3.95	3.73
Sulfated glycoprotein 1 OS=Rattus norvegicus GN=Psap PE=1 SV=1	2.87	3.08	3.76	2.96	4.01	1.84	1.36	1.97	2.80
Creatine kinase U-type, mitochondrial OS=Rattus norvegicus GN=Ckmt1 PE=1 SV=1	2.87	3.08	2.82	2.96	4.01	2.76	4.09	4.93	2.80
Transketolase OS=Rattus norvegicus GN=Tkt PE=1 SV=1	0.96	1.03	0.94	2.96	2.01	1.84	0.00	0.99	1.86
14-3-3 protein gamma OS=Rattus norvegicus GN=Ywhag PE=1 SV=2	6.69	6.17	4.70	6.91	7.03	7.36	9.55	8.88	6.52
Histone H2A type 1-C OS=Rattus norvegicus PE=1 SV=2	3.82	4.11	2.82	1.97	4.01	1.84	1.36	1.97	3.73
Alpha-synuclein OS=Rattus norvegicus GN=Snca PE=1 SV=1	2.87	3.08	2.82	2.96	3.01	3.68	2.73	2.96	3.73
Hemoglobin subunit beta-2 OS=Rattus norvegicus PE=1 SV=2	17.19	18.50	16.94	17.76	16.06	13.79	17.73	18.74	15.85
Keratin, type I cytoskeletal 10 OS=Rattus norvegicus GN=Krt10 PE=2 SV=1	7.64	9.25	0.94	1.97	3.01	0.92	1.36	0.00	0.00
NADH dehydrogenase [ubiquinone] flavoprotein 2, mitochondrial OS=Rattus norvegicus GN=Ndufv2 PE=1 SV=2	5.73	3.08	1.88	1.97	2.01	3.68	1.36	0.99	1.86
Thiomorpholine-carboxylate dehydrogenase OS=Rattus norvegicus GN=Crym PE=1 SV=1	3.82	2.06	1.88	0.99	4.01	2.76	1.36	1.97	1.86
Transitional endoplasmic reticulum ATPase OS=Rattus norvegicus GN=Vcp PE=1 SV=3	1.91	3.08	1.88	2.96	2.01	2.76	2.73	1.97	4.66
Nucleoside diphosphate kinase B OS=Rattus norvegicus GN=Nme2 PE=1 SV=1	2.87	3.08	1.88	0.99	2.01	0.92	0.00	1.97	0.93
Ubiquitin-60S ribosomal protein L40 OS=Rattus norvegicus GN=Uba52 PE=1 SV=2	3.82	1.03	2.82	1.97	0.00	2.76	2.73	2.96	1.86
Ubiquitin thioesterase OTUB1 OS=Rattus norvegicus GN=Otub1 PE=1 SV=1	0.96	0.00	0.94	0.99	3.01	1.84	0.00	0.99	0.93
Band 4.1-like protein 1 OS=Rattus norvegicus GN=Epb41l1 PE=1 SV=1	3.82	2.06	0.94	1.97	2.01	1.84	0.00	1.97	0.93
Isocitrate dehydrogenase [NAD] subunit alpha, mitochondrial OS=Rattus norvegicus GN=Idh3a PE=1 SV=1	3.82	4.11	1.88	1.97	4.01	1.84	2.73	3.95	4.66
Clathrin light chain A OS=Rattus norvegicus GN=Clta PE=1 SV=1	1.91	2.06	2.82	2.96	3.01	2.76	1.36	1.97	4.66
Protein DJ-1 OS=Rattus norvegicus GN=Park7 PE=1 SV=1	2.87	2.06	0.94	1.97	2.01	3.68	0.00	1.97	1.86
Protein disulfide-isomerase A3 OS=Rattus norvegicus GN=Pdia3 PE=1 SV=2	4.78	3.08	4.70	1.97	1.00	1.84	0.00	0.99	2.80
Apolipoprotein E OS=Rattus norvegicus GN=Apoe PE=1 SV=2	2.87	3.08	3.76	2.96	3.01	2.76	1.36	0.99	3.73
Peroxiredoxin-1 OS=Rattus norvegicus GN=Prdx1 PE=1 SV=1	2.87	4.11	1.88	2.96	2.01	1.84	1.36	0.00	3.73
Synaptic vesicle glycoprotein 2A OS=Rattus norvegicus GN=Sv2a PE=1 SV=2	4.78	3.08	2.82	0.99	5.02	0.92	4.09	0.00	0.00
Ras-related protein Rap-1A OS=Rattus norvegicus GN=Rap1a PE=1 SV=1	2.87	2.06	1.88	3.95	3.01	1.84	2.73	2.96	1.86
Elongation factor 2 OS=Rattus norvegicus GN=Eef2 PE=1 SV=4	2.87	2.06	1.88	2.96	2.01	2.76	0.00	1.97	1.86
Ras-related protein Rab-2A OS=Rattus norvegicus GN=Rab2a PE=1 SV=1	2.87	4.11	2.82	1.97	2.01	0.92	4.09	0.99	2.80
ADP-ribosylation factor 1 OS=Rattus norvegicus GN=Arf1 PE=1 SV=2	3.82	1.03	1.88	5.92	1.00	3.68	4.09	1.97	2.80
F-actin-capping protein subunit alpha-2 OS=Rattus norvegicus GN=Capza2 PE=1 SV=1	0.96	2.06	2.82	2.96	2.01	2.76	1.36	1.97	1.86
ProSAAS OS=Rattus norvegicus GN=Pcsk1n PE=1 SV=1	2.87	1.03	2.82	1.97	3.01	2.76	4.09	3.95	2.80
Heat shock 70 kDa protein 4 OS=Rattus norvegicus GN=Hspa4 PE=1 SV=1	1.91	3.08	2.82	2.96	1.00	2.76	0.00	2.96	1.86
Synaptogyrin-1 OS=Rattus norvegicus GN=Syngr1 PE=2 SV=1	3.82	2.06	3.76	2.96	4.01	1.84	2.73	3.95	1.86
Sodium/potassium-transporting ATPase subunit alpha-2 OS=Rattus norvegicus GN=Atp1a2 PE=1 SV=1	13.37	21.59	17.88	15.79	24.09	14.71	17.73	15.78	19.57
WD repeat-containing protein 1 OS=Rattus norvegicus GN=Wdr1 PE=1 SV=3	1.91	1.03	1.88	1.97	0.00	1.84	2.73	4.93	3.73
Myelin-associated glycoprotein OS=Rattus norvegicus GN=Mag PE=1 SV=1	0.96	2.06	2.82	2.96	2.01	2.76	0.00	4.93	1.86
ATP synthase subunit gamma, mitochondrial OS=Rattus norvegicus GN=Atp5c1 PE=1 SV=2	1.91	0.00	2.82	0.99	1.00	0.92	0.00	0.00	1.86
CaM kinase-like vesicle-associated protein OS=Rattus norvegicus GN=Camkv PE=1 SV=1	0.96	2.06	1.88	1.97	2.01	1.84	0.00	4.93	2.80
Phytanoyl-CoA hydroxylase-interacting protein OS=Rattus norvegicus GN=Phyhip PE=2 SV=1	0.96	0.00	2.82	0.99	1.00	3.68	0.00	1.97	2.80
Clathrin coat assembly protein AP180 OS=Rattus norvegicus GN=Snap91 PE=1 SV=1	3.82	1.03	1.88	1.97	1.00	4.60	0.00	1.97	1.86

	Quantitative value of spectra counts								
	METH treated Hipp samples					Control			
Identified Proteins (302)	HM2	HM3	HM4	HM5	HM6	HS3	HS4	HS6	HS7
Dihydrolipoyllysine-residue acetyltransferase component of pyruvate dehydrogenase complex, mitochondrial OS=Rattus norvegicus GN=Dlat PE=1 SV=3	2.87	2.06	0.94	3.95	2.01	1.84	0.00	1.97	3.73
Calcium-dependent secretion activator 1 OS=Rattus norvegicus GN=Cadps PE=1 SV=1	0.00	0.00	0.94	0.00	0.00	2.76	1.36	2.96	0.93
Cytoplasmic dynein 1 heavy chain 1 OS=Rattus norvegicus GN=Dync1h1 PE=1 SV=1	0.96	2.06	1.88	0.99	3.01	0.92	0.00	2.96	0.93
Superoxide dismutase [Mn], mitochondrial OS=Rattus norvegicus GN=Sod2 PE=1 SV=2	2.87	4.11	2.82	1.97	0.00	1.84	2.73	1.97	1.86
Alpha-adducin OS=Rattus norvegicus GN=Add1 PE=1 SV=2	1.91	3.08	2.82	0.00	4.01	0.00	1.36	0.99	1.86
Limbic system-associated membrane protein OS=Rattus norvegicus GN=Lsamp PE=1 SV=1	0.00	0.00	0.94	1.97	3.01	0.92	1.36	1.97	0.00
ATP synthase subunit O, mitochondrial OS=Rattus norvegicus GN=Atp5o PE=1 SV=1	0.96	2.06	0.94	0.99	2.01	0.92	0.00	2.96	0.93
Histidine triad nucleotide-binding protein 1 OS=Rattus norvegicus GN=Hint1 PE=1 SV=5	0.96	2.06	0.94	1.97	0.00	0.92	1.36	1.97	0.00
Heterogeneous nuclear ribonucleoproteins A2/B1 OS=Rattus norvegicus GN=Hnrnpa2b1 PE=1 SV=1	3.82	4.11	2.82	2.96	5.02	1.84	2.73	0.99	0.00
Histone H2B type 1 OS=Rattus norvegicus PE=1 SV=2	1.91	1.03	1.88	0.99	3.01	1.84	0.00	0.00	1.86
60S acidic ribosomal protein P2 OS=Rattus norvegicus GN=Rplp2 PE=1 SV=2	2.87	3.08	2.82	1.97	3.01	1.84	2.73	1.97	2.80
ADP/ATP translocase 1 OS=Rattus norvegicus GN=Slc25a4 PE=1 SV=3	0.96	2.06	1.88	1.97	3.01	0.92	1.36	1.97	1.86
Serine/threonine-protein phosphatase 2A 55 kDa regulatory subunit B alpha isoform OS=Rattus norvegicus GN=Ppp2r2a PE=2 SV=1	2.87	1.03	1.88	3.95	1.00	2.76	0.00	3.95	1.86
Neurofilament heavy polypeptide OS=Rattus norvegicus GN=Nefh PE=1 SV=4	5.73	5.14	2.82	2.96	4.01	6.44	4.09	3.95	5.59
AP-2 complex subunit mu OS=Rattus norvegicus GN=Ap2m1 PE=1 SV=1	0.00	1.03	0.94	0.99	1.00	0.00	0.00	0.00	2.80
Sodium/potassium-transporting ATPase subunit alpha-1 OS=Rattus norvegicus GN=Atp1a1 PE=1 SV=1	11.46	17.48	14.11	11.84	18.06	11.95	17.73	14.80	18.64
Coronin-1A OS=Rattus norvegicus GN=Coro1a PE=1 SV=3	0.96	1.03	0.94	0.99	4.01	2.76	2.73	0.99	2.80
Complexin-2 OS=Rattus norvegicus GN=Cplx2 PE=1 SV=1	0.96	1.03	0.94	0.00	2.01	0.00	2.73	0.99	0.00
Succinate-semialdehyde dehydrogenase, mitochondrial OS=Rattus norvegicus GN=Aldh5a1 PE=1 SV=2	2.87	2.06	2.82	1.97	2.01	0.92	2.73	1.97	1.86
GTP-binding nuclear protein Ran OS=Rattus norvegicus GN=Ran PE=1 SV=3	0.96	2.06	1.88	1.97	2.01	1.84	1.36	1.97	0.93
Histone H4 OS=Rattus norvegicus GN=Hist1h4b PE=1 SV=2	1.91	2.06	3.76	1.97	3.01	1.84	1.36	1.97	1.86
Protein kinase C gamma type OS=Rattus norvegicus GN=Prkcg PE=1 SV=1	1.91	1.03	1.88	0.99	3.01	2.76	2.73	1.97	0.93
UMP-CMP kinase OS=Rattus norvegicus GN=Cmpk1 PE=1 SV=2	3.82	1.03	0.94	4.93	0.00	2.76	0.00	1.97	1.86
Receptor-type tyrosine-protein phosphatase zeta OS=Rattus norvegicus GN=Ptprz1 PE=1 SV=1	1.91	1.03	0.94	1.97	0.00	3.68	0.00	0.00	2.80
6-phosphofructokinase type C OS=Rattus norvegicus GN=Pfkp PE=1 SV=2	1.91	0.00	1.88	0.99	2.01	0.00	2.73	3.95	3.73
Heterogeneous nuclear ribonucleoprotein A3 OS=Rattus norvegicus GN=Hnrnpa3 PE=1 SV=1	0.96	2.06	3.76	1.97	4.01	2.76	0.00	1.97	0.00
Peroxiredoxin-2 OS=Rattus norvegicus GN=Prdx2 PE=1 SV=3	2.87	0.00	1.88	3.95	0.00	0.00	2.73	2.96	0.93
Serine/threonine-protein phosphatase PP1-gamma catalytic subunit OS=Rattus norvegicus GN=Ppp1cc PE=1 SV=1	0.00	1.03	0.94	1.97	3.01	0.92	4.09	0.00	1.86
Rho GDP-dissociation inhibitor 1 OS=Rattus norvegicus GN=Arhgdia PE=1 SV=1	2.87	1.03	1.88	0.99	1.00	0.00	4.09	0.00	2.80
Cell division control protein 42 homolog OS=Rattus norvegicus GN=Cdc42 PE=1 SV=2	0.96	1.03	0.94	1.97	3.01	1.84	0.00	0.00	0.93
Dynamin-1-like protein OS=Rattus norvegicus GN=Dnm1l PE=1 SV=1	2.87	1.03	1.88	2.96	2.01	2.76	0.00	0.99	0.93
Synaptic vesicle glycoprotein 2B OS=Rattus norvegicus GN=Sv2b PE=1 SV=1	2.87	2.06	1.88	0.99	0.00	1.84	1.36	1.97	0.00
Adenylyl cyclase-associated protein 1 OS=Rattus norvegicus GN=Cap1 PE=1 SV=3	0.96	1.03	0.00	1.97	0.00	1.84	0.00	0.99	2.80
Alpha-1-inhibitor 3 OS=Rattus norvegicus GN=A1i3 PE=1 SV=1	0.96	3.08	0.94	0.99	0.00	0.00	2.73	1.97	2.80
Septin-8 OS=Rattus norvegicus GN=Sept8 PE=1 SV=1	3.82	3.08	2.82	3.95	5.02	5.52	0.00	1.97	3.73
ATP synthase subunit delta, mitochondrial OS=Rattus norvegicus GN=Atp5d PE=1 SV=2	1.91	2.06	1.88	1.97	2.01	1.84	4.09	2.96	1.86
Calcium/calmodulin-dependent protein kinase type II subunit beta OS=Rattus norvegicus GN=Camk2b PE=1 SV=1	2.87	6.17	3.76	0.99	7.03	3.68	2.73	3.95	3.73
Rab GDP dissociation inhibitor beta OS=Rattus norvegicus GN=Gdi2 PE=1 SV=2	6.69	6.17	2.82	4.93	4.01	1.84	1.36	3.95	4.66
Adenylate kinase isoenzyme 1 OS=Rattus norvegicus GN=Ak1 PE=1 SV=3	2.87	2.06	0.94	1.97	2.01	1.84	0.00	0.00	2.80
Transgelin-3 OS=Rattus norvegicus GN=Tagln3 PE=1 SV=2	1.91	3.08	1.88	1.97	2.01	0.92	2.73	0.00	2.80
Lactoylglutathione lyase OS=Rattus norvegicus GN=Glo1 PE=1 SV=3	2.87	2.06	1.88	1.97	3.01	0.92	0.00	0.99	1.86
Excitatory amino acid transporter 1 OS=Rattus norvegicus GN=Slc1a3 PE=1 SV=2	1.91	3.08	0.00	1.97	4.01	1.84	0.00	1.97	0.00
Actin-related protein 2/3 complex subunit 1A OS=Rattus norvegicus GN=Arpc1a PE=2 SV=1	0.96	1.03	0.94	0.99	2.01	0.00	0.00	0.00	0.93
Platelet-activating factor acetylhydrolase IB subunit beta OS=Rattus norvegicus GN=Pafah1b2 PE=1 SV=1	0.96	0.00	1.88	1.97	1.00	2.76	1.36	0.99	0.00
Endoplasmin OS=Rattus norvegicus GN=Hsp90b1 PE=1 SV=2	4.78	2.06	3.76	2.96	3.01	0.92	4.09	1.97	3.73

Identified Proteins (302)	Quantitative value of spectra counts								
	METH treated Hipp samples					Control			
	HM2	HM3	HM4	HM5	HM6	HS3	HS4	HS6	HS7
Succinyl-CoA ligase [ADP/GDP-forming] subunit alpha, mitochondrial OS=Rattus norvegicus GN=Suclg1 PE=2 SV=2	1.91	1.03	2.82	1.97	2.01	1.84	1.36	0.99	1.86
Serine/threonine-protein phosphatase 2A catalytic subunit alpha isoform OS=Rattus norvegicus GN=Ppp2ca PE=1 SV=1	0.96	1.03	3.76	1.97	1.00	1.84	1.36	1.97	0.00
Protein NDRG2 OS=Rattus norvegicus GN=Ndrg2 PE=1 SV=1	2.87	1.03	1.88	0.00	0.00	1.84	0.00	0.99	1.86
Myc box-dependent-interacting protein 1 OS=Rattus norvegicus GN=Bin1 PE=1 SV=1	0.00	2.06	0.00	0.99	0.00	0.00	2.73	1.97	1.86
Protein phosphatase 1E OS=Rattus norvegicus GN=Ppm1e PE=2 SV=1	0.96	1.03	1.88	1.97	2.01	1.84	1.36	1.97	3.73
Actin-related protein 3 OS=Rattus norvegicus GN=Actr3 PE=1 SV=1	0.96	0.00	0.94	0.99	3.01	0.92	2.73	0.00	0.93
EF-hand domain-containing protein D2 OS=Rattus norvegicus GN=Efhd2 PE=1 SV=1	0.96	1.03	0.00	0.00	1.00	1.84	0.00	2.96	0.93
Ubiquitin-conjugating enzyme E2 N OS=Rattus norvegicus GN=Ube2n PE=1 SV=1	0.00	1.03	2.82	0.00	1.00	3.68	1.36	0.99	0.93
Peptidyl-prolyl cis-trans isomerase FKBP1A OS=Rattus norvegicus GN=Fkbp1a PE=1 SV=3	2.87	4.11	1.88	1.97	3.01	0.00	1.36	0.00	0.00
Rho-related GTP-binding protein RhoB OS=Rattus norvegicus GN=Rhob PE=1 SV=1	0.96	2.06	0.94	1.97	0.00	0.92	4.09	0.99	1.86
Pyruvate dehydrogenase E1 component subunit alpha, somatic form, mitochondrial OS=Rattus norvegicus GN=Pdha1 PE=1 SV=2	2.87	0.00	1.88	0.00	1.00	1.84	0.00	0.99	2.80
Cytochrome c oxidase subunit 4 isoform 1, mitochondrial OS=Rattus norvegicus GN=Cox4i1 PE=1 SV=1	0.00	0.00	0.00	1.97	0.00	1.84	1.36	0.00	2.80
Actin-related protein 2/3 complex subunit 2 OS=Rattus norvegicus GN=Arpc2 PE=1 SV=1	0.00	0.00	2.82	0.00	1.00	0.00	1.36	0.00	1.86
Neurofascin OS=Rattus norvegicus GN=Nfasc PE=1 SV=2	1.91	0.00	1.88	1.97	1.00	1.84	1.36	2.96	1.86
Septin-7 OS=Rattus norvegicus GN=Sept7 PE=1 SV=1	2.87	1.03	4.70	3.95	5.02	3.68	1.36	2.96	4.66
Alpha-centractin OS=Rattus norvegicus GN=Actr1a PE=1 SV=1	1.91	2.06	2.82	1.97	2.01	1.84	0.00	0.00	0.00
Heterogeneous nuclear ribonucleoprotein D0 OS=Rattus norvegicus GN=Hnrnpd PE=1 SV=1	1.91	3.08	2.82	1.97	4.01	0.92	0.00	0.00	0.93
Guanine nucleotide-binding protein G(I)/G(S)/G(T) subunit beta-2 OS=Rattus norvegicus GN=Gnb2 PE=1 SV=4	4.78	3.08	1.88	1.97	3.01	7.36	0.00	3.95	3.73
Stress-70 protein, mitochondrial OS=Rattus norvegicus GN=Hspa9 PE=1 SV=3	0.00	2.06	0.00	1.97	0.00	0.00	4.09	1.97	0.00
Calbindin OS=Rattus norvegicus GN=Calb1 PE=1 SV=2	1.91	3.08	0.94	0.99	2.01	0.92	1.36	0.99	0.93
Growth factor receptor-bound protein 2 OS=Rattus norvegicus GN=Grb2 PE=1 SV=1	0.96	1.03	2.82	1.97	2.01	0.92	0.00	0.99	0.00
Oxidation resistance protein 1 OS=Rattus norvegicus GN=Oxr1 PE=1 SV=3	1.91	0.00	0.00	1.97	1.00	1.84	1.36	0.99	3.73
40S ribosomal protein SA OS=Rattus norvegicus GN=Rpsa PE=1 SV=3	1.91	2.06	0.94	1.97	1.00	1.84	1.36	1.97	0.00
1-phosphatidylinositol 4,5-bisphosphate phosphodiesterase beta-1 OS=Rattus norvegicus GN=Plcb1 PE=1 SV=1	1.91	0.00	1.88	0.00	1.00	1.84	1.36	0.99	2.80
Protein IMPACT OS=Rattus norvegicus GN=Impact PE=2 SV=1	0.96	0.00	2.82	1.97	0.00	0.92	0.00	0.00	1.86
Calnexin OS=Rattus norvegicus GN=Canx PE=1 SV=1	0.96	3.08	0.94	0.00	0.00	0.00	0.00	0.00	0.00
Plasma membrane calcium-transporting ATPase 1 OS=Rattus norvegicus GN=Atp2b1 PE=2 SV=2	0.00	3.08	0.94	0.00	4.01	0.92	0.00	1.97	0.00
Myosin-10 OS=Rattus norvegicus GN=Myh10 PE=1 SV=1	0.00	1.03	0.94	0.00	1.00	1.84	0.00	0.99	0.00
Fatty acid-binding protein, brain OS=Rattus norvegicus GN=Fabp7 PE=1 SV=2	0.00	1.03	0.00	0.00	0.00	4.60	0.00	1.97	0.93
Glutathione S-transferase Yb-3 OS=Rattus norvegicus GN=Gstm3 PE=1 SV=2	0.96	0.00	0.00	0.99	1.00	1.84	0.00	1.97	1.86
T-complex protein 1 subunit gamma OS=Rattus norvegicus GN=Cct3 PE=1 SV=1	1.91	1.03	1.88	1.97	0.00	0.00	0.00	1.97	1.86
Mitogen-activated protein kinase 1 OS=Rattus norvegicus GN=Mapk1 PE=1 SV=3	0.96	3.08	0.94	2.96	0.00	1.84	0.00	1.97	0.93
Acetyl-CoA acetyltransferase, mitochondrial OS=Rattus norvegicus GN=Acat1 PE=1 SV=1	1.91	0.00	0.00	2.96	0.00	0.00	1.36	0.99	0.93
Clathrin light chain B OS=Rattus norvegicus GN=Cltb PE=1 SV=1	0.00	1.03	0.00	0.00	1.00	2.76	1.36	0.99	0.00
Guanine nucleotide-binding protein G(i) subunit alpha-2 OS=Rattus norvegicus GN=Gnai2 PE=1 SV=3	0.00	0.00	1.88	0.99	1.00	1.84	1.36	1.97	0.00
Tropomodulin-2 OS=Rattus norvegicus GN=Tmod2 PE=1 SV=1	1.91	1.03	0.94	0.00	0.00	0.92	1.36	0.00	2.80
Thioredoxin-dependent peroxide reductase, mitochondrial OS=Rattus norvegicus GN=Prdx3 PE=1 SV=2	0.96	0.00	0.94	2.96	0.00	0.00	0.00	1.97	0.93
Endophilin-B2 OS=Rattus norvegicus GN=Sh3glb2 PE=2 SV=2	1.91	1.03	0.94	0.00	1.00	3.68	0.00	0.00	0.00
Dynactin subunit 1 OS=Rattus norvegicus GN=Dctn1 PE=2 SV=2	0.96	1.03	1.88	1.97	0.00	2.76	0.00	0.00	1.86
Leucine-rich glioma-inactivated protein 1 OS=Rattus norvegicus GN=Lgi1 PE=1 SV=1	1.91	0.00	2.82	1.97	2.01	0.00	0.00	0.99	1.86
Protein bassoon OS=Rattus norvegicus GN=Bsn PE=1 SV=3	0.00	0.00	1.88	2.96	0.00	1.84	0.00	1.97	0.93
Ras-related protein Rab-11A OS=Rattus norvegicus GN=Rab11a PE=1 SV=3	0.96	0.00	0.94	1.97	0.00	2.76	0.00	0.99	0.00
Keratin, type II cytoskeletal 1 OS=Rattus norvegicus GN=Krt1 PE=2 SV=1	4.78	0.00	0.94	0.99	2.01	1.84	0.00	0.00	0.00
Small glutamine-rich tetratricopeptide repeat-containing protein alpha OS=Rattus norvegicus GN=Sgta PE=1 SV=1	2.87	1.03	0.00	1.97	1.00	1.84	1.36	0.99	0.00
Isocitrate dehydrogenase [NAD] subunit gamma 1, mitochondrial OS=Rattus norvegicus GN=Idh3g PE=2 SV=2	1.91	1.03	0.94	0.99	3.01	0.92	0.00	0.00	1.86
Septin-5 OS=Rattus norvegicus GN=Sept5 PE=1 SV=2	0.96	0.00	0.00	0.00	1.00	3.68	0.00	0.00	0.93

	Quantitative value of spectra counts								
	METH treated Hipp samples					Control			
Identified Proteins (302)	HM2	HM3	HM4	HM5	HM6	HS3	HS4	HS6	HS7
Myelin-oligodendrocyte glycoprotein OS=Rattus norvegicus GN=Mog PE=1 SV=1	0.00	0.00	0.00	2.96	2.01	1.84	2.73	1.97	0.93
Cytochrome b-c1 complex subunit 6, mitochondrial OS=Rattus norvegicus GN=Uqcrh PE=2 SV=1	1.91	0.00	0.00	0.99	0.00	0.92	0.00	0.00	3.73
Cyclin-dependent kinase 5 OS=Rattus norvegicus GN=Cdk5 PE=1 SV=1	0.96	1.03	2.82	0.00	2.01	0.00	0.00	1.97	0.93
Obg-like ATPase 1 OS=Rattus norvegicus GN=Ola1 PE=2 SV=1	0.96	1.03	1.88	0.99	1.00	0.00	0.00	2.96	1.86
AP2-associated protein kinase 1 OS=Rattus norvegicus GN=Aak1 PE=1 SV=1	0.96	0.00	0.94	0.00	1.00	0.92	5.46	0.00	0.93
Acidic leucine-rich nuclear phosphoprotein 32 family member A OS=Rattus norvegicus GN=Anp32a PE=2 SV=1	0.96	1.03	1.88	0.00	1.00	1.84	1.36	0.00	0.00
T-complex protein 1 subunit delta OS=Rattus norvegicus GN=Cct4 PE=1 SV=3	1.91	1.03	0.94	0.99	0.00	1.84	0.00	0.99	0.00
Wiskott-Aldrich syndrome protein family member 1 OS=Rattus norvegicus GN=Wasf1 PE=2 SV=1	1.91	0.00	0.00	2.96	2.01	1.84	1.36	0.00	0.00
Sarcoplasmic/endoplasmic reticulum calcium ATPase 2 OS=Rattus norvegicus GN=Atp2a2 PE=1 SV=1	0.00	0.00	0.00	0.00	2.01	0.00	0.00	1.97	0.93
Prohibitin OS=Rattus norvegicus GN=Phb PE=1 SV=1	0.00	0.00	0.00	1.97	2.01	0.00	2.73	0.00	2.80
Keratin, type II cytoskeletal 6A OS=Rattus norvegicus GN=Krt6a PE=1 SV=1	4.78	2.06	0.00	0.00	1.00	0.92	2.73	0.00	0.00
Actin-related protein 2 OS=Rattus norvegicus GN=Actr2 PE=1 SV=1	1.91	3.08	0.94	0.99	2.01	0.00	0.00	0.00	0.00
Ras GTPase-activating protein SynGAP OS=Rattus norvegicus GN=Syngap1 PE=1 SV=2	0.00	1.03	0.00	0.00	2.01	0.00	0.00	0.00	0.00
F-actin-capping protein subunit beta OS=Rattus norvegicus GN=Capzb PE=1 SV=1	0.00	1.03	0.94	1.97	0.00	0.00	1.36	0.99	1.86
Glutathione S-transferase alpha-3 OS=Rattus norvegicus GN=Gsta3 PE=1 SV=3	0.96	2.06	0.94	1.97	1.00	0.92	0.00	0.00	0.00
Guanine nucleotide-binding protein G(i) subunit alpha-1 OS=Rattus norvegicus GN=Gnai1 PE=1 SV=3	0.00	0.00	0.94	1.97	1.00	2.76	1.36	0.00	0.00
Guanine nucleotide-binding protein G(q) subunit alpha OS=Rattus norvegicus GN=Gnaq PE=1 SV=2	0.00	1.03	0.94	1.97	0.00	0.92	1.36	0.99	0.00
Alpha-actinin-4 OS=Rattus norvegicus GN=Actn4 PE=1 SV=2	3.82	4.11	7.53	1.97	4.01	5.52	0.00	2.96	6.52
Alpha-soluble NSF attachment protein OS=Rattus norvegicus GN=Napa PE=1 SV=2	3.82	2.06	0.94	1.97	0.00	5.52	1.36	0.99	0.93
Thioredoxin-like protein 1 OS=Rattus norvegicus GN=Txnl1 PE=1 SV=3	0.00	0.00	0.94	1.97	0.00	0.00	1.36	0.99	1.86
Stathmin OS=Rattus norvegicus GN=Stmn1 PE=1 SV=2	1.91	2.06	0.94	0.99	0.00	0.00	0.00	0.00	0.00
Hyaluronan and proteoglycan link protein 1 OS=Rattus norvegicus GN=Hapln1 PE=1 SV=2	0.00	0.00	0.00	0.00	0.00	0.00	0.00	0.99	1.86
Voltage-dependent anion-selective channel protein 2 OS=Rattus norvegicus GN=Vdac2 PE=1 SV=2	0.00	0.00	0.00	0.00	3.01	0.00	0.00	0.00	0.93
Annexin A5 OS=Rattus norvegicus GN=Anxa5 PE=1 SV=3	1.91	0.00	0.94	0.00	1.00	0.00	0.00	0.00	0.00
L-asparaginase OS=Rattus norvegicus GN=Asrgl1 PE=1 SV=1	0.00	2.06	0.94	0.00	0.00	0.00	0.00	0.00	0.00
Alpha-2-HS-glycoprotein OS=Rattus norvegicus GN=Ahsg PE=1 SV=2	0.00	0.00	0.00	0.00	0.00	0.00	0.00	1.97	0.00
Keratin, type I cytoskeletal 14 OS=Rattus norvegicus GN=Krt14 PE=2 SV=1	1.91	6.17	0.94	1.97	2.01	0.92	0.00	0.00	0.00
Microtubule-associated protein 4 OS=Rattus norvegicus GN=Map4 PE=1 SV=1	0.00	2.06	0.00	0.00	0.00	0.00	0.00	0.00	0.00

HM: METH treated hippocampal tissue, HS: Saline injected hippocampal tissue.
