# Supplementary material for: The Effect of Chronic Methamphetamine Exposure on the Hippocampal and Olfactory Bulb Neuroproteomes of Rats
Source: PLoS One. 2016 Apr 15;11(4):e0151034. doi: 10.1371/journal.pone.0151034 (PMC4833297; doi:10.1371/journal.pone.0151034)
Supplement: S3 Table — (PDF) [file pone.0151034.s004.pdf]

Table S3. Entire identified proteins list and quantitative spectra counts value of METH treated olfactory bulb tissues and control

	Quantitative Value of Spectra Counts									
	METH treated OB samples					Control				
Identified Proteins (336)	OM4	OM5	OM6	OM7	OM8	OS2	OS3	OS4	OS5	OS6
Spectrin alpha chain, non-erythrocytic 1 OS=Rattus norvegicus GN=Sptan1 PE=1 SV=2	47.55	44.03	54.03	58.07	41.67	45.99	48.96	42.47	36.61	42.59
Tubulin alpha-1B chain OS=Rattus norvegicus GN=Tuba1b PE=1 SV=1	40.89	42.12	20.53	41.48	44.33	37.81	32.64	36.93	36.61	39.47
Tubulin beta-2B chain OS=Rattus norvegicus GN=Tubb2b PE=1 SV=1	47.55	50.73	47.55	53.93	44.33	55.19	57.75	46.16	54.45	40.51
Creatine kinase B-type OS=Rattus norvegicus GN=Ckb PE=1 SV=2	18.07	23.93	28.10	16.59	27.49	29.64	30.13	39.70	28.16	37.39
Hemoglobin subunit alpha-1/2 OS=Rattus norvegicus GN=Hba1 PE=1 SV=3	38.04	36.37	14.05	48.74	38.13	31.68	30.13	43.39	47.88	44.67
Tubulin alpha-1A chain OS=Rattus norvegicus GN=Tuba1a PE=1 SV=1	66.57	66.05	37.82	86.07	69.16	73.58	50.22	78.48	67.59	62.32
Clathrin heavy chain 1 OS=Rattus norvegicus GN=Cltc PE=1 SV=3	33.28	27.76	33.50	18.67	30.15	26.57	26.36	31.39	36.61	41.55
Vimentin OS=Rattus norvegicus GN=Vim PE=1 SV=2	29.48	27.76	30.26	31.11	26.60	29.64	27.62	29.54	30.98	27.01
Microtubule-associated protein 1B OS=Rattus norvegicus GN=Map1b PE=1 SV=2	22.82	26.80	29.18	30.07	28.37	31.68	37.66	32.31	27.22	30.12
Actin, cytoplasmic 1 OS=Rattus norvegicus GN=Actb PE=1 SV=1	22.82	27.76	22.69	28.00	29.26	25.55	23.85	28.62	23.47	21.81
Serum albumin OS=Rattus norvegicus GN=Alb PE=1 SV=2	21.87	22.02	23.77	29.04	30.15	28.62	27.62	25.85	29.10	27.01
Neural cell adhesion molecule 1 OS=Rattus norvegicus GN=Ncam1 PE=1 SV=1	27.58	30.63	25.94	28.00	22.17	23.51	28.87	19.39	24.41	23.89
Hemoglobin subunit beta-1 OS=Rattus norvegicus GN=Hbb PE=1 SV=3	25.68	22.02	19.45	20.74	23.94	22.48	31.38	23.08	24.41	22.85
Dihydropyrimidinase-related protein 2 OS=Rattus norvegicus GN=Dpysl2 PE=1 SV=1	19.97	21.06	20.53	23.85	18.62	13.29	25.11	20.31	17.84	21.81
Heat shock cognate 71 kDa protein OS=Rattus norvegicus GN=Hspa8 PE=1 SV=1	24.73	20.10	31.34	23.85	25.71	24.53	21.34	21.24	20.65	21.81
ATP synthase subunit beta, mitochondrial OS=Rattus norvegicus GN=Atp5b PE=1 SV=2	21.87	25.84	25.94	19.70	20.39	20.44	26.36	18.47	21.59	21.81
Sodium/potassium-transporting ATPase subunit alpha-1 OS=Rattus norvegicus GN=Atp1a1 PE=1 SV=1	23.77	22.02	19.45	19.70	23.94	22.48	17.58	18.47	18.78	18.70
Heat shock protein HSP 90-alpha OS=Rattus norvegicus GN=Hsp90aa1 PE=1 SV=3	17.12	19.14	21.61	16.59	15.96	12.26	21.34	10.16	17.84	18.70
Aconitate hydratase, mitochondrial OS=Rattus norvegicus GN=Aco2 PE=1 SV=2	13.31	19.14	23.77	12.44	16.85	17.37	26.36	17.54	19.71	18.70
Alpha-enolase OS=Rattus norvegicus GN=Eno1 PE=1 SV=4	17.12	14.36	7.56	10.37	19.51	14.31	16.32	15.70	24.41	13.50
Syntaxin-binding protein 1 OS=Rattus norvegicus GN=Stxbp1 PE=1 SV=1	17.12	18.19	23.77	13.48	15.96	14.31	16.32	11.08	19.71	11.43
Pyruvate kinase isozymes M1/M2 OS=Rattus norvegicus GN=Pkm PE=1 SV=3	19.97	22.97	21.61	17.63	21.28	20.44	16.32	17.54	20.65	19.74
Microtubule-associated protein 1A OS=Rattus norvegicus GN=Map1a PE=1 SV=1	5.71	10.53	9.73	17.63	12.41	11.24	21.34	16.62	15.02	10.39
Olfactory marker protein OS=Rattus norvegicus GN=Omp PE=1 SV=2	15.22	12.44	12.97	10.37	10.64	14.31	13.81	12.93	13.14	14.54
Microtubule-associated protein 2 OS=Rattus norvegicus GN=Map2 PE=1 SV=3	12.36	11.49	8.65	19.70	14.19	12.26	8.79	12.93	10.33	16.62
Tubulin beta-3 chain OS=Rattus norvegicus GN=Tubb3 PE=1 SV=1	30.43	34.46	33.50	37.33	31.92	38.84	42.68	27.70	39.43	31.16
Guanine nucleotide-binding protein G(o) subunit alpha OS=Rattus norvegicus GN=Gnao1 PE=1 SV=2	11.41	9.57	12.97	9.33	11.53	13.29	12.55	12.93	15.02	9.35
ATP synthase subunit alpha, mitochondrial OS=Rattus norvegicus GN=Atp5a1 PE=1 SV=2	12.36	12.44	16.21	10.37	13.30	12.26	12.55	12.93	11.27	12.47
Sodium/potassium-transporting ATPase subunit alpha-3 OS=Rattus norvegicus GN=Atp1a3 PE=1 SV=2	19.97	21.06	25.94	23.85	29.26	23.51	15.07	24.01	20.65	18.70
Calmodulin OS=Rattus norvegicus GN=Calm1 PE=1 SV=2	12.36	11.49	14.05	12.44	14.19	13.29	12.55	11.08	11.27	10.39
Fructose-bisphosphate aldolase A OS=Rattus norvegicus GN=Aldoa PE=1 SV=2	10.46	11.49	14.05	10.37	11.53	9.20	12.55	12.93	11.27	9.35
14-3-3 protein zeta/delta OS=Rattus norvegicus GN=Ywhaz PE=1 SV=1	16.17	11.49	16.21	9.33	15.96	17.37	20.09	17.54	10.33	16.62
14-3-3 protein epsilon OS=Rattus norvegicus GN=Ywhae PE=1 SV=1	10.46	12.44	12.97	8.30	9.75	10.22	18.83	12.00	7.51	9.35
Malate dehydrogenase, cytoplasmic OS=Rattus norvegicus GN=Mdh1 PE=1 SV=3	14.26	8.61	12.97	9.33	7.09	11.24	7.53	11.08	11.27	7.27
Ras-related protein Rab-3A OS=Rattus norvegicus GN=Rab3a PE=1 SV=1	8.56	11.49	10.81	8.30	10.64	9.20	12.55	6.46	11.27	7.27
Rab GDP dissociation inhibitor alpha OS=Rattus norvegicus GN=Gdi1 PE=1 SV=1	8.56	8.61	12.97	12.44	10.64	10.22	12.55	8.31	8.45	12.47
Heat shock protein HSP 90-beta OS=Rattus norvegicus GN=Hsp90ab1 PE=1 SV=4	10.46	11.49	14.05	10.37	9.75	10.22	13.81	8.31	9.39	14.54
Glucose-6-phosphate isomerase OS=Rattus norvegicus GN=Gpi PE=1 SV=1	7.61	11.49	9.73	9.33	7.98	6.13	8.79	10.16	7.51	9.35
Hexokinase-1 OS=Rattus norvegicus GN=Hk1 PE=1 SV=4	10.46	9.57	8.65	10.37	10.64	9.20	8.79	9.23	7.51	13.50
Tubulin beta-4B chain OS=Rattus norvegicus GN=Tubb4b PE=1 SV=1	45.65	48.82	47.55	60.15	45.22	49.06	52.73	42.47	57.26	41.55
Triosephosphate isomerase OS=Rattus norvegicus GN=Tpi1 PE=1 SV=2	10.46	9.57	10.81	10.37	8.87	7.15	16.32	10.16	8.45	7.27
Glutamate dehydrogenase 1, mitochondrial OS=Rattus norvegicus GN=Glud1 PE=1 SV=2	9.51	6.70	6.48	7.26	8.87	6.13	10.04	10.16	8.45	7.27
Glial fibrillary acidic protein OS=Rattus norvegicus GN=Gfap PE=1 SV=2	11.41	9.57	7.56	15.56	12.41	6.13	5.02	9.23	12.20	8.31
Aspartate aminotransferase, cytoplasmic OS=Rattus norvegicus GN=Got1 PE=1 SV=3	6.66	5.74	5.40	10.37	7.09	8.18	13.81	8.31	12.20	8.31
60 kDa heat shock protein, mitochondrial OS=Rattus norvegicus GN=Hspd1 PE=1 SV=1	6.66	6.70	11.89	8.30	6.21	7.15	7.53	12.93	9.39	10.39

	Quantitative Value of Spectra Counts									
	METH treated OB samples					Control				
Identified Proteins (336)	OM4	OM5	OM6	OM7	OM8	OS2	OS3	OS4	OS5	OS6
Elongation factor 1-alpha 1 OS=Rattus norvegicus GN=Eef1a1 PE=1 SV=1	11.41	11.49	5.40	5.19	10.64	8.18	6.28	11.08	7.51	8.31
Vesicle-fusing ATPase OS=Rattus norvegicus GN=Nsf PE=1 SV=1	10.46	9.57	12.97	8.30	8.87	7.15	11.30	9.23	12.20	4.15
Guanine nucleotide-binding protein G(I)/G(S)/G(T) subunit beta-1 OS=Rattus norvegicus GN=Gnb1 PE=1 SV=4	6.66	11.49	12.97	10.37	6.21	11.24	5.02	10.16	9.39	9.35
Calretinin OS=Rattus norvegicus GN=Calb2 PE=1 SV=1	11.41	9.57	15.13	9.33	5.32	6.13	11.30	9.23	10.33	10.39
Dynamin-1 OS=Rattus norvegicus GN=Dnm1 PE=1 SV=2	7.61	9.57	9.73	9.33	7.98	9.20	8.79	9.23	7.51	7.27
Malate dehydrogenase, mitochondrial OS=Rattus norvegicus GN=Mdh2 PE=1 SV=2	8.56	7.66	9.73	3.11	8.87	8.18	8.79	7.39	9.39	11.43
Serotransferrin OS=Rattus norvegicus GN=Tf PE=1 SV=3	7.61	9.57	5.40	6.22	5.32	6.13	7.53	9.23	10.33	12.47
Glyceraldehyde-3-phosphate dehydrogenase OS=Rattus norvegicus GN=Gapdh PE=1 SV=3	6.66	8.61	7.56	7.26	8.87	8.18	8.79	4.62	4.69	8.31
Aspartate aminotransferase, mitochondrial OS=Rattus norvegicus GN=Got2 PE=1 SV=2	12.36	6.70	8.65	6.22	7.98	11.24	12.55	6.46	9.39	9.35
2',3'-cyclic-nucleotide 3'-phosphodiesterase OS=Rattus norvegicus GN=Cnp PE=1 SV=2	4.75	4.79	8.65	9.33	4.43	7.15	6.28	9.23	6.57	6.23
Ubiquitin-like modifier-activating enzyme 1 OS=Rattus norvegicus GN=Uba1 PE=1 SV=1	10.46	5.74	6.48	7.26	4.43	6.13	7.53	5.54	12.20	7.27
Glutamine synthetase OS=Rattus norvegicus GN=Glul PE=1 SV=3	9.51	8.61	8.65	8.30	3.55	8.18	10.04	6.46	9.39	8.31
Pyruvate dehydrogenase E1 component subunit beta, mitochondrial OS=Rattus norvegicus GN=Pdhb PE=1 SV=2	4.75	5.74	6.48	6.22	5.32	3.07	5.02	6.46	3.76	5.19
Syntaxin-1B OS=Rattus norvegicus GN=Stx1b PE=1 SV=1	9.51	7.66	6.48	5.19	8.87	5.11	5.02	11.08	5.63	6.23
Dihydropyrimidinase-related protein 3 OS=Rattus norvegicus GN=Dpysl3 PE=1 SV=2	9.51	10.53	11.89	11.41	10.64	9.20	13.81	9.23	8.45	13.50
Cytochrome b-c1 complex subunit 1, mitochondrial OS=Rattus norvegicus GN=Uqcrc1 PE=1 SV=1	5.71	6.70	6.48	4.15	6.21	7.15	7.53	7.39	9.39	7.27
Sodium/potassium-transporting ATPase subunit beta-1 OS=Rattus norvegicus GN=Atp1b1 PE=1 SV=1	6.66	7.66	7.56	5.19	7.09	7.15	10.04	6.46	6.57	7.27
Ubiquitin carboxyl-terminal hydrolase isozyme L1 OS=Rattus norvegicus GN=Uchl1 PE=1 SV=2	7.61	7.66	6.48	9.33	8.87	7.15	10.04	7.39	6.57	7.27
Gamma-enolase OS=Rattus norvegicus GN=Eno2 PE=1 SV=2	12.36	15.32	8.65	5.19	17.73	13.29	15.07	14.77	21.59	15.58
Glucose-6-phosphate 1-dehydrogenase OS=Rattus norvegicus GN=G6pdx PE=1 SV=3	5.71	6.70	7.56	7.26	6.21	7.15	5.02	5.54	5.63	7.27
Annexin A5 OS=Rattus norvegicus GN=Anxa5 PE=1 SV=3	10.46	7.66	8.65	3.11	6.21	10.22	8.79	7.39	4.69	9.35
Cytoplasmic dynein 1 heavy chain 1 OS=Rattus norvegicus GN=Dync1h1 PE=1 SV=1	3.80	7.66	4.32	2.07	0.89	6.13	5.02	5.54	1.88	1.04
Microtubule-associated protein 6 OS=Rattus norvegicus GN=Map6 PE=1 SV=1	3.80	4.79	5.40	8.30	3.55	6.13	7.53	8.31	9.39	5.19
Brain acid soluble protein 1 OS=Rattus norvegicus GN=Basp1 PE=1 SV=2	3.80	3.83	4.32	8.30	6.21	9.20	5.02	6.46	3.76	8.31
Transitional endoplasmic reticulum ATPase OS=Rattus norvegicus GN=Vcp PE=1 SV=3	5.71	6.70	7.56	10.37	5.32	5.11	5.02	4.62	2.82	8.31
Fructose-bisphosphate aldolase C OS=Rattus norvegicus GN=Aldoc PE=1 SV=3	9.51	8.61	7.56	7.26	7.98	6.13	2.51	2.77	4.69	4.15
Cofilin-1 OS=Rattus norvegicus GN=Cfl1 PE=1 SV=3	4.75	5.74	5.40	5.19	8.87	6.13	6.28	6.46	4.69	7.27
Myelin proteolipid protein OS=Rattus norvegicus GN=Plp1 PE=1 SV=2	6.66	6.70	4.32	7.26	8.87	7.15	3.77	8.31	5.63	7.27
Histone H4 OS=Rattus norvegicus GN=Hist1h4b PE=1 SV=2	5.71	3.83	5.40	4.15	5.32	8.18	7.53	6.46	8.45	8.31
Neuromodulin OS=Rattus norvegicus GN=Gap43 PE=1 SV=1	5.71	5.74	6.48	5.19	7.09	5.11	7.53	5.54	6.57	4.15
Adenylate kinase isoenzyme 1 OS=Rattus norvegicus GN=Ak1 PE=1 SV=3	8.56	3.83	6.48	8.30	6.21	5.11	2.51	6.46	6.57	7.27
Heterogeneous nuclear ribonucleoprotein K OS=Rattus norvegicus GN=Hnrnpk PE=1 SV=1	6.66	6.70	7.56	4.15	5.32	11.24	6.28	6.46	6.57	4.15
L-lactate dehydrogenase B chain OS=Rattus norvegicus GN=Ldhb PE=1 SV=2	3.80	4.79	5.40	4.15	10.64	3.07	3.77	3.69	7.51	4.15
Voltage-dependent anion-selective channel protein 1 OS=Rattus norvegicus GN=Vdac1 PE=1 SV=4	3.80	3.83	6.48	3.11	7.98	9.20	5.02	8.31	6.57	6.23
Glycogen phosphorylase, brain form (Fragment) OS=Rattus norvegicus GN=Pygb PE=1 SV=3	2.85	2.87	3.24	3.11	5.32	6.13	5.02	2.77	3.76	7.27
Alpha-actinin-1 OS=Rattus norvegicus GN=Actn1 PE=1 SV=1	5.71	7.66	4.32	6.22	2.66	2.04	8.79	5.54	3.76	4.15
Peptidyl-prolyl cis-trans isomerase A OS=Rattus norvegicus GN=Ppia PE=1 SV=2	4.75	8.61	4.32	7.26	2.66	5.11	2.51	3.69	6.57	7.27
14-3-3 protein theta OS=Rattus norvegicus GN=Ywhaq PE=1 SV=1	12.36	10.53	12.97	9.33	11.53	11.24	8.79	9.23	7.51	10.39
Phosphoglycerate kinase 1 OS=Rattus norvegicus GN=Pgk1 PE=1 SV=2	7.61	2.87	6.48	6.22	6.21	9.20	8.79	10.16	5.63	5.19
Alpha-1-inhibitor 3 OS=Rattus norvegicus GN=A1i3 PE=1 SV=1	3.80	0.96	1.08	6.22	6.21	2.04	2.51	2.77	3.76	2.08
Cytochrome b-c1 complex subunit 2, mitochondrial OS=Rattus norvegicus GN=Uqcrc2 PE=1 SV=2	9.51	6.70	5.40	5.19	5.32	5.11	5.02	5.54	3.76	4.15
Citrate synthase, mitochondrial OS=Rattus norvegicus GN=Cs PE=1 SV=1	3.80	5.74	6.48	4.15	7.09	10.22	7.53	6.46	5.63	5.19
14-3-3 protein beta/alpha OS=Rattus norvegicus GN=Ywhab PE=1 SV=3	9.51	10.53	6.48	4.15	7.09	7.15	6.28	7.39	6.57	10.39
Succinate dehydrogenase [ubiquinone] flavoprotein subunit, mitochondrial OS=Rattus norvegicus GN=Sdha PE=1 SV=1	4.75	5.74	5.40	4.15	6.21	6.13	3.77	8.31	3.76	8.31
Myristoylated alanine-rich C-kinase substrate OS=Rattus norvegicus GN=Marcks PE=1 SV=2	5.71	3.83	8.65	6.22	3.55	6.13	5.02	4.62	4.69	5.19
14-3-3 protein gamma OS=Rattus norvegicus GN=Ywhag PE=1 SV=2	11.41	9.57	10.81	8.30	10.64	12.26	10.04	9.23	8.45	5.19

	Quantitative Value of Spectra Counts									
	METH treated OB samples					Control				
Identified Proteins (336)	OM4	OM5	OM6	OM7	OM8	OS2	OS3	OS4	OS5	OS6
NADH-ubiquinone oxidoreductase 75 kDa subunit, mitochondrial OS=Rattus norvegicus GN=Ndufs1 PE=1 SV=1	6.66	3.83	3.24	7.26	5.32	6.13	5.02	6.46	7.51	7.27
Dihydropyrimidinase-related protein 1 OS=Rattus norvegicus GN=Crmp1 PE=1 SV=1	8.56	9.57	9.73	17.63	10.64	9.20	7.53	6.46	8.45	12.47
Fatty acid-binding protein, brain OS=Rattus norvegicus GN=Fabp7 PE=1 SV=2	6.66	4.79	4.32	4.15	3.55	6.13	8.79	3.69	5.63	7.27
Calreticulin OS=Rattus norvegicus GN=Calr PE=1 SV=1	2.85	1.91	2.16	5.19	2.66	7.15	2.51	3.69	1.88	5.19
Fascin OS=Rattus norvegicus GN=Fscn1 PE=1 SV=2	4.75	5.74	7.56	5.19	3.55	4.09	6.28	3.69	3.76	6.23
Cytochrome c, somatic OS=Rattus norvegicus GN=Cycs PE=1 SV=2	5.71	8.61	4.32	4.15	5.32	6.13	6.28	5.54	5.63	5.19
ATP synthase-coupling factor 6, mitochondrial OS=Rattus norvegicus GN=Atp5j PE=1 SV=1	5.71	7.66	6.48	5.19	7.98	4.09	7.53	5.54	1.88	5.19
78 kDa glucose-regulated protein OS=Rattus norvegicus GN=Hspa5 PE=1 SV=1	11.41	10.53	10.81	6.22	8.87	8.18	8.79	10.16	7.51	6.23
Peroxiredoxin-6 OS=Rattus norvegicus GN=Prdx6 PE=1 SV=3	4.75	4.79	7.56	4.15	6.21	1.02	1.26	3.69	2.82	4.15
Superoxide dismutase [Cu-Zn] OS=Rattus norvegicus GN=Sod1 PE=1 SV=2	5.71	4.79	5.40	2.07	5.32	0.00	5.02	3.69	4.69	6.23
AP-2 complex subunit beta OS=Rattus norvegicus GN=Ap2b1 PE=1 SV=1	4.75	4.79	5.40	3.11	3.55	2.04	2.51	3.69	3.76	6.23
Peroxiredoxin-1 OS=Rattus norvegicus GN=Prdx1 PE=1 SV=1	2.85	4.79	8.65	1.04	5.32	4.09	6.28	3.69	5.63	7.27
Tubulin alpha-4A chain OS=Rattus norvegicus GN=Tuba4a PE=2 SV=1	37.09	41.16	19.45	35.26	39.01	35.77	27.62	28.62	29.10	35.32
Cullin-associated NEDD8-dissociated protein 1 OS=Rattus norvegicus GN=Cand1 PE=1 SV=1	1.90	1.91	0.00	6.22	5.32	3.07	1.26	4.62	2.82	3.12
Excitatory amino acid transporter 2 OS=Rattus norvegicus GN=Slc1a2 PE=1 SV=2	4.75	4.79	7.56	6.22	5.32	4.09	6.28	5.54	5.63	5.19
Histone H2B type 1 OS=Rattus norvegicus PE=1 SV=2	4.75	5.74	3.24	3.11	3.55	3.07	5.02	0.92	1.88	6.23
Aldehyde dehydrogenase, mitochondrial OS=Rattus norvegicus GN=Aldh2 PE=1 SV=1	5.71	3.83	6.48	3.11	3.55	5.11	3.77	6.46	3.76	4.15
Rab GDP dissociation inhibitor beta OS=Rattus norvegicus GN=Gdi2 PE=1 SV=2	5.71	2.87	8.65	2.07	5.32	8.18	6.28	4.62	8.45	7.27
Ubiquitin-60S ribosomal protein L40 OS=Rattus norvegicus GN=Uba52 PE=1 SV=2	1.90	2.87	3.24	1.04	2.66	3.07	5.02	2.77	3.76	4.15
Synapsin-2 OS=Rattus norvegicus GN=Syn2 PE=1 SV=1	3.80	2.87	2.16	5.19	2.66	5.11	3.77	4.62	2.82	2.08
Tubulin beta-5 chain OS=Rattus norvegicus GN=Tubb5 PE=1 SV=1	47.55	49.77	42.14	61.18	46.11	55.19	46.45	43.39	57.26	42.59
Beta-soluble NSF attachment protein OS=Rattus norvegicus GN=Napb PE=1 SV=1	3.80	5.74	1.08	4.15	2.66	2.04	0.00	3.69	4.69	6.23
Protein disulfide-isomerase A3 OS=Rattus norvegicus GN=Pdia3 PE=1 SV=2	1.90	3.83	6.48	5.19	3.55	5.11	2.51	5.54	5.63	4.15
Synapsin-1 OS=Rattus norvegicus GN=Syn1 PE=1 SV=3	4.75	5.74	2.16	2.07	5.32	3.07	2.51	2.77	0.00	6.23
Calcium/calmodulin-dependent protein kinase type II subunit alpha OS=Rattus norvegicus GN=Camk2a PE=1 SV=1	2.85	2.87	3.24	8.30	4.43	3.07	2.51	5.54	3.76	1.04
Phosphatidylethanolamine-binding protein 1 OS=Rattus norvegicus GN=Pebp1 PE=1 SV=3	4.75	4.79	4.32	3.11	5.32	5.11	5.02	6.46	4.69	5.19
Secernin-1 OS=Rattus norvegicus GN=Scrn1 PE=1 SV=1	3.80	4.79	5.40	6.22	5.32	4.09	3.77	4.62	3.76	4.15
AP-2 complex subunit alpha-2 OS=Rattus norvegicus GN=Ap2a2 PE=1 SV=3	5.71	5.74	3.24	1.04	4.43	4.09	2.51	1.85	2.82	5.19
Peroxiredoxin-2 OS=Rattus norvegicus GN=Prdx2 PE=1 SV=3	2.85	1.91	2.16	2.07	4.43	6.13	1.26	2.77	5.63	6.23
2-oxoglutarate dehydrogenase, mitochondrial OS=Rattus norvegicus GN=Ogdh PE=1 SV=1	3.80	1.91	4.32	1.04	1.77	3.07	2.51	2.77	3.76	4.15
Dihydrolipoyl dehydrogenase, mitochondrial OS=Rattus norvegicus GN=Dld PE=1 SV=1	2.85	3.83	6.48	4.15	1.77	1.02	5.02	3.69	2.82	2.08
Clathrin coat assembly protein AP180 OS=Rattus norvegicus GN=Snap91 PE=1 SV=1	2.85	5.74	4.32	2.07	6.21	2.04	5.02	6.46	1.88	3.12
14-3-3 protein eta OS=Rattus norvegicus GN=Ywhah PE=1 SV=2	6.66	9.57	7.56	7.26	6.21	9.20	7.53	9.23	6.57	7.27
Peroxiredoxin-5, mitochondrial OS=Rattus norvegicus GN=Prdx5 PE=1 SV=1	4.75	2.87	8.65	2.07	3.55	4.09	6.28	3.69	4.69	2.08
V-type proton ATPase subunit C 1 OS=Rattus norvegicus GN=Atp6v1c1 PE=2 SV=1	2.85	2.87	3.24	3.11	2.66	4.09	5.02	0.92	4.69	6.23
Amphiphysin OS=Rattus norvegicus GN=Amph PE=1 SV=1	0.95	3.83	2.16	6.22	2.66	2.04	8.79	1.85	5.63	1.04
L-lactate dehydrogenase A chain OS=Rattus norvegicus GN=Ldha PE=1 SV=1	1.90	3.83	8.65	3.11	3.55	4.09	0.00	0.92	0.94	3.12
Isocitrate dehydrogenase [NADP], mitochondrial OS=Rattus norvegicus GN=Idh2 PE=1 SV=2	3.80	3.83	5.40	3.11	1.77	4.09	0.00	2.77	4.69	2.08
Ubiquitin thioesterase OTUB1 OS=Rattus norvegicus GN=Otub1 PE=1 SV=1	2.85	3.83	5.40	2.07	2.66	1.02	5.02	3.69	3.76	4.15
Transketolase OS=Rattus norvegicus GN=Tkt PE=1 SV=1	4.75	2.87	4.32	1.04	1.77	3.07	5.02	5.54	0.94	2.08
10 kDa heat shock protein, mitochondrial OS=Rattus norvegicus GN=Hspe1 PE=1 SV=3	0.95	3.83	4.32	2.07	0.89	1.02	3.77	0.92	1.88	2.08
Coactosin-like protein OS=Rattus norvegicus GN=Cotl1 PE=1 SV=1	2.85	1.91	3.24	3.11	0.89	4.09	0.00	4.62	2.82	1.04
Sulfated glycoprotein 1 OS=Rattus norvegicus GN=Psap PE=1 SV=1	2.85	3.83	1.08	3.11	2.66	3.07	2.51	2.77	2.82	2.08
NAD-dependent protein deacetylase sirtuin-2 OS=Rattus norvegicus GN=Sirt2 PE=1 SV=1	4.75	5.74	2.16	3.11	2.66	3.07	2.51	1.85	3.76	5.19
Synaptotagmin-1 OS=Rattus norvegicus GN=Syt1 PE=1 SV=3	2.85	2.87	4.32	4.15	4.43	3.07	6.28	1.85	2.82	3.12
Septin-11 OS=Rattus norvegicus GN=Sept11 PE=1 SV=1	3.80	0.96	2.16	2.07	3.55	2.04	2.51	2.77	3.76	4.15

	Quantitative Value of Spectra Counts									
	METH treated OB samples					Control				
Identified Proteins (336)	OM4	OM5	OM6	OM7	OM8	OS2	OS3	OS4	OS5	OS6
Dynactin subunit 2 OS=Rattus norvegicus GN=Dctn2 PE=1 SV=1	1.90	0.96	2.16	3.11	3.55	2.04	2.51	1.85	1.88	2.08
Heterogeneous nuclear ribonucleoprotein A3 OS=Rattus norvegicus GN=Hnrnpa3 PE=1 SV=1	2.85	2.87	3.24	4.15	5.32	5.11	3.77	3.69	1.88	5.19
Reticulon-1 OS=Rattus norvegicus GN=Rtn1 PE=2 SV=1	1.90	2.87	3.24	4.15	3.55	4.09	2.51	1.85	2.82	4.15
Growth factor receptor-bound protein 2 OS=Rattus norvegicus GN=Grb2 PE=1 SV=1	2.85	4.79	6.48	3.11	4.43	3.07	2.51	2.77	0.94	3.12
4-aminobutyrate aminotransferase, mitochondrial OS=Rattus norvegicus GN=Abat PE=1 SV=3	0.95	1.91	2.16	2.07	2.66	3.07	0.00	3.69	2.82	1.04
Succinate-semialdehyde dehydrogenase, mitochondrial OS=Rattus norvegicus GN=Aldh5a1 PE=1 SV=2	1.90	1.91	4.32	3.11	2.66	3.07	2.51	3.69	3.76	2.08
Isocitrate dehydrogenase [NAD] subunit beta, mitochondrial OS=Rattus norvegicus GN=Idh3B PE=1 SV=1	2.85	1.91	3.24	3.11	1.77	2.04	1.26	2.77	2.82	2.08
Serine/threonine-protein phosphatase 2B catalytic subunit alpha isoform OS=Rattus norvegicus GN=Ppp3ca PE=1 SV=1	3.80	1.91	3.24	3.11	3.55	5.11	5.02	3.69	4.69	3.12
Astrocytic phosphoprotein PEA-15 OS=Rattus norvegicus GN=Pea15 PE=1 SV=1	3.80	3.83	1.08	4.15	1.77	0.00	5.02	2.77	2.82	2.08
Carbonic anhydrase 2 OS=Rattus norvegicus GN=Ca2 PE=1 SV=2	1.90	3.83	0.00	2.07	3.55	3.07	3.77	3.69	0.94	2.08
ADP-ribosylation factor 1 OS=Rattus norvegicus GN=Arf1 PE=1 SV=2	4.75	3.83	7.56	3.11	4.43	2.04	5.02	1.85	2.82	3.12
Phosphoglycerate mutase 1 OS=Rattus norvegicus GN=Pgam1 PE=1 SV=4	2.85	1.91	3.24	3.11	1.77	3.07	6.28	0.92	3.76	2.08
V-type proton ATPase subunit E 1 OS=Rattus norvegicus GN=Atp6v1e1 PE=1 SV=1	2.85	2.87	3.24	4.15	2.66	3.07	2.51	2.77	2.82	3.12
Serine/threonine-protein phosphatase PP1-alpha catalytic subunit OS=Rattus norvegicus GN=Ppp1ca PE=1 SV=1	0.95	4.79	4.32	2.07	2.66	1.02	3.77	3.69	2.82	7.27
Creatine kinase U-type, mitochondrial OS=Rattus norvegicus GN=Ckmt1 PE=1 SV=1	2.85	1.91	5.40	3.11	3.55	4.09	2.51	4.62	2.82	1.04
Synaptosomal-associated protein 25 OS=Rattus norvegicus GN=Snap25 PE=1 SV=1	3.80	1.91	2.16	2.07	5.32	2.04	1.26	5.54	3.76	4.15
Stress-induced-phosphoprotein 1 OS=Rattus norvegicus GN=Stip1 PE=1 SV=1	3.80	0.96	5.40	1.04	0.89	3.07	2.51	0.92	0.94	2.08
Guanine deaminase OS=Rattus norvegicus GN=Gda PE=1 SV=1	3.80	1.91	5.40	2.07	3.55	4.09	1.26	2.77	3.76	1.04
Complement component 1 Q subcomponent-binding protein, mitochondrial OS=Rattus norvegicus GN=C1qbp PE=1 SV=2	2.85	1.91	3.24	1.04	1.77	3.07	2.51	1.85	2.82	4.15
Histone H2A.Z OS=Rattus norvegicus GN=H2afz PE=1 SV=2	3.80	2.87	2.16	4.15	3.55	4.09	5.02	2.77	2.82	3.12
Neuronal membrane glycoprotein M6-a OS=Rattus norvegicus GN=Gpm6a PE=1 SV=1	1.90	2.87	0.00	3.11	0.00	2.04	1.26	2.77	1.88	1.04
6-phosphofructokinase, muscle type OS=Rattus norvegicus GN=Pfkm PE=2 SV=3	3.80	0.00	4.32	4.15	2.66	3.07	0.00	0.92	2.82	2.08
Secretagogin OS=Rattus norvegicus GN=Scgn PE=1 SV=1	2.85	2.87	2.16	3.11	2.66	2.04	1.26	0.92	2.82	3.12
Histidine triad nucleotide-binding protein 1 OS=Rattus norvegicus GN=Hint1 PE=1 SV=5	2.85	2.87	1.08	0.00	0.89	2.04	1.26	3.69	1.88	2.08
GTP-binding nuclear protein Ran, testis-specific isoform OS=Rattus norvegicus GN=Rasl2-9 PE=2 SV=1	4.75	4.79	3.24	0.00	3.55	2.04	5.02	4.62	0.94	3.12
Alpha-synuclein OS=Rattus norvegicus GN=Snca PE=1 SV=1	3.80	0.96	3.24	3.11	3.55	2.04	0.00	2.77	2.82	1.04
Protein DJ-1 OS=Rattus norvegicus GN=Park7 PE=1 SV=1	2.85	4.79	3.24	3.11	2.66	1.02	0.00	1.85	2.82	2.08
Long-chain-fatty-acid--CoA ligase ACSBG1 OS=Rattus norvegicus GN=Acsbg1 PE=1 SV=1	1.90	0.96	4.32	0.00	5.32	3.07	3.77	6.46	3.76	3.12
Elongation factor 2 OS=Rattus norvegicus GN=Eef2 PE=1 SV=4	1.90	1.91	2.16	4.15	2.66	2.04	2.51	2.77	2.82	3.12
Dynamin-1-like protein OS=Rattus norvegicus GN=Dnm1l PE=1 SV=1	0.00	3.83	1.08	3.11	4.43	3.07	5.02	2.77	1.88	6.23
Ras-related protein Rap-1A OS=Rattus norvegicus GN=Rap1a PE=1 SV=1	3.80	3.83	2.16	4.15	2.66	3.07	0.00	2.77	2.82	3.12
Tropomyosin alpha-3 chain OS=Rattus norvegicus GN=Tpm3 PE=1 SV=2	1.90	4.79	2.16	3.11	4.43	4.09	5.02	2.77	1.88	3.12
ATP synthase subunit gamma, mitochondrial OS=Rattus norvegicus GN=Atp5c1 PE=1 SV=2	2.85	0.96	3.24	1.04	0.00	1.02	2.51	0.92	0.94	2.08
V-type proton ATPase subunit B, brain isoform OS=Rattus norvegicus GN=Atp6v1b2 PE=1 SV=1	3.80	0.00	4.32	4.15	0.00	1.02	2.51	1.85	0.94	3.12
Heat shock 70 kDa protein 4 OS=Rattus norvegicus GN=Hspa4 PE=1 SV=1	1.90	2.87	3.24	2.07	1.77	2.04	3.77	3.69	2.82	4.15
WD repeat-containing protein 1 OS=Rattus norvegicus GN=Wdr1 PE=1 SV=3	2.85	1.91	3.24	4.15	0.89	2.04	3.77	2.77	0.94	2.08
Apolipoprotein E OS=Rattus norvegicus GN=Apoe PE=1 SV=2	2.85	2.87	4.32	3.11	4.43	5.11	2.51	2.77	0.00	4.15
Alcohol dehydrogenase class-3 OS=Rattus norvegicus GN=Adh5 PE=1 SV=2	2.85	0.96	4.32	2.07	1.77	2.04	1.26	1.85	0.94	4.15
Guanine nucleotide-binding protein G(olf) subunit alpha OS=Rattus norvegicus GN=Gnal PE=2 SV=2	3.80	2.87	4.32	3.11	5.32	2.04	3.77	2.77	1.88	3.12
Clathrin light chain A OS=Rattus norvegicus GN=Clta PE=1 SV=1	0.00	0.96	1.08	2.07	1.77	1.02	1.26	0.92	2.82	3.12
Annexin A6 OS=Rattus norvegicus GN=Anxa6 PE=1 SV=2	2.85	2.87	3.24	2.07	0.00	3.07	1.26	1.85	0.94	4.15
Visinin-like protein 1 OS=Rattus norvegicus GN=Vsnl1 PE=1 SV=2	3.80	3.83	1.08	2.07	2.66	1.02	1.26	1.85	2.82	1.04
Alpha-actinin-4 OS=Rattus norvegicus GN=Actn4 PE=1 SV=2	7.61	9.57	6.48	9.33	4.43	6.13	8.79	7.39	6.57	5.19
Alpha-internexin OS=Rattus norvegicus GN=Ina PE=1 SV=2	3.80	3.83	2.16	3.11	3.55	3.07	2.51	3.69	1.88	5.19
Rho GDP-dissociation inhibitor 1 OS=Rattus norvegicus GN=Arhgdia PE=1 SV=1	0.95	1.91	4.32	1.04	0.89	1.02	1.26	2.77	0.94	2.08
Protein kinase C and casein kinase substrate in neurons protein 1 OS=Rattus norvegicus GN=Pacsin1 PE=1 SV=1	0.95	1.91	1.08	3.11	2.66	3.07	2.51	0.92	2.82	3.12

	Quantitative Value of Spectra Counts									
	METH treated OB samples					Control				
Identified Proteins (336)	OM4	OM5	OM6	OM7	OM8	OS2	OS3	OS4	OS5	OS6
Sodium/potassium-transporting ATPase subunit alpha-2 OS=Rattus norvegicus GN=Atp1a2 PE=1 SV=1	13.31	16.27	16.21	16.59	19.51	16.35	16.32	17.54	14.08	11.43
Neurochondrin OS=Rattus norvegicus GN=Ncdn PE=1 SV=2	0.95	0.00	2.16	0.00	1.77	1.02	0.00	0.00	0.00	1.04
Serine/threonine-protein phosphatase 2A 55 kDa regulatory subunit B alpha isoform OS=Rattus norvegicus GN=Ppp2r2a PE=2 SV=1	1.90	3.83	2.16	3.11	1.77	1.02	0.00	1.85	2.82	3.12
Septin-7 OS=Rattus norvegicus GN=Sept7 PE=1 SV=1	5.71	0.96	0.00	4.15	2.66	4.09	5.02	4.62	2.82	6.23
Mitogen-activated protein kinase 1 OS=Rattus norvegicus GN=Mapk1 PE=1 SV=3	0.95	1.91	2.16	3.11	4.43	1.02	1.26	1.85	1.88	0.00
Prohibitin OS=Rattus norvegicus GN=Phb PE=1 SV=1	1.90	0.96	1.08	2.07	0.00	4.09	2.51	1.85	2.82	2.08
Spectrin beta chain, non-erythrocytic 2 OS=Rattus norvegicus GN=Sptbn2 PE=1 SV=2	3.80	0.96	1.08	1.04	0.00	0.00	0.00	0.00	1.88	2.08
Toll-interacting protein OS=Rattus norvegicus GN=Tollip PE=2 SV=1	1.90	1.91	3.24	4.15	2.66	1.02	2.51	2.77	1.88	2.08
60S acidic ribosomal protein P2 OS=Rattus norvegicus GN=Rplp2 PE=1 SV=2	2.85	1.91	2.16	3.11	1.77	2.04	1.26	3.69	2.82	3.12
NSFL1 cofactor p47 OS=Rattus norvegicus GN=Nsfl1c PE=1 SV=1	1.90	1.91	1.08	2.07	2.66	2.04	0.00	4.62	1.88	3.12
Hemopexin OS=Rattus norvegicus GN=Hpx PE=1 SV=3	0.95	0.96	1.08	1.04	0.00	0.00	0.00	0.00	2.82	1.04
ADP/ATP translocase 2 OS=Rattus norvegicus GN=Slc25a5 PE=1 SV=3	1.90	1.91	2.16	0.00	5.32	4.09	0.00	3.69	3.76	1.04
Calcium-dependent secretion activator 1 OS=Rattus norvegicus GN=Cadps PE=1 SV=1	3.80	0.96	0.00	0.00	2.66	0.00	0.00	1.85	0.00	0.00
Plasma membrane calcium-transporting ATPase 4 OS=Rattus norvegicus GN=Atp2b4 PE=2 SV=1	1.90	0.96	2.16	2.07	3.55	2.04	2.51	2.77	0.94	2.08
Stress-70 protein, mitochondrial OS=Rattus norvegicus GN=Hspa9 PE=1 SV=3	2.85	3.83	2.16	1.04	0.89	3.07	0.00	1.85	2.82	2.08
NADH dehydrogenase [ubiquinone] flavoprotein 2, mitochondrial OS=Rattus norvegicus GN=Ndufv2 PE=1 SV=2	0.95	2.87	1.08	3.11	0.89	3.07	0.00	2.77	0.94	2.08
Synaptophysin OS=Rattus norvegicus GN=Syp PE=1 SV=1	2.85	1.91	2.16	1.04	0.89	2.04	3.77	0.00	0.00	2.08
Hypoxanthine-guanine phosphoribosyltransferase OS=Rattus norvegicus GN=Hprt1 PE=1 SV=1	2.85	3.83	1.08	2.07	0.89	2.04	3.77	1.85	0.94	2.08
Excitatory amino acid transporter 1 OS=Rattus norvegicus GN=Slc1a3 PE=1 SV=2	0.00	0.96	1.08	0.00	2.66	2.04	1.26	1.85	2.82	1.04
N(G),N(G)-dimethylarginine dimethylaminohydrolase 2 OS=Rattus norvegicus GN=Ddah2 PE=1 SV=1	0.95	3.83	0.00	2.07	1.77	2.04	2.51	0.92	0.94	2.08
Ezrin OS=Rattus norvegicus GN=Ezr PE=1 SV=3	2.85	0.96	0.00	2.07	2.66	2.04	0.00	0.92	1.88	1.04
UMP-CMP kinase OS=Rattus norvegicus GN=Cmpk1 PE=1 SV=2	1.90	3.83	4.32	1.04	1.77	5.11	0.00	0.92	0.94	2.08
Lamin-B1 OS=Rattus norvegicus GN=Lmnb1 PE=1 SV=3	4.75	2.87	1.08	2.07	2.66	2.04	0.00	2.77	0.00	4.15
Nucleosome assembly protein 1-like 1 OS=Rattus norvegicus GN=Nap1l1 PE=2 SV=1	0.00	0.96	2.16	2.07	0.89	2.04	2.51	1.85	0.94	3.12
Vesicle-associated membrane protein 2 OS=Rattus norvegicus GN=Vamp2 PE=1 SV=2	0.95	4.79	1.08	0.00	0.89	3.07	0.00	2.77	5.63	3.12
Spliceosome RNA helicase Ddx39b OS=Rattus norvegicus GN=Ddx39b PE=1 SV=3	0.00	0.00	3.24	0.00	0.89	0.00	1.26	0.00	0.94	0.00
Alpha-adducin OS=Rattus norvegicus GN=Add1 PE=1 SV=2	1.90	1.91	2.16	3.11	2.66	1.02	1.26	2.77	0.94	3.12
Contactin-1 OS=Rattus norvegicus GN=Cntn1 PE=1 SV=2	0.00	2.87	1.08	0.00	3.55	0.00	0.00	1.85	0.94	0.00
Tropomodulin-2 OS=Rattus norvegicus GN=Tmod2 PE=1 SV=1	0.95	2.87	1.08	0.00	2.66	2.04	2.51	1.85	0.94	2.08
Myosin-9 OS=Rattus norvegicus GN=Myh9 PE=1 SV=3	1.90	0.96	1.08	0.00	0.89	0.00	0.00	0.92	1.88	1.04
Gelsolin OS=Rattus norvegicus GN=Gsn PE=1 SV=1	1.90	1.91	3.24	2.07	1.77	0.00	1.26	1.85	2.82	3.12
Calcium/calmodulin-dependent protein kinase type II subunit beta OS=Rattus norvegicus GN=Camk2b PE=1 SV=1	0.95	2.87	2.16	4.15	3.55	2.04	2.51	6.46	3.76	2.08
Paralemmin-1 OS=Rattus norvegicus GN=Palm PE=1 SV=1	0.00	0.96	1.08	0.00	2.66	2.04	2.51	2.77	1.88	1.04
6-phosphofructokinase type C OS=Rattus norvegicus GN=Pfkp PE=1 SV=2	2.85	0.96	2.16	3.11	1.77	0.00	1.26	0.92	1.88	1.04
Superoxide dismutase [Mn], mitochondrial OS=Rattus norvegicus GN=Sod2 PE=1 SV=2	1.90	1.91	0.00	1.04	1.77	2.04	0.00	1.85	1.88	2.08
Protein lin-7 homolog A OS=Rattus norvegicus GN=Lin7a PE=1 SV=2	0.00	1.91	2.16	1.04	1.77	0.00	0.00	0.00	0.00	1.04
Purkinje cell protein 4 OS=Rattus norvegicus GN=Pcp4 PE=1 SV=2	2.85	1.91	3.24	2.07	1.77	2.04	0.00	2.77	1.88	2.08
Cytochrome c oxidase subunit 4 isoform 1, mitochondrial OS=Rattus norvegicus GN=Cox4i1 PE=1 SV=1	0.00	1.91	4.32	4.15	0.89	2.04	3.77	1.85	1.88	0.00
Nucleoside diphosphate kinase B OS=Rattus norvegicus GN=Nme2 PE=1 SV=1	0.00	0.00	3.24	0.00	0.89	0.00	1.26	0.00	0.00	0.00
Long-chain-fatty-acid--CoA ligase 6 OS=Rattus norvegicus GN=Acsl6 PE=1 SV=1	1.90	0.00	1.08	0.00	1.77	3.07	0.00	1.85	2.82	1.04
Fatty acid-binding protein, epidermal OS=Rattus norvegicus GN=Fabp5 PE=1 SV=3	2.85	0.00	0.00	0.00	0.89	0.00	1.26	0.92	2.82	1.04
Isocitrate dehydrogenase [NAD] subunit alpha, mitochondrial OS=Rattus norvegicus GN=Idh3a PE=1 SV=1	1.90	1.91	2.16	2.07	1.77	2.04	2.51	1.85	1.88	2.08
Neurofilament light polypeptide OS=Rattus norvegicus GN=Nefl PE=1 SV=3	3.80	2.87	1.08	2.07	2.66	2.04	2.51	4.62	1.88	4.15
AP-2 complex subunit mu OS=Rattus norvegicus GN=Ap2m1 PE=1 SV=1	0.00	0.96	2.16	0.00	0.89	0.00	1.26	0.92	0.94	0.00
Alpha-centractin OS=Rattus norvegicus GN=Actr1a PE=1 SV=1	2.85	0.96	2.16	3.11	0.89	2.04	0.00	2.77	1.88	2.08
Heterogeneous nuclear ribonucleoprotein D0 OS=Rattus norvegicus GN=Hnrnpd PE=1 SV=1	3.80	0.96	2.16	2.07	1.77	2.04	2.51	1.85	2.82	0.00

	Quantitative Value of Spectra Counts									
	METH treated OB samples					Control				
Identified Proteins (336)	OM4	OM5	OM6	OM7	OM8	OS2	OS3	OS4	OS5	OS6
Kinesin-1 heavy chain OS=Rattus norvegicus GN=Kif5b PE=2 SV=1	0.95	0.96	1.08	1.04	1.77	2.04	0.00	1.85	2.82	2.08
Thioredoxin-dependent peroxide reductase, mitochondrial OS=Rattus norvegicus GN=Prdx3 PE=1 SV=2	2.85	0.96	3.24	2.07	1.77	1.02	1.26	1.85	1.88	0.00
Receptor-type tyrosine-protein phosphatase zeta OS=Rattus norvegicus GN=Ptprz1 PE=1 SV=1	0.00	1.91	2.16	2.07	0.00	0.00	1.26	0.00	0.00	0.00
Cytochrome b-c1 complex subunit 6, mitochondrial OS=Rattus norvegicus GN=Uqcrh PE=2 SV=1	0.95	1.91	0.00	0.00	0.89	1.02	3.77	0.00	0.94	0.00
Acidic leucine-rich nuclear phosphoprotein 32 family member A OS=Rattus norvegicus GN=Anp32a PE=2 SV=1	0.95	0.96	3.24	1.04	0.89	2.04	0.00	1.85	1.88	1.04
Ras-related protein Rab-2A OS=Rattus norvegicus GN=Rab2a PE=1 SV=1	1.90	1.91	3.24	1.04	2.66	1.02	2.51	1.85	0.94	0.00
Glutathione S-transferase alpha-3 OS=Rattus norvegicus GN=Gsta3 PE=1 SV=3	0.00	0.96	0.00	1.04	0.89	2.04	1.26	0.92	4.69	1.04
Heterogeneous nuclear ribonucleoprotein H OS=Rattus norvegicus GN=Hnrnph1 PE=1 SV=2	0.00	0.00	1.08	4.15	1.77	2.04	1.26	1.85	0.94	1.04
Keratin, type II cytoskeletal 6A OS=Rattus norvegicus GN=Krt6a PE=1 SV=1	2.85	0.00	1.08	0.00	0.00	4.09	1.26	3.69	1.88	1.04
Histone H1.4 OS=Rattus norvegicus GN=Hist1h1e PE=1 SV=3	0.00	0.00	0.00	2.07	2.66	6.13	5.02	1.85	3.76	0.00
Heterogeneous nuclear ribonucleoproteins A2/B1 OS=Rattus norvegicus GN=Hnrnpa2b1 PE=1 SV=1	1.90	1.91	2.16	2.07	0.89	0.00	2.51	0.00	2.82	0.00
ProSAAS OS=Rattus norvegicus GN=Pcsk1n PE=1 SV=1	1.90	1.91	3.24	1.04	1.77	2.04	1.26	1.85	2.82	1.04
Elongation factor Tu, mitochondrial OS=Rattus norvegicus GN=Tufm PE=1 SV=1	0.95	0.96	1.08	0.00	0.89	2.04	0.00	2.77	1.88	0.00
Lactoylglutathione lyase OS=Rattus norvegicus GN=Glo1 PE=1 SV=3	2.85	0.96	3.24	0.00	0.89	2.04	2.51	1.85	1.88	1.04
Adenylyl cyclase-associated protein 1 OS=Rattus norvegicus GN=Cap1 PE=1 SV=3	1.90	1.91	1.08	2.07	1.77	2.04	1.26	0.92	0.00	0.00
Synaptic vesicle glycoprotein 2A OS=Rattus norvegicus GN=Sv2a PE=1 SV=2	0.95	2.87	0.00	0.00	1.77	4.09	0.00	1.85	2.82	1.04
Histone H3.3 OS=Rattus norvegicus GN=H3f3b PE=1 SV=2	0.95	0.00	1.08	4.15	0.00	1.02	1.26	1.85	1.88	1.04
Thy-1 membrane glycoprotein OS=Rattus norvegicus GN=Thy1 PE=1 SV=1	1.90	0.00	1.08	0.00	0.00	3.07	0.00	0.00	0.94	1.04
Pyridoxal kinase OS=Rattus norvegicus GN=Pdxk PE=1 SV=1	0.95	1.91	3.24	1.04	1.77	2.04	0.00	0.92	1.88	1.04
G-protein-signaling modulator 1 OS=Rattus norvegicus GN=Gpsm1 PE=1 SV=2	0.95	2.87	2.16	1.04	0.89	0.00	0.00	2.77	0.94	1.04
Voltage-dependent anion-selective channel protein 2 OS=Rattus norvegicus GN=Vdac2 PE=1 SV=2	0.00	1.91	1.08	0.00	1.77	2.04	2.51	0.00	0.94	0.00
Profilin-1 OS=Rattus norvegicus GN=Pfn1 PE=1 SV=2	2.85	0.00	2.16	1.04	1.77	3.07	1.26	0.92	0.00	3.12
Endoplasmin OS=Rattus norvegicus GN=Hsp90b1 PE=1 SV=2	3.80	2.87	2.16	3.11	2.66	3.07	1.26	2.77	2.82	3.12
Cell division control protein 42 homolog OS=Rattus norvegicus GN=Cdc42 PE=1 SV=2	0.00	0.96	1.08	1.04	0.89	1.02	0.00	1.85	0.00	2.08
Guanine nucleotide-binding protein G(i) subunit alpha-2 OS=Rattus norvegicus GN=Gnai2 PE=1 SV=3	3.80	3.83	2.16	3.11	5.32	1.02	2.51	0.92	1.88	2.08
Prohibitin-2 OS=Rattus norvegicus GN=Phb2 PE=1 SV=1	0.95	1.91	3.24	0.00	2.66	1.02	1.26	0.00	2.82	1.04
Transcriptional activator protein Pur-beta OS=Rattus norvegicus GN=Purb PE=1 SV=3	2.85	0.96	0.00	3.11	1.77	1.02	1.26	1.85	1.88	2.08
Endophilin-B2 OS=Rattus norvegicus GN=Sh3glb2 PE=2 SV=2	0.95	0.00	0.00	1.04	0.00	1.02	1.26	0.92	0.00	2.08
Neurofilament medium polypeptide OS=Rattus norvegicus GN=Nefm PE=1 SV=4	1.90	1.91	2.16	3.11	0.00	1.02	3.77	2.77	2.82	3.12
3-ketoacyl-CoA thiolase, mitochondrial OS=Rattus norvegicus GN=Acaa2 PE=1 SV=1	0.95	2.87	2.16	0.00	1.77	2.04	0.00	1.85	0.94	0.00
EF-hand domain-containing protein D2 OS=Rattus norvegicus GN=Efhd2 PE=1 SV=1	0.00	0.00	1.08	2.07	2.66	0.00	2.51	0.92	0.94	0.00
Eukaryotic initiation factor 4A-II OS=Rattus norvegicus GN=Eif4a2 PE=1 SV=1	0.95	1.91	1.08	0.00	1.77	0.00	0.00	0.92	1.88	1.04
Basigin OS=Rattus norvegicus GN=Bsg PE=1 SV=2	2.85	0.00	3.24	2.07	1.77	1.02	1.26	1.85	0.00	2.08
Ubiquitin-conjugating enzyme E2 variant 2 OS=Rattus norvegicus GN=Ube2v2 PE=1 SV=3	1.90	0.96	3.24	1.04	0.89	0.00	0.00	0.92	0.00	3.12
NADH dehydrogenase [ubiquinone] iron-sulfur protein 2, mitochondrial OS=Rattus norvegicus GN=Ndufs2 PE=1 SV=1	0.00	0.96	0.00	0.00	1.77	0.00	0.00	0.92	0.94	3.12
Clathrin light chain B OS=Rattus norvegicus GN=Cltb PE=1 SV=1	2.85	0.00	1.08	0.00	0.00	0.00	1.26	0.00	3.76	2.08
Keratin, type I cytoskeletal 10 OS=Rattus norvegicus GN=Krt10 PE=2 SV=1	1.90	1.91	0.00	0.00	0.00	4.09	0.00	3.69	0.94	0.00
Prostaglandin E synthase 3 OS=Rattus norvegicus GN=Ptges3 PE=1 SV=2	2.85	0.96	2.16	1.04	0.00	1.02	1.26	0.92	1.88	0.00
Myosin light polypeptide 6 OS=Rattus norvegicus GN=Myl6 PE=1 SV=3	0.00	2.87	2.16	2.07	1.77	1.02	1.26	1.85	0.00	1.04
Ras-related protein Rab-11B OS=Rattus norvegicus GN=Rab11b PE=2 SV=4	0.00	0.96	1.08	0.00	1.77	2.04	2.51	1.85	1.88	0.00
Drebrin OS=Rattus norvegicus GN=Dbn1 PE=2 SV=3	0.95	1.91	3.24	1.04	0.89	2.04	0.00	1.85	0.00	2.08
Amyloid beta A4 protein OS=Rattus norvegicus GN=App PE=1 SV=2	2.85	0.00	1.08	1.04	0.89	2.04	2.51	0.00	0.00	2.08
Alpha-2-HS-glycoprotein OS=Rattus norvegicus GN=Ahsg PE=1 SV=2	0.00	0.96	0.00	0.00	0.00	2.04	3.77	2.77	1.88	4.15
Matrin-3 OS=Rattus norvegicus GN=Matr3 PE=1 SV=2	1.90	2.87	0.00	2.07	0.89	1.02	0.00	0.00	0.94	1.04
Ubiquitin-conjugating enzyme E2 N OS=Rattus norvegicus GN=Ube2n PE=1 SV=1	0.95	0.96	0.00	1.04	0.89	1.02	0.00	2.77	0.00	0.00
Acetyl-CoA acetyltransferase, mitochondrial OS=Rattus norvegicus GN=Acat1 PE=1 SV=1	1.90	0.96	0.00	0.00	2.66	0.00	0.00	1.85	0.00	2.08

	Quantitative Value of Spectra Counts									
	METH treated OB samples					Control				
Identified Proteins (336)	OM4	OM5	OM6	OM7	OM8	OS2	OS3	OS4	OS5	OS6
Ribonuclease inhibitor OS=Rattus norvegicus GN=Rnh1 PE=1 SV=2	3.80	0.00	0.00	1.04	0.89	2.04	1.26	1.85	0.94	1.04
4F2 cell-surface antigen heavy chain OS=Rattus norvegicus GN=Slc3a2 PE=1 SV=1	1.90	1.91	1.08	0.00	1.77	2.04	0.00	1.85	0.94	0.00
Serine/threonine-protein phosphatase 2A catalytic subunit beta isoform OS=Rattus norvegicus GN=Ppp2cb PE=1 SV=1	2.85	0.00	0.00	0.00	1.77	2.04	0.00	0.00	0.94	1.04
Ras-related protein Rab-1B OS=Rattus norvegicus GN=Rab1b PE=1 SV=1	1.90	1.91	4.32	3.11	2.66	3.07	3.77	2.77	4.69	2.08
Protein disulfide-isomerase OS=Rattus norvegicus GN=P4hb PE=1 SV=2	0.95	1.91	2.16	0.00	0.00	0.00	0.00	1.85	0.94	1.04
Ras-related protein Ral-A OS=Rattus norvegicus GN=Rala PE=1 SV=1	0.95	0.96	1.08	0.00	0.89	2.04	0.00	2.77	1.88	2.08
Dynactin subunit 1 OS=Rattus norvegicus GN=Dctn1 PE=2 SV=2	0.95	0.96	0.00	0.00	0.89	2.04	0.00	0.00	0.94	2.08
26S protease regulatory subunit 6A OS=Rattus norvegicus GN=Psmc3 PE=2 SV=1	0.95	1.91	1.08	0.00	1.77	2.04	0.00	0.00	0.94	3.12
Carbonyl reductase [NADPH] 1 OS=Rattus norvegicus GN=Cbr1 PE=1 SV=2	0.95	1.91	1.08	1.04	0.00	0.00	1.26	2.77	1.88	2.08
Protein phosphatase 1E OS=Rattus norvegicus GN=Ppm1e PE=2 SV=1	1.90	0.00	1.08	2.07	0.89	2.04	0.00	0.00	1.88	3.12
F-actin-capping protein subunit alpha-2 OS=Rattus norvegicus GN=Capza2 PE=1 SV=1	1.90	0.00	0.00	1.04	1.77	2.04	0.00	1.85	0.00	2.08
Dihydropyrimidinase-related protein 5 OS=Rattus norvegicus GN=Dpysl5 PE=1 SV=1	0.95	1.91	1.08	1.04	1.77	0.00	0.00	0.92	0.00	0.00
Nuclear migration protein nudC OS=Rattus norvegicus GN=Nudc PE=1 SV=1	1.90	1.91	1.08	0.00	1.77	2.04	0.00	0.92	1.88	0.00
Ras-related protein Rab-14 OS=Rattus norvegicus GN=Rab14 PE=1 SV=3	1.90	2.87	3.24	2.07	1.77	3.07	2.51	2.77	4.69	1.04
Calcineurin subunit B type 1 OS=Rattus norvegicus GN=Ppp3r1 PE=1 SV=2	1.90	1.91	3.24	1.04	0.89	0.00	0.00	0.92	0.94	0.00
Serine/threonine-protein phosphatase 2A 65 kDa regulatory subunit A beta isoform OS=Rattus norvegicus GN=Ppp2r1b PE=2 SV=1	0.00	0.96	3.24	1.04	2.66	0.00	1.26	0.92	0.94	0.00
Isocitrate dehydrogenase [NAD] subunit gamma 1, mitochondrial OS=Rattus norvegicus GN=Idh3g PE=2 SV=2	3.80	0.96	1.08	1.04	0.89	1.02	0.00	1.85	0.94	0.00
Glutathione S-transferase Mu 5 OS=Rattus norvegicus GN=Gstm5 PE=1 SV=3	0.95	0.96	0.00	2.07	0.89	1.02	0.00	0.92	1.88	0.00
Cell adhesion molecule 3 OS=Rattus norvegicus GN=Cadm3 PE=2 SV=1	2.85	0.00	1.08	1.04	0.89	0.00	0.00	0.00	1.88	0.00
ATP synthase subunit delta, mitochondrial OS=Rattus norvegicus GN=Atp5d PE=1 SV=2	0.95	0.96	0.00	2.07	1.77	0.00	2.51	2.77	0.00	2.08
Hemoglobin subunit beta-2 OS=Rattus norvegicus PE=1 SV=2	17.12	15.32	14.05	15.56	15.96	16.35	22.60	15.70	15.96	15.58
Glutathione S-transferase Mu 1 OS=Rattus norvegicus GN=Gstm1 PE=1 SV=2	0.00	0.00	4.32	0.00	0.00	0.00	3.77	1.85	1.88	0.00
Stathmin OS=Rattus norvegicus GN=Stmn1 PE=1 SV=2	0.00	2.87	3.24	2.07	0.00	1.02	2.51	0.00	0.00	0.00
Platelet-activating factor acetylhydrolase IB subunit alpha OS=Rattus norvegicus GN=Pafah1b1 PE=1 SV=2	1.90	0.96	1.08	0.00	0.89	0.00	1.26	0.00	0.94	1.04
Protein disulfide-isomerase A6 OS=Rattus norvegicus GN=Pdia6 PE=1 SV=2	0.95	1.91	0.00	1.04	0.00	2.04	0.00	0.00	0.94	2.08
Eukaryotic translation initiation factor 5A-1 OS=Rattus norvegicus GN=Eif5a PE=1 SV=3	1.90	0.96	1.08	1.04	0.00	0.00	0.00	0.00	0.94	0.00
Annexin A3 OS=Rattus norvegicus GN=Anxa3 PE=1 SV=4	0.00	0.00	0.00	1.04	0.00	3.07	2.51	1.85	0.94	2.08
Unconventional myosin-Va OS=Rattus norvegicus GN=Myo5a PE=1 SV=1	0.95	0.00	0.00	1.04	1.77	0.00	0.00	0.00	0.00	2.08
40S ribosomal protein SA OS=Rattus norvegicus GN=Rpsa PE=1 SV=3	0.95	1.91	1.08	2.07	0.00	0.00	2.51	0.92	0.94	1.04
Protein IMPACT OS=Rattus norvegicus GN=Impact PE=2 SV=1	0.00	0.00	1.08	0.00	0.00	0.00	0.00	1.85	0.00	0.00
Pyruvate carboxylase, mitochondrial OS=Rattus norvegicus GN=Pc PE=1 SV=2	0.95	1.91	0.00	0.00	1.77	0.00	0.00	0.92	1.88	1.04
Transgelin OS=Rattus norvegicus GN=Tagln PE=1 SV=2	0.00	1.91	2.16	1.04	0.00	2.04	0.00	0.92	0.00	0.00
Keratin, type II cytoskeletal 1 OS=Rattus norvegicus GN=Krt1 PE=2 SV=1	1.90	0.00	0.00	0.00	0.00	4.09	0.00	1.85	0.94	0.00
Transgelin-3 OS=Rattus norvegicus GN=Tagln3 PE=1 SV=2	1.90	0.96	0.00	2.07	0.89	0.00	1.26	0.92	0.00	0.00
Importin subunit beta-1 OS=Rattus norvegicus GN=Kpnb1 PE=1 SV=1	1.90	0.96	0.00	0.00	0.89	0.00	0.00	0.00	0.94	2.08
T-complex protein 1 subunit beta OS=Rattus norvegicus GN=Cct2 PE=1 SV=3	1.90	0.00	2.16	0.00	0.89	0.00	0.00	0.92	0.94	2.08
Protein RUFY3 OS=Rattus norvegicus GN=Rufy3 PE=1 SV=1	0.95	0.00	0.00	1.04	0.00	0.00	0.00	1.85	0.94	2.08
Protein S100-B OS=Rattus norvegicus GN=S100b PE=1 SV=2	0.95	0.00	1.08	2.07	0.89	0.00	0.00	0.92	1.88	1.04
ADP/ATP translocase 1 OS=Rattus norvegicus GN=Slc25a4 PE=1 SV=3	1.90	2.87	1.08	2.07	3.55	2.04	0.00	1.85	3.76	2.08
Kinesin-like protein KIF2A OS=Rattus norvegicus GN=Kif2a PE=2 SV=2	0.95	0.96	1.08	2.07	0.89	0.00	0.00	0.92	0.00	0.00
Alpha-1-antiproteinase OS=Rattus norvegicus GN=Serpina1 PE=1 SV=2	0.00	0.00	1.08	0.00	2.66	0.00	1.26	0.00	0.00	1.04
Beta-synuclein OS=Rattus norvegicus GN=Sncb PE=1 SV=1	1.90	1.91	0.00	0.00	0.00	2.04	1.26	0.92	0.00	0.00
Myosin-10 OS=Rattus norvegicus GN=Myh10 PE=1 SV=1	0.00	0.00	1.08	0.00	0.00	2.04	0.00	0.00	0.00	0.00
Cyclin-dependent kinase 5 OS=Rattus norvegicus GN=Cdk5 PE=1 SV=1	1.90	0.00	0.00	2.07	0.89	1.02	0.00	0.92	0.94	0.00
Neuronal migration protein doublecortin OS=Rattus norvegicus GN=Dcx PE=1 SV=2	0.95	0.00	0.00	1.04	0.89	0.00	0.00	0.00	0.94	3.12
Adenosylhomocysteinase OS=Rattus norvegicus GN=Ahcy PE=1 SV=3	0.95	3.83	1.08	0.00	0.89	0.00	0.00	0.00	0.00	0.00

	Quantitative Value of Spectra Counts									
	METH treated OB samples					Control				
Identified Proteins (336)	OM4	OM5	OM6	OM7	OM8	OS2	OS3	OS4	OS5	OS6
D-3-phosphoglycerate dehydrogenase OS=Rattus norvegicus GN=Phgdh PE=1 SV=3	0.00	0.00	0.00	2.07	1.77	0.00	0.00	0.00	0.00	0.00
Trifunctional enzyme subunit alpha, mitochondrial OS=Rattus norvegicus GN=Hadha PE=1 SV=2	0.00	0.96	0.00	0.00	0.00	1.02	0.00	1.85	0.00	1.04
Oxidation resistance protein 1 OS=Rattus norvegicus GN=Oxr1 PE=1 SV=3	0.00	1.91	0.00	2.07	0.00	1.02	0.00	0.00	0.94	0.00
Synaptogyrin-1 OS=Rattus norvegicus GN=Syngr1 PE=2 SV=1	0.95	0.00	0.00	0.00	0.00	1.02	0.00	0.00	1.88	1.04
Proteasome subunit alpha type-4 OS=Rattus norvegicus GN=Psma4 PE=1 SV=1	1.90	0.00	0.00	0.00	0.00	2.04	0.00	1.85	0.94	0.00
Myosin regulatory light chain 12B OS=Rattus norvegicus GN=Myl12b PE=1 SV=3	0.00	0.00	0.00	1.04	0.89	0.00	2.51	0.92	0.00	0.00
Cytosolic non-specific dipeptidase OS=Rattus norvegicus GN=Cndp2 PE=1 SV=1	0.95	0.00	0.00	0.00	1.77	2.04	0.00	0.00	0.00	0.00
Alpha-1-macroglobulin OS=Rattus norvegicus GN=A1m PE=1 SV=1	0.00	0.00	0.00	1.04	1.77	0.00	0.00	0.00	0.00	0.00
Proteasome subunit alpha type-7 OS=Rattus norvegicus GN=Psma7 PE=1 SV=1	0.00	0.96	0.00	0.00	0.00	1.02	0.00	1.85	0.00	0.00
Electron transfer flavoprotein subunit beta OS=Rattus norvegicus GN=Etfb PE=2 SV=3	0.00	0.00	2.16	0.00	0.89	0.00	0.00	0.00	0.00	2.08
3-hydroxyacyl-CoA dehydrogenase type-2 OS=Rattus norvegicus GN=Hsd17b10 PE=1 SV=3	0.00	0.00	0.00	0.00	0.00	0.00	0.00	1.85	0.00	0.00
ATP synthase subunit b, mitochondrial OS=Rattus norvegicus GN=Atp5f1 PE=1 SV=1	0.00	0.00	0.00	0.00	0.00	0.00	3.77	0.00	0.00	1.04
T-complex protein 1 subunit gamma OS=Rattus norvegicus GN=Cct3 PE=1 SV=1	0.00	1.91	0.00	2.07	0.00	0.00	0.00	0.00	0.00	0.00
Gamma-glutamyltransferase 7 OS=Rattus norvegicus GN=Ggt7 PE=2 SV=2	0.00	0.00	0.00	0.00	1.77	0.00	0.00	0.00	0.00	0.00
ATP synthase subunit d, mitochondrial OS=Rattus norvegicus GN=Atp5h PE=1 SV=3	0.00	0.00	0.00	2.07	0.00	0.00	0.00	0.00	0.00	0.00

OM: METH treated olfactory bulb tissue, OS: Saline injected olfactory bulb tissue.
