# Supplementary material for: The Effect of Chronic Methamphetamine Exposure on the Hippocampal and Olfactory Bulb Neuroproteomes of Rats
Source: PLoS One. 2016 Apr 15;11(4):e0151034. doi: 10.1371/journal.pone.0151034 (PMC4833297; doi:10.1371/journal.pone.0151034)
Supplement: S5 Table — (PDF) [file pone.0151034.s006.pdf]

Table S5. Target proteins and transitions of hippcampal proteome

Target protein	Selected peptide	Precursor *m/z*	Charge state of precursor	Transitions *m/z*	Charge state of transition	Ion type of transition
GABT_RAT=57160.70;4-aminobutyrate aminotransferase; mitochondrial OS=Rattus norvegicus GN=Abat PE=1 SV=3	NLLLAEVINIIK	676.929	2	487.323	1	y4
GABT_RAT=57160.70;4-aminobutyrate aminotransferase; mitochondrial OS=Rattus norvegicus GN=Abat PE=1 SV=3	NLLLAEVINIIK	676.929	2	899.556	1	y8
GABT_RAT=57160.70;4-aminobutyrate aminotransferase; mitochondrial OS=Rattus norvegicus GN=Abat PE=1 SV=3	NLLLAEVINIIK	676.929	2	1012.640	1	y9
GABT_RAT=57160.70;4-aminobutyrate aminotransferase; mitochondrial OS=Rattus norvegicus GN=Abat PE=1 SV=3	IDIPSFDWPIAPFPR	885.964	2	516.292	1	y4
GABT_RAT=57160.70;4-aminobutyrate aminotransferase; mitochondrial OS=Rattus norvegicus GN=Abat PE=1 SV=3	IDIPSFDWPIAPFPR	885.964	2	587.330	1	y5
GABT_RAT=57160.70;4-aminobutyrate aminotransferase; mitochondrial OS=Rattus norvegicus GN=Abat PE=1 SV=3	IDIPSFDWPIAPFPR	885.964	2	797.466	1	y7
CH60_RAT=61088.45;60 kDa heat shock protein; mitochondrial OS=Rattus norvegicus GN=Hspd1 PE=1 SV=1	ISSVQSIVPALEIANAHR	635.690	3	695.893	2	y13
CH60_RAT=61088.45;60 kDa heat shock protein; mitochondrial OS=Rattus norvegicus GN=Hspd1 PE=1 SV=1	ISSVQSIVPALEIANAHR	635.690	3	759.923	2	y14
CH60_RAT=61088.45;60 kDa heat shock protein; mitochondrial OS=Rattus norvegicus GN=Hspd1 PE=1 SV=1	ISSVQSIVPALEIANAHR	635.690	3	852.973	2	y16
CH60_RAT=61088.45;60 kDa heat shock protein; mitochondrial OS=Rattus norvegicus GN=Hspd1 PE=1 SV=1	LVQDVANNTNEEAGDGTTTATVLAR	854.088	3	458.308	1	y4
CH60_RAT=61088.45;60 kDa heat shock protein; mitochondrial OS=Rattus norvegicus GN=Hspd1 PE=1 SV=1	LVQDVANNTNEEAGDGTTTATVLAR	854.088	3	832.488	1	y8
CH60_RAT=61088.45;60 kDa heat shock protein; mitochondrial OS=Rattus norvegicus GN=Hspd1 PE=1 SV=1	LVQDVANNTNEEAGDGTTTATVLAR	854.088	3	1162.606	1	y12
CAPS1_RAT=147313.66;Calcium-dependent secretion activator 1 OS=Rattus norvegicus GN=Cadps PE=1 SV=1	ATLSLLER	451.769	2	417.245	1	y3
CAPS1_RAT=147313.66;Calcium-dependent secretion activator 1 OS=Rattus norvegicus GN=Cadps PE=1 SV=1	ATLSLLER	451.769	2	617.361	1	y5
CAPS1_RAT=147313.66;Calcium-dependent secretion activator 1 OS=Rattus norvegicus GN=Cadps PE=1 SV=1	ATLSLLER	451.769	2	730.445	1	y6
CAPS1_RAT=147313.66;Calcium-dependent secretion activator 1 OS=Rattus norvegicus GN=Cadps PE=1 SV=1	FVTILEGVLAK	595.363	2	487.323	1	y5
CAPS1_RAT=147313.66;Calcium-dependent secretion activator 1 OS=Rattus norvegicus GN=Cadps PE=1 SV=1	FVTILEGVLAK	595.363	2	729.450	1	y7
CAPS1_RAT=147313.66;Calcium-dependent secretion activator 1 OS=Rattus norvegicus GN=Cadps PE=1 SV=1	FVTILEGVLAK	595.363	2	943.582	1	y9
BASP1_RAT=21777.44;Brain acid soluble protein 1 OS=Rattus norvegicus GN=Basp1 PE=1 SV=2	ESEPQAAADATEVK	723.341	2	550.783	2	y11
BASP1_RAT=21777.44;Brain acid soluble protein 1 OS=Rattus norvegicus GN=Basp1 PE=1 SV=2	ESEPQAAADATEVK	723.341	2	662.335	1	y6
BASP1_RAT=21777.44;Brain acid soluble protein 1 OS=Rattus norvegicus GN=Basp1 PE=1 SV=2	ESEPQAAADATEVK	723.341	2	733.372	1	y7
BASP1_RAT=21777.44;Brain acid soluble protein 1 OS=Rattus norvegicus GN=Basp1 PE=1 SV=2	AGEASAESTGAADGAPQEEGEAK	1066.964	2	887.410	1	y8
BASP1_RAT=21777.44;Brain acid soluble protein 1 OS=Rattus norvegicus GN=Basp1 PE=1 SV=2	AGEASAESTGAADGAPQEEGEAK	1066.964	2	1015.469	1	y10
BASP1_RAT=21777.44;Brain acid soluble protein 1 OS=Rattus norvegicus GN=Basp1 PE=1 SV=2	AGEASAESTGAADGAPQEEGEAK	1066.964	2	1130.495	1	y11
ARP2_RAT=44990.33;Actin-related protein 2 OS=Rattus norvegicus GN=Actr2 PE=1 SV=1	GYAFNHSADFETVR	807.371	2	651.346	1	y5
ARP2_RAT=44990.33;Actin-related protein 2 OS=Rattus norvegicus GN=Actr2 PE=1 SV=1	GYAFNHSADFETVR	807.371	2	924.442	1	y8
ARP2_RAT=44990.33;Actin-related protein 2 OS=Rattus norvegicus GN=Actr2 PE=1 SV=1	GYAFNHSADFETVR	807.371	2	1061.500	1	y9
ARP2_RAT=44990.33;Actin-related protein 2 OS=Rattus norvegicus GN=Actr2 PE=1 SV=1	LC[Carboxyamidomethyl]YVGYNIEQEQK	822.390	2	888.442	1	y7
ARP2_RAT=44990.33;Actin-related protein 2 OS=Rattus norvegicus GN=Actr2 PE=1 SV=1	LC[Carboxyamidomethyl]YVGYNIEQEQK	822.390	2	1108.526	1	y9
ARP2_RAT=44990.33;Actin-related protein 2 OS=Rattus norvegicus GN=Actr2 PE=1 SV=1	LC[Carboxyamidomethyl]YVGYNIEQEQK	822.390	2	1207.595	1	y10
ARC1A_RAT=42142.89;Actin-related protein 2/3 complex subunit 1A OS=Rattus norvegicus GN=Arpc1a PE=2 SV=1	LAWVSHDSTVSVADASK	886.944	2	677.346	1	y7
ARC1A_RAT=42142.89;Actin-related protein 2/3 complex subunit 1A OS=Rattus norvegicus GN=Arpc1a PE=2 SV=1	LAWVSHDSTVSVADASK	886.944	2	1079.521	1	y11
ARC1A_RAT=42142.89;Actin-related protein 2/3 complex subunit 1A OS=Rattus norvegicus GN=Arpc1a PE=2 SV=1	LAWVSHDSTVSVADASK	886.944	2	1303.612	1	y13
ARC1A_RAT=42142.89;Actin-related protein 2/3 complex subunit 1A OS=Rattus norvegicus GN=Arpc1a PE=2 SV=1	DGIWKPTLVILR	470.954	3	401.287	1	y3
ARC1A_RAT=42142.89;Actin-related protein 2/3 complex subunit 1A OS=Rattus norvegicus GN=Arpc1a PE=2 SV=1	DGIWKPTLVILR	470.954	3	500.355	1	y4
ARC1A_RAT=42142.89;Actin-related protein 2/3 complex subunit 1A OS=Rattus norvegicus GN=Arpc1a PE=2 SV=1	DGIWKPTLVILR	470.954	3	563.361	2	y9
AINX_RAT=56252.70;Alpha-internexin OS=Rattus norvegicus GN=Ina PE=1 SV=2	DVDGATLAR	459.238	2	531.324	1	y5
AINX_RAT=56252.70;Alpha-internexin OS=Rattus norvegicus GN=Ina PE=1 SV=2	DVDGATLAR	459.238	2	588.346	1	y6
AINX_RAT=56252.70;Alpha-internexin OS=Rattus norvegicus GN=Ina PE=1 SV=2	DVDGATLAR	459.238	2	703.373	1	y7
AINX_RAT=56252.70;Alpha-internexin OS=Rattus norvegicus GN=Ina PE=1 SV=2	ALEAELAALR	528.806	2	430.277	1	y4
AINX_RAT=56252.70;Alpha-internexin OS=Rattus norvegicus GN=Ina PE=1 SV=2	ALEAELAALR	528.806	2	543.361	1	y5
AINX_RAT=56252.70;Alpha-internexin OS=Rattus norvegicus GN=Ina PE=1 SV=2	ALEAELAALR	528.806	2	743.441	1	y7
ACTZ_RAT=42700.95;Alpha-centractin OS=Rattus norvegicus GN=Actr1a PE=1 SV=1	AGFAGDQIPK	502.264	2	438.234	2	y8
ACTZ_RAT=42700.95;Alpha-centractin OS=Rattus norvegicus GN=Actr1a PE=1 SV=1	AGFAGDQIPK	502.264	2	657.356	1	y6
ACTZ_RAT=42700.95;Alpha-centractin OS=Rattus norvegicus GN=Actr1a PE=1 SV=1	AGFAGDQIPK	502.264	2	728.393	1	y7
ACTZ_RAT=42700.95;Alpha-centractin OS=Rattus norvegicus GN=Actr1a PE=1 SV=1	LYSTWIGGSILASLDTFK	658.019	3	510.255	1	y4

Target protein	Selected peptide	Precursor *m/z*	Charge state of precursor	Transitions *m/z*	Charge state of transition	Ion type of transition
				710.371	1	y6
				781.409	1	y7
1433G_RAT=28455.98;14-3-3 protein gamma OS=Rattus norvegicus GN=Ywhag PE=1 SV=2	YLAEVATGEK	540.782	2	505.261	1	y5
				733.372	1	y7
				804.409	1	y8
	NVTELNEPLSNEER	822.397	2	715.841	2	y12
				844.415	1	y7
				1087.501	1	y9
1433B_RAT=28150.88;14-3-3 protein beta/alpha OS=Rattus norvegicus GN=Ywhab PE=1 SV=3	YLSEVASGDNK	591.785	2	520.236	1	y5
				690.341	1	y7
				906.416	1	y9
	AVTEQGHELSNEER	799.874	2	634.279	1	y5
				747.363	1	y6
				1070.486	1	y9
DCTN2_RAT=44234.78;Dynactin subunit 2 OS=Rattus norvegicus GN=Dctn2 PE=1 SV=1	LLLQLEATK	514.821	2	448.240	1	y4
				689.382	1	y6
				802.466	1	y7
DPYL1_RAT=62498.73;Dihydropyrimidinase-related protein 1 OS=Rattus norvegicus GN=Crmp1 PE=1 SV=1	NLHQSNFSLSGAQIDDNNPR	1114.028	2	500.257	1	y4
				1000.464	2	y18
				1186.544	1	y11
	KPFPEHLYQR	438.903	3	466.240	1	y3
				471.743	2	y7
				593.804	2	y9
EF2_RAT=96192.24;Elongation factor 2 OS=Rattus norvegicus GN=Eef2 PE=1 SV=4	ETVSEESNVLC[Carboxyamidomethyl]LSK	797.885	2	507.259	1	y4
				620.343	1	y5
				1049.529	1	y9
	YEWDVAEAR	569.762	2	446.235	1	y4
				660.331	1	y6
				846.410	1	y7
GRB2_RAT=25304.48;Growth factor receptor-bound protein 2 OS=Rattus norvegicus GN=Grb2 PE=1 SV=1	YFLWVVK	477.776	2	531.328	1	y4
				644.413	1	y5
				791.481	1	y6
	ESESAPGDFSLSVK	726.846	2	949.498	1	y9
				1020.535	1	y10
				1107.568	1	y11
GSTA3_RAT=25360.33;Glutathione S-transferase alpha-3 OS=Rattus norvegicus GN=Gsta3 PE=1 SV=3	SHGQDYLVGNR	623.302	2	445.251	1	y4
				721.399	1	y6
				1021.506	1	y9
HBB2_RAT=16086.34;Hemoglobin subunit beta-2 OS=Rattus norvegicus PE=1 SV=2	EFTPC[Carboxyamidomethyl]AQAAFQK	699.330	2	510.750	2	y9
				561.274	2	y10
				1020.493	1	y9
	GTFAHLSELHC[Carboxyamidomethyl]DK	757.856	2	422.170	1	y3
				888.388	1	y7
				1001.471	1	y8
HNRPD_RAT=38339.27;Heterogeneous nuclear ribonucleoprotein D0 OS=Rattus norvegicus GN=Hnrnpd PE=1 SV=1	IFVGGLSPDTPEEK	744.883	2	502.250	1	y4
				815.378	1	y7
				902.410	1	y8

Target protein	Selected peptide	Precursor *m/z*	Charge state of precursor	Transitions *m/z*	Charge state of transition	Ion type of transition
	GFGFVLFK	457.760	2	407.265	1	y3
				506.333	1	y4
				710.423	1	y6
HNRPK_RAT=51229.51;Heterogeneous nuclear ribonucleoprotein K OS=Rattus norvegicus GN=Hnrnpk PE=1 SV=1	GSDFDC[Carboxyamidomethyl]ELR	549.730	2	577.276	1	y4
				692.303	1	y5
				839.371	1	y6
	IILDLISESPIK	670.905	2	444.281	1	y4
				660.356	1	y6
				1114.635	1	y10
HSP7C_RAT=71055.30;Heat shock cognate 71 kDa protein OS=Rattus norvegicus GN=Hspa8 PE=1 SV=1	DAGTIAGLNVLR	600.341	2	501.314	1	y4
				671.419	1	y6
				742.456	1	y7
	C[Carboxyamidomethyl]NEIISWLDK	639.313	2	648.335	1	y5
				761.419	1	y6
				874.503	1	y7
IF5A1_RAT=17049.49;Eukaryotic translation initiation factor 5A-1 OS=Rattus norvegicus GN=Eif5a PE=1 SV=3	VHLVGIDIFTGK	649.877	2	850.466	1	y8
				949.535	1	y9
				1062.619	1	y10
	YDC[Carboxyamidomethyl]GEEILITVLSAMTEEAAVAIK	876.104	3	430.302	1	y4
				1062.549	1	y10
				1094.592	2	y21
ROA2_RAT=37511.75;Heterogeneous nuclear ribonucleoproteins A2/B1 OS=Rattus norvegicus GN=Hnrnpa2b1 PE=1 SV=1	GGNFGFGDSR	507.225	2	434.199	1	y4
				581.267	1	y5
				638.289	1	y6
	IDTIEIITDR	594.827	2	504.277	1	y4
				617.361	1	y5
				746.404	1	y6
UCRI_RAT=29712.34;Cytochrome b-c1 complex subunit Rieske; mitochondrial OS=Rattus norvegicus GN=Uqcrfs1 PE=1 SV=2	EIDQEAAVEVSQLR	793.905	2	503.293	1	y4
				731.404	1	y6
				830.473	1	y7
	VPDFSDYR	499.733	2	450.198	2	y7
				802.336	1	y6
				899.389	1	y7
NDKB_RAT=17385.94;Nucleoside diphosphate kinase B OS=Rattus norvegicus GN=Nme2 PE=1 SV=1	GLVGEIIK	414.763	2	502.323	1	y4
				559.344	1	y5
				658.413	1	y6
	NIIHGSDSVESAEK	743.363	2	573.257	2	y11
				1008.447	1	y10
				1145.506	1	y11
STMN1_RAT=17277.94;Stathmin OS=Rattus norvegicus GN=Stmn1 PE=1 SV=2	ESVPEFPLSPPK	663.851	2	506.279	2	y9
				638.387	1	y6
				1011.550	1	y9
	ASGQAFELILSPR	694.880	2	472.287	1	y4
				585.371	1	y5
				974.566	1	y8
NCDN_RAT=80355.24;Neurochondrin OS=Rattus	NDSEQFAALLLVTK	774.917	2	460.312	1	y4

Target protein	Selected peptide	Precursor *m/z*	Charge state of precursor	Transitions *m/z*	Charge state of transition	Ion type of transition
norvegicus GN=Ncdn PE=1 SV=2				757.518	1	y7
				828.555	1	y8
	EGAPSLLC[Carboxyamidomethyl]K	487.752	2	420.227	1	y3
				620.343	1	y5
				717.396	1	y6
HXK1_RAT=103539.56;Hexokinase-1 OS=Rattus norvegicus GN=Hk1 PE=1 SV=4	LALLQVR	406.771	2	402.245	1	y3
				515.330	1	y4
				628.414	1	y5
	LSDEILIDILTR	700.903	2	617.361	1	y5
				730.445	1	y6
				843.529	1	y7
PLPP_RAT=33493.00;Pyridoxal phosphate phosphatase OS=Rattus norvegicus GN=Pdxp PE=1 SV=2	TPGTGSLAAAVETASGR	773.397	2	790.405	1	y8
				861.442	1	y9
				932.479	1	y10
	LETDILFGHR	400.884	3	479.759	2	y8
				516.267	1	y4
				544.280	2	y9
FKB1A_RAT=11972.13;Peptidyl-prolyl cis-trans isomerase FKBP1A OS=Rattus norvegicus GN=Fkbp1a PE=1 SV=3	GVQVETISSGDGR	652.826	2	491.220	1	y5
				578.252	1	y6
				792.384	1	y8
	GWEEGVAQMSVGQR	767.359	2	546.299	1	y5
				805.398	1	y7
				876.435	1	y8
RALA_RAT=23709.02;Ras-related protein Ral-A OS=Rattus norvegicus GN=Rala PE=1 SV=1	ADQWNVNYVETSAK	812.884	2	535.272	1	y5
				911.446	1	y8
				1124.558	1	y10
SNP25_RAT=23528.29;Synaptosomal-associated protein 25 OS=Rattus norvegicus GN=Snap25 PE=1 SV=1	ADQLADESLESTR	717.839	2	492.241	1	y4
				936.426	1	y8
				1007.463	1	y9
	EQMAISGGFIR	604.808	2	549.314	1	y5
				636.346	1	y6
				749.430	1	y7
WDR1_RAT=66824.13;WD repeat-containing protein 1 OS=Rattus norvegicus GN=Wdr1 PE=1 SV=3	YTNLTLR	440.748	2	502.334	1	y4
				616.377	1	y5
				717.425	1	y6
	VYSILGATLK	532.821	2	489.303	1	y5
				602.387	1	y6
				802.503	1	y8
SAP_RAT=62908.24;Sulfated glycoprotein 1 OS=Rattus norvegicus GN=Psap PE=1 SV=1	TC[Carboxyamidomethyl]AWIHDSSLSASC[Carboxyamidomethyl]K	861.882	2	954.419	1	y9
				1091.478	1	y10
				1204.562	1	y11
	EVVDSYLPVILDMIK	867.473	2	928.553	1	y8
				1041.637	1	y9
				1204.700	1	y10
NFASC_RAT=138716.53;Neurofascin OS=Rattus norvegicus GN=Nfasc PE=1 SV=2	TSGAPPESNPSDVK	693.331	2	535.261	2	y10
				545.292	1	y5

Target protein	Selected peptide	Precursor *m/z*	Charge state of precursor	Transitions *m/z*	Charge state of transition	Ion type of transition
				1069.516	1	y10
	GPEPETVIGYSGEDLPSAPR	1036.002	2	527.293	1	y5
				640.377	1	y6
				1028.500	1	y10
PHB2_RAT=33291.91;Prohibitin-2 OS=Rattus norvegicus GN=Phb2 PE=1 SV=1	IGGVQQDTILAEGLHFR	618.667	3	700.370	2	y12
				764.399	2	y13
				870.955	2	y16
MYPR_RAT=30855.40;Myelin proteolipid protein OS=Rattus norvegicus GN=Plp1 PE=1 SV=2	LIETYFSK	500.771	2	544.276	1	y4
				645.324	1	y5
				774.366	1	y6
	GLSATVTGGQK	509.780	2	490.261	1	y5
				589.330	1	y6
				690.378	1	y7
SPTN1_RAT=285260.57;Spectrin alpha chain; non-erythrocytic 1 OS=Rattus norvegicus GN=Sptan1 PE=1 SV=2	ALINADELANDVAGAEALLDR	1077.555	2	716.393	1	y6
				844.452	1	y8
				915.489	1	y9
	DLIGVQNLLK	556.837	2	442.782	2	y8
				615.382	1	y5
				771.472	1	y7
TBA4A_RAT=50633.65;Tubulin alpha-4A chain OS=Rattus norvegicus GN=Tuba4a PE=2 SV=1	EIIDPVLDR	535.298	2	403.229	1	y3
				599.351	1	y5
				714.378	1	y6
	SIQFVDWC[Carboxyamidomethyl]PTGFK	792.879	2	549.303	1	y5
				709.333	1	y6
				1010.439	1	y8
LGUL_RAT=20977.40;Lactoylglutathione lyase OS=Rattus norvegicus GN=Glo1 PE=1 SV=3	VLGLTLLQK	492.826	2	501.339	1	y4
				602.387	1	y5
				772.492	1	y7
	DFLLQQTMLR	632.839	2	648.349	1	y5
				776.408	1	y6
				889.492	1	y7
SV2A_RAT=83406.24;Synaptic vesicle glycoprotein 2A OS=Rattus norvegicus GN=Sv2a PE=1 SV=2	GGLSDGEGPPGGR	578.273	2	483.267	1	y5
				540.288	1	y6
				726.352	1	y8
	FEEEEDDDDFPAPADGYYR	1140.448	2	558.267	1	y4
				841.383	1	y7
				1009.473	1	y9
VATB2_RAT=56856.98;V-type proton ATPase subunit B; brain isoform OS=Rattus norvegicus GN=Atp6v1b2 PE=1 SV=1	YAEIVHLTLPDGTK	778.919	2	517.261	1	y5
				731.393	1	y7
				981.536	1	y9
	TPVSEDMLGR	552.771	2	502.247	2	y9
				807.366	1	y7
				906.434	1	y8
VPP1_RAT=97063.43;V-type proton ATPase 116 kDa subunit a isoform 1 OS=Rattus norvegicus GN=Atp6v0a1 PE=2 SV=1	FTHGFQNIVDAYGIGTYR	1030.005	2	900.457	1	y8
				905.947	2	y16
				1114.552	1	y10
	LGFVAGVINR	523.311	2	402.245	1	y3

Target protein	Selected peptide	Precursor *m/z*	Charge state of precursor	Transitions *m/z*	Charge state of transition	Ion type of transition
				558. 335	1	y5
				629. 372	1	y6
NDUAA_RAT=40752.75;NADH dehydrogenase [ubiquinone] 1 alpha subcomplex subunit 10; mitochondrial OS=Rattus norvegicus GN=Ndufa10 PE=1 SV=1	VITVDGNIC[Carboxyamidomethyl]SGK	631.824	2	525.748	2	y10
				850.372	1	y8
				1050.488	1	y10
Internal Standard	Reduced and permethylated dextran composed of 4 glucose units	896.507	1	111.000	1	
				127.000	1	
				155.000	1	
