# Supplementary material for: The Effect of Chronic Methamphetamine Exposure on the Hippocampal and Olfactory Bulb Neuroproteomes of Rats
Source: PLoS One. 2016 Apr 15;11(4):e0151034. doi: 10.1371/journal.pone.0151034 (PMC4833297; doi:10.1371/journal.pone.0151034)
Supplement: S6 Table — (PDF) [file pone.0151034.s007.pdf]

Table S6. Target proteins and transitions of olfactory bulb proteome

Target protein	Selected peptide	Precursor *m/z*	Charge state of precursor	Transitions *m/z*	Charge state of transition	Ion type of transition
QCR1_RAT=53499.66;Cytochrome b-c1 complex subunit 1; mitochondrial OS=Rattus norvegicus GN=Uqcrc1 PE=1 SV=1	SLLTYGR	405.229	2	248.629	2	y4
				496.251	1	y4
				609.335	1	y5
	TDLTDYLSR	542.269	2	653.325	1	y5
				754.372	1	y6
				867.457	1	y7
K6PP_RAT=86634.81;6-phosphofructokinase type C OS=Rattus norvegicus GN=Pfkp PE=1 SV=2	DLLFKPVAELR	434.259	3	406.753	2	y7
				480.287	2	y8
				536.829	2	y9
	EIGWGDVGGWTGQGGSILGTK	1038.013	2	917.505	1	y10
				1018.552	1	y11
				1318.674	1	y14
GNAI1_RAT=40889.45;Guanine nucleotide-binding protein G(i) subunit alpha-1 OS=Rattus norvegicus GN=Gnai1 PE=1 SV=3	AVVYSNTIQSIIAIIR	587.68	3	401.287	1	y3
				472.324	1	y4
				585.408	1	y5
	EYQLNDSAAYYLNDLDR	1031.971	2	908.447	1	y7
				1071.51	1	y8
				1142.547	1	y9
ALDOC_RAT=39658.34;Fructose-bisphosphate aldolase C OS=Rattus norvegicus GN=Aldoc PE=1 SV=3	TPSALAILENANVLAR	826.97	2	757.431	1	y7
				886.474	1	y8
				999.558	1	y9
	LSQIGVENTEENRR	548.948	3	602.289	2	y10
				658.831	2	y11
				722.86	2	y12
SCRN1_RAT=46993.83;Secernin-1 OS=Rattus norvegicus GN=Scrn1 PE=1 SV=1	AQSPC[Carboxyamidomethyl]FGDDDPAK	704.296	2	604.748	2	y11
				1121.456	1	y10
				1208.488	1	y11
	SSPC[Carboxyamidomethyl]IHYFTGTPDPSR	911.415	2	977.468	1	y9
				1140.531	1	y10
				1277.59	1	y11
PP2BA_RAT=59290.53;Serine/threonine-protein phosphatase 2B catalytic subunit alpha isoform OS=Rattus norvegicus GN=Ppp3ca PE=1 SV=1	QTLQSATVEAIEADEAIK	958.994	2	775.383	1	y7
				1088.546	1	y10
				1288.663	1	y12
LIN7A_RAT=25962.57;Protein lin-7 homolog A OS=Rattus norvegicus GN=Lin7a PE=1 SV=2	LQESGEVPVHK	408.219	3	480.292	1	y4
				491.253	2	y9
				555.283	2	y10
H4_RAT=11360.38;Histone H4 OS=Rattus norvegicus GN=Hist1h4b PE=1 SV=2	VFLENVIR	495.293	2	501.314	1	y4
				630.356	1	y5
				743.441	1	y6
	TVTAMDVVYALK	655.855	2	494.297	1	y4
				938.501	1	y8
				1110.586	1	y10
KIF2A_RAT=80483.84;Kinesin-like protein KIF2A OS=Rattus norvegicus GN=Kif2a PE=2 SV=2	IDILTELR	486.79	2	518.293	1	y4
				631.377	1	y5
				744.461	1	y6
	FSLIDLAGNER	617.825	2	475.225	1	y4

Target protein	Selected peptide	Precursor *m/z*	Charge state of precursor	Transitions *m/z*	Charge state of transition	Ion type of transition
				546.263	1	y5
				774.374	1	y7
GRB2_RAT=25304.48;Growth factor receptor-bound protein 2 OS=Rattus norvegicus GN=Grb2 PE=1 SV=1	YFLWVVK	477.776	2	531.328	1	y4
				644.413	1	y5
				791.481	1	y6
	ESESAPGDFSLSVK	726.846	2	949.498	1	y9
				1020.535	1	y10
				1107.568	1	y11
CANB1_RAT=19401.62;Calcineurin subunit B type 1 OS=Rattus norvegicus GN=Ppp3r1 PE=1 SV=2	ISFEEFC[Carboxyamidomethyl]AVVGGLDIHK	640.989	3	787.387	2	y14
				860.922	2	y15
				904.438	2	y16
	VIDIFDTDGNGEVDFK	892.423	2	409.208	1	y3
				1196.506	1	y11
				1343.574	1	y12
PRDX6_RAT=24860.00;Peroxiredoxin-6 OS=Rattus norvegicus GN=Prdx6 PE=1 SV=3	FHDFLGDSWGILFSHPR	677.667	3	643.331	1	y5
				756.415	1	y6
				873.933	2	y15
	VVDSLQLTASNPVATPVDWK	1070.568	2	644.34	1	y5
				745.387	1	y6
				1498.789	1	y14
GNAI2_RAT=41043.27;Guanine nucleotide-binding protein G(i) subunit alpha-2 OS=Rattus norvegicus GN=Gnai2 PE=1 SV=3	IIHEDGYSEEEC[Carboxyamidomethyl]R	818.849	2	705.765	2	y11
				1029.394	1	y8
				1144.421	1	y9
	EYQLNDSAAYYLNDLER	1038.979	2	922.462	1	y7
				1085.526	1	y8
				1156.563	1	y9
PARK7_RAT=20189.51;Protein DJ-1 OS=Rattus norvegicus GN=Park7 PE=1 SV=1	VTVAGLAGK	408.253	2	445.276	1	y5
				516.314	1	y6
				615.382	1	y7
	DGLILTSR	437.753	2	476.282	1	y4
				589.366	1	y5
				702.45	1	y6
PLCB1_RAT=139112.55;1-phosphatidylinositol 4;5-bisphosphate phosphodiesterase beta-1 OS=Rattus norvegicus GN=Plcb1 PE=1 SV=1	TEDLIQSVLTEVEAQTIEELK	796.751	3	518.282	1	y4
				751.901	2	y13
				763.069	3	y20
	EADPGETSSEAPSETR	831.858	2	589.294	1	y5
				660.331	1	y6
				1064.485	1	y10
CNRP1_RAT=18817.66;CB1 cannabinoid receptor-interacting protein 1 OS=Rattus norvegicus GN=Cnrip1 PE=1 SV=1	IKPTTLQVENISIGGVLVPLELK	821.159	3	599.376	1	y5
				698.444	1	y6
				1024.64	1	y10
	VVYTGIYDTEGVAPTK	856.941	2	416.25	1	y4
				917.457	1	y9
				1080.52	1	y10
DPYL5_RAT=62071.43;Dihydropyrimidinase-related protein 5 OS=Rattus norvegicus GN=Dpysl5 PE=1	FVAVTSSNAAK	547.796	2	577.294	1	y6
				678.341	1	y7

Target protein	Selected peptide	Precursor *m/z*	Charge state of precursor	Transitions *m/z*	Charge state of transition	Ion type of transition
SV=1				848.447	1	y9
	C[Carboxyamidomethyl]HGVPLVTISR	413.561	3	476.282	1	y4
				575.351	1	y5
				688.435	1	y6
KCRB_RAT=42983.39;Creatine kinase B-type OS=Rattus norvegicus GN=Ckb PE=1 SV=2	DLFDPIIEDR	616.812	2	742.409	1	y6
				857.436	1	y7
				1004.504	1	y8
	FC[Carboxyamidomethyl]TGLTQIETLFK	779.403	2	979.545	1	y8
				1149.651	1	y10
				1250.698	1	y11
PHB_RAT=29858.92;Prohibitin OS=Rattus norvegicus GN=Phb PE=1 SV=1	IYTSIGEDYDER	730.831	2	826.321	1	y6
				883.342	1	y7
				1184.506	1	y10
	EFTEAVEAK	512.253	2	446.26	1	y4
				517.298	1	y5
				747.388	1	y7
PGAM2_RAT=28907.88;Phosphoglycerate mutase 2 OS=Rattus norvegicus GN=Pgam2 PE=2 SV=2	HYGGLTGLNK	530.283	2	702.414	1	y7
				759.435	1	y8
				922.499	1	y9
COX5A_RAT=16347.40;Cytochrome c oxidase subunit 5A; mitochondrial OS=Rattus norvegicus GN=Cox5a PE=1 SV=1	LNDFASAVR	496.762	2	432.256	1	y4
				503.293	1	y5
				765.388	1	y7
ES1_RAT=28496.98;ES1 protein homolog; mitochondrial OS=Rattus norvegicus PE=1 SV=2	ITNLAQLSAANHDAAIFPGGFGAAK	819.096	3	704.372	1	y8
				851.441	1	y9
				964.525	1	y10
FETUA_RAT=38756.51;Alpha-2-HS-glycoprotein OS=Rattus norvegicus GN=Ahsg PE=1 SV=2	LGGEEVSVAC[Carboxyamidomethyl]K	574.784	2	564.281	1	y5
				663.349	1	y6
				792.392	1	y7
	HAFSPVASVESASGEVLHSPK	712.695	3	866.473	1	y8
				953.505	1	y9
				964.491	2	y19
HDHD2_RAT=28932.17;Haloacid dehalogenase-like hydrolase domain-containing protein 2 OS=Rattus norvegicus GN=Hdhd2 PE=2 SV=1	TFFLEALR	498.779	2	488.282	1	y4
				601.366	1	y5
				748.435	1	y6
	LLLDGAPLIAIHK	458.622	3	517.803	2	y10
				574.345	2	y11
				791.513	1	y7
ANXA3_RAT=36568.67;Annexin A3 OS=Rattus norvegicus GN=Anxa3 PE=1 SV=4	SEIDLLDIR	537.295	2	403.229	1	y3
				516.314	1	y4
				857.509	1	y7
	GELSGHFEDLLLAVVR	585.653	3	444.292	1	y4
				557.376	1	y5
				656.374	2	y11
Internal Standard	Reduced and permethylated dextran composed of 4 glucose units	896.507	1	111	1	
				127	1	
				155	1	
