# Supplementary material for: The Effect of Chronic Methamphetamine Exposure on the Hippocampal and Olfactory Bulb Neuroproteomes of Rats
Source: PLoS One. 2016 Apr 15;11(4):e0151034. doi: 10.1371/journal.pone.0151034 (PMC4833297; doi:10.1371/journal.pone.0151034)
Supplement: S7 Table — (PDF) [file pone.0151034.s008.pdf]

Table S7. Table of Annotations of the interaction relations showing PubMed reference indicating the relationship and regulation type

Relation	Type	Sentence	MedLine Reference
GNAI1 --+> chemotaxis	Regulation	Little is known about the GTPase activity of the Galphai proteins involved in adhesion and chemotaxis, or the significance of their regulation to these responses.	9774420:3
GNAI1 --+> chemotaxis	Regulation	Conditioned media from bone marrow stroma induced receptor activation and chemotaxis that was sensitive to G alpha i and anti-receptor antibody inhibition.	12842911:4
GNAI1 --+> chemotaxis	Regulation	Because activation of any G protein presumably releases free Gbetagamma, we tested the hypothesis that chemotaxis also requires activated alpha subunits (Galphai) of Gi proteins.	9915816:2
GNAI1 --+> chemotaxis	Regulation	MIF triggered G(alphai)- and integrin-dependent arrest and chemotaxis of monocytes and T cells, rapid integrin activation and calcium influx through CXCR2 or CXCR4.	17435771:2
GNAI1 --+> chemotaxis	Regulation	We conclude that chemotaxis is dependent on activation of Galphai and the release of Gbetagamma dimers, and that Galphai-coupled receptors not traditionally associated with chemotaxis can mediate directed migration when they are expressed in hematopoietic cells.	9405641:5
GNAI1 --+> chemotaxis	Regulation	Both G alpha i/o proteins and phospholipase C are involved in histamine-induced calcium mobilization and chemotaxis in mast cells, because these responses were completely inhibited by pertussis toxin and phospholipase C inhibitor 1-[6-[[17 beta-3-methoxyestra-1,3,5 (10)-trien-17-yl]amino]hexyl]-1H-pyrrole-2,5-dione (U73122).	12626656:7
GNAI1 --+> chemotaxis	Regulation	PAFR-Gai3, PAFR-Gaq, and PAFR mediated chemotaxis.	16920964:1177
GNAI1 --+> chemotaxis	Regulation	However, it seems likely that ZAP-70 and Gai contribute differently to CXCR3-mediated T-cell chemotaxis.	17250586:1488
GNAI1 --+> chemotaxis	Regulation	In fact, CCR6-mediated chemotaxis of immune cells is potently inhibited upon blockade of Gai with pertussis toxin (33, 36).	19233848:1205
GNAI1 --+> chemotaxis	Regulation	It has been demonstrated recently that chemokine receptor-mediated chemotaxis is triggered by the ß? subunit of Gai (Neptune and Bourne, 1997).	10037796:1239
GNAI1 --+> chemotaxis	Regulation	In vitro, granulocytes display strong, Galphai-dependent chemotaxis to CXCL12 (reference 43 and unpublished data).	14707114:1296
GNAI1 --+> chemotaxis	Regulation	CXCR4 neutralization, Gai, and phosphatidylinositol 3-kinase inhibition significantly diminished CXCL12-stimulated chemotaxis.	15358596:1194
GNAI1 --+> chemotaxis	Regulation	For example, in BLT1-expressing RBL cells LTB4-induced chemotaxis, but not phosphatidylinositol hydrolysis and calcium mobilization, depended on Gai proteins (33).	17911632:1253
GNAI1 --+> chemotaxis	Regulation	Recently, it was demonstrated (45) that chemotaxis of T lymphocytes induced by CC chemokines is dependent on activation of Gai and the release of Gß? dimers and that Gai-coupled receptors not traditionally associated with chemotaxis can mediate directed migration when they are expressed in hemopoietic cells.	10201960:1250
GNAI1 --+> chemotaxis	Regulation	CHO-S1P2 cells stably expressing either Gai2, Gaq, Ga12, Ga13, or an empty vector were subjected to Western blot analysis using respective, specific anti-Ga antibodies described in Materials and Methods. (B and C) Overexpression of Gai markedly attenuates AlF4-- and sphingosine-1-phosphate-induced inhibition of IGF I-directed chemotaxis, but overexpression of Ga12 or Ga13 enhances such inhibition.	12588974:1241
GNAI1 ---> cell migration	Regulation	The complexity of this process is illustrated by the finding that Gai itself does not seem to be required for cell migration.	15383458:1069

Relation	Type	Sentence	MedLine Reference
GNAI1 ---> cell migration	Regulation	We conclude that both Gai and GIV are required for cell migration and mitosis, however, activation of Gai by GIV biases the cells to migrate.	20462955:1375
GNAI1 ---> cell migration	Regulation	Melanoma cell migration through an endothelial cell monolayer was dependent on one or more Gai signaling events.	18922934:1221
GNAI1 ---> cell migration	Regulation	Together, these data show that the phosphatidylinositol 3-kinase and ERK1/2 signaling pathways are key participants in Gai-CXCR4-directed epithelial cell migration.	15358596:1313
GNAI1 ---> cell migration	Regulation	The results presented here thus suggest that Gai proteins might also provide a converging signal for the cooperative modulation of hematopoietic stem cell migration by CXCL12 and extracellular uridine triphosphate.	17008551:1280
GNAI1 ---> cell migration	Regulation	pertussis toxin pretreatment stimulated cell migration of OGR1-PC3 cells but had no effect on vector-PC3 cells, suggesting that activating Gai proteins, which are sensitive to pertussis toxin, are involved in OGR1-induced inhibition of cell migration.	17728215:1366
GNAI1 ---> cell migration	Regulation	Activated Gai proteins are required for TF-1 cell migration, whereas phosphatidylinositol-3 kinase seem to be dispensable. (A) Proportion of TF-1 cells in spheroid cocultures with murine M2-10B4 stroma cell spheroids incubated with the specific inhibitors pertussis toxin (100 ng/ml) and LY294002 (10 μM) for 12 h, beginning with the initiation of cocultivation (n=4). (B) Serum components or M2-10B4-derived cytokines do not compensate for the inhibitory effect of LY294002 on the phosphatidylinositol-3 kinase pathway.	12377954:1169
calcineurin --+> glycolysis	Regulation	In mouse skeletal muscles, calcineurin activation enhanced lipid oxidation and attenuated glycolysis (21), increased the proportion of type I muscle fibers (6, 54), and reduced fatigability of fast-twitch tibialis anterior muscles (51, 52).	18199592:1081
SPTAN1 --+> apoptosis	Regulation	Moreover, we found that alpha-fodrin autoantigen induced Th1 immune responses and accelerated disturbance of Fas-mediated T cell apoptosis in aged Sjogren's syndrome model mice.	15593201:9
SPTAN1 --+> apoptosis	Regulation	Apoptosis in HepG2 cells mediated by immature plums was associated with "death receptor signaling." Immature plum extracts significantly increased the activation of caspase-8, -10, and -3 and expression of the caspase-3 target proteins alpha-fodrin (induces membrane blebbing and cell shrinkage), poly(ADP-ribose) polymerase (a nuclear enzyme that is involved in DNA repair following DNA nicks), and DNA fragmentation factor (induces apoptotic DNA fragmentation).	19627199:3
SPTAN1 --+> apoptosis	Regulation	Having demonstrated that a-fodrin proteolysis is an early event in apoptosis, we next wanted to determine the sensitivity of Fas-induced fodrin cleavage to a panel of protease inhibitors.	8940132:1108
SPTAN1 --+> apoptosis	Regulation	Taken together, it is tempting to speculate that ischemia/reperfusion induces calpain-mediated a-fodrin proteolysis that causes membrane disruption and necrosis during the early phase, and then caspase-mediated proteolysis of PARP and other apoptotic changes during the later phase.	11058563:1252
SPTAN1 --+> apoptosis	Regulation	Proteolytic cleavage of a-fodrin is implicated with apoptotic processes, as it is believed to be associated with membrane blebbing in apoptotic cells.12 Fragmentation of a-fodrin, an early event in apoptosis, is alternatively performed by calpain and caspase 3, which leads to generation of 150, 145 and 120 kDa cleavage products.13 However, a-fodrin cleavage may also proceed independent of calpain and caspase 3 activity, as shown during TGF-ß-induced apoptosis of a murine B-cell line.14 In addition, fragmentation of a-fodrin occurs during differentiation of nerve cells15 and lens fibres16, 17 as well as during myoblast fusion.18 Here, we show localization of a-fodrin in human placental tissues and address the issue of spectrin remodelling during intercellular trophoblast fusion.	19798107:1050

Relation	Type	Sentence	MedLine Reference
SPTAN1 --+> apoptosis	Regulation	Subsequently, the activation of caspase-3 downstream target proteins, poly (ADP-ribose) polymerase (PARP) and ?-fodrin could initiate apoptosis.	17635674:1287
GNAI1 ---> cell differentiation	Regulation	This suggests that the Cb2-mediated differentiation block requires interaction of G(alphai) proteins with other currently unknown motifs.	15039279:7
GNAI1 ---> cell differentiation	Regulation	We have previously shown, using the constitutively active Gai2 mutant (Gi2-Q205L), that the stimulatory effect of sphingosine 1-phosphate on MT1-MMP-dependent endothelial cell migration and morphogenic differentiation involves G protein ai subunits.	17541067:1219
GNAI1 ---> cell differentiation	Regulation	Recently Jorda et al41 have observed that Cb2, another Gai-G-protein-coupled receptor and a frequent proviral target in Cas-Br-M-MuLV-induced myeloid leukemias, produces an arrest in myeloid differentiation in the 32D system, suggesting a more general role of Gai-G-protein-coupled receptors in leukemogenesis.	15054042:1260
SPTAN1 --+> Ischemia	Regulation	Figure 4A shows that the 150- and 145-kDa fragments of a-fodrin increased significantly with time of reperfusion for 0.5, 6, and 24 h after 1 h of ischemia compared with the control (146.7% ± 13.4%, 213.9% ± 31.8%, and 323.8% ± 72.0%, respectively; Fig. 4A). m-Calpain was localized predominantly in the cytosolic fraction as an inactive proform (80 kDa) and underwent limited proteolysis with time of reperfusion following ischemia (37.3% ± 7.2% and 21.0% ± 5.6%, respectively; Fig. 4B).	11058563:1152
RALA ---> exocytosis	Regulation	Our results indicate that the interaction between RalA and the exocyst complex (containing Sec5) is essential for GTP-dependent exocytosis.	14978027:8
RALA ---> exocytosis	Regulation	Conversely, expression of the constitutively inactive GDP-bound RalA (G26A) or silencing of the RalA gene by RNA interference led to a strong impairment of the exocytotic response.	15980073:5
RALA ---> exocytosis	Regulation	Downregulation of RalA results in a limited level of reductions in GTP-dependent exocytosis.	17202486:1176
RALA ---> exocytosis	Regulation	These results suggest that RalA binding to the exocyst is required for the efficient exocytosis of insulin granules.	18426794:1219
RALA ---> exocytosis	Regulation	These previous results suggest that exocytosis of Weibel-Palade bodies requires the activation of RalA.	18417737:1119
RALA ---> exocytosis	Regulation	The fact that active RalA, which enhances the membrane delivery of newly synthesized proteins, localizes to recycling endosomes, a compartment also known to be involved in endocytosis, highlights the idea that RalA and its targets may function at the junction of exocytosis and endocytosis regulation.	15199131:1302
RALA --+> apoptosis	Regulation	Because apoptosis triggered by RalB small interfering RNA in cancer cells can be blocked by simultaneous transfection of RalA and RalB small interfering RNA (26), we sought to determine the effect on motility of both RalA and RalB depletion.	16103060:1158
RALA ---> cell growth	Regulation	These results suggest that merlin suppresses the RalGDS and RalA-mediated cell growth.	16007223:1150
RALA ---> cell growth	Regulation	In summary, we present the first demonstration that despite their significant sequence homology, RalA and RalB have nonoverlapping and opposing functions in cancer cell migration but overlapping functions in cell growth.	16103060:1053
GSTA1 ---| oxidative stress	Regulation	Enhanced expression of glutathione-S-transferase A1-1 protects against oxidative stress in human retinal pigment epithelial cells.	15652532:100
GSTA1 ---| oxidative stress	Regulation	These findings are in accord with previous studies, showing that GSTA4 expression is upregulated in response to oxidative stress induced by UVB (47,48) and that overexpression of GSTA1 protects against hydrogen peroxide-induced oxidative stress (49).	17984112:1255

Relation	Type	Sentence	MedLine Reference
GSTA1 ---| oxidative stress	Regulation	Genes containing a functional antioxidant response element(s) include those encoding hemeoxygenase 1 (HO-1), UDP-glucuronosyl transferase 1A (UGT1A), glutathione S-transferase A1/2, NAD(P)H:quinone reductase, and ?-glutamylcysteine synthetase, which play a crucial role in defense against oxidative stress or electrophilic chemicals (30).	17591699:1062
RALA ---> cell motility	Regulation	Although Ral proteins have previously been shown to be important for the motility of skeletal myoblasts and bladder cancer cells (27, 28), this is the first indication that RalA and RalB have nonoverlapping functions in cell motility.	16103060:1233
AHSG ---> apoptosis	Regulation	Bovine fetuin and human alpha(2)-HS glycoprotein significantly augmented the phagocytosis of apoptotic cells by human peripheral blood monocyte-derived macrophages, whereas the control proteins BSA, sialylated BSA and asialofetuin were ineffective.	12725640:3
AHSG ---> apoptosis	Regulation	Fetuin-A significantly inhibited apoptosis at all time points.	16093453:1186
AHSG ---> apoptosis	Regulation	Fetuin-alpha2-HS glycoprotein enhances phagocytosis of apoptotic cells and macropinocytosis by human macrophages.	15149366:1582
AHSG ---> apoptosis	Regulation	On the cellular level, fetuin-A accumulates in mineralization-competent matrix vesicles associated with vascular smooth muscle cells, thus attenuating apoptosis and dystrophic calcification (7).	16177000:1057
AHSG ---> apoptosis	Regulation	The prometastatic activity of fetuin-A is in sharp contrast to other studies demonstrating that bovine fetuin-A from fetal blood in the presence of zinc ions, can induce apoptotic cell death and abrogate tumor incidence in nude mice (12).	15695392:1296
AHSG ---> apoptosis	Regulation	Furthermore, fetuin-A may facilitate macrophages-mediated ingestion and elimination of apoptotic neutrophils [53], [54], thereby preventing secondary necrosis and passive leakage of injurious molecules (e.g., proteases, reactive oxygen species, and HMGB1) [55].	21347455:1235
AHSG ---> apoptosis	Regulation	Assuming that the continued buildup of calcified debris in the absence of fetuin-A may cause apoptosis in macrophages as it does in smooth muscle cells,26 it is tempting to speculate that calcification greatly enhances the vicious cycle of phagocytosis, apoptosis, etc. so well established in atherosclerosis-promoting macrophages laden with oxidized lipids.27 Intimal calcification patterns characterize older patients with chronic kidney disease,28 whereas younger patients typically exhibit calcifications of the vascular media.29 Our murine observations suggest that fetuin-A may help to prevent intima calcification but has little influence on media calcification and, as an alternative explanation, that only the intima, not the media, is damaged in this murine model and that such damage is a prerequisite for calcification.	19389852:1165
RALA ---> cell migration	Regulation	This phosphorylation potentiates RalA activation, anchorage-independent growth, and collagen I-induced cell migration (12).	17606711:1246
RALA ---> cell migration	Regulation	These results suggest that RalA and RalB have different roles in cell migration and that they may in fact act as antagonists with regard to this phenotype.	16103060:1051
RALA ---> cell migration	Regulation	During embryogenesis, the Ras-like GTPases RAP-1 and RAL-1 act in concert to orchestrate hypodermal cell migration and sorting.	17989692:1090
PSAP --+> neurite outgrowth	Regulation	In addition, prosaposin promotes neurite outgrowth in vitro via sequences in saposin C.	8636113:1
PSAP --+> neurite outgrowth	Regulation	Dose-response curves demonstrated that nanomolar concentrations of prosaposin and saposin C stimulated neurite outgrowth and increased ChAT activity.	7937812:3
PSAP --+> neurite outgrowth	Regulation	Extracellularly, intact prosaposin has ex vivo or in vivo functions as a neurite outgrowth or nerve regeneration factor, respectively (23,24).	17353235:1054

Relation	Type	Sentence	MedLine Reference
PSAP --+> neurite outgrowth	Regulation	Extracellularly, the intact prosaposin precursor functions ex vivo or in vivo as a neurite-outgrowth factor or nerve-regeneration factor respectively [11-14].	11085950:1034
PSAP --+> neurite outgrowth	Regulation	In each of these cells prosaposin stimulated neurite outgrowth and prevented cell death.	9895286:1054
PSAP --+> neurite outgrowth	Regulation	In each of these cells, prosaposin or prosaptides stimulate neurite outgrowth in nanomolar concentrations and prevent cell death.	9114068:1043
PSAP --+> neurite outgrowth	Regulation	When placed in the media surrounding neuroblastoma cells, prosaposin facilitates neurite outgrowth (6-8), and prosaposin facilitates in vivoregeneration of the sciatic nerve following injury (9).	9582364:1034
PSAP --+> neurite outgrowth	Regulation	Prosaposin and Prosaptide peptides have been shown to stimulate neurite outgrowth and choline acetyl transferase activity in vitro (O'Brien et al., 1995; Kotani et al., 1996b; Qi et al., 1996, 1999) and to prevent neuronal cell death induced by serum deprivation (O'Brien et al., 1995; Kotani et al., 1996b).	10773009:1036
PSAP --+> neurite outgrowth	Regulation	A significant portion of prosaposin is glycosylated, leading to a 70-kDa secreted form that is found in several extracellular fluids, such as cerebrospinal fluid, maternal milk, seminal plasma, and pancreatic secretions,24 25 26 27 28 and in the human29 and rat30 brain, where it is predominantly found in neurons.25 This secreted form can act as a neurotrophic, neuroprotective, reparative, and myelinotrophic factor.31 32 33 34 35 36 37 Prosaposin stimulates neurite outgrowth and prevents programmed cell death of a variety of neuronal cells.31 38 39 Moreover, prosaposin can protect neurons against ischemic damage.40 41 Direct application of prosaposin to transected sciatic nerves promotes nerve regeneration and/or prevents retrograde neuronal peripheral cell death after injury.32 These data suggest that prosaposin is an endogenous modulator of neuronal sprouting and regeneration.	15111580:1071
GNAI1 ---> cell growth	Regulation	LLC-PK1 cell growth is repressed by WT1 inhibition of G-protein alpha i-2 protooncogene transcription.	8530517:100
GNAI1 ---> cell growth	Regulation	Thus, Dehydroepiandrosterone induction of DU145 cell growth is also temporal, in parallel with c-Myc and cyclin D1 expression, and requires functional ERs and Gai/o subunits.	20176724:1206
BASP1 --+> apoptosis	Regulation	Overexpression of BASP1 induced cell death with features of apoptosis; conversely, small interfering RNA -mediated knockdown of BASP1 protected tubular cells from apoptosis.	20110383:6
PLP1 ---> apoptosis	Regulation	Abnormal PLP is thought to impair protein trafficking and to induce apoptosis in oligodendroglia.	12230321:5
PLP1 ---> apoptosis	Regulation	The regulation of apoptosis by PLP gene expression occurs independently of myelination, indicating that the PLP gene has multiple primary functions.	15662843:7
PLP1 ---> apoptosis	Regulation	The myelin proteolipid protein gene modulates apoptosis in neural and non-neural tissues.	15375385:100
PLP1 ---> apoptosis	Regulation	Disrupted proteolipid protein trafficking results in oligodendrocyte apoptosis in an animal model of Pelizaeus-Merzbacher disease.	9472043:100
PLP1 ---> apoptosis	Regulation	Disrupted proteolipid protein trafficking results in oligodendrocyte apoptosis in an animal model of Pelizaeus-Merzbacher disease.	15601821:1370
BASP1 ---> neuronal plasticity	Regulation	Brain-specific protein kinase C substrate, CAP-23/NAP-22, which is involved in the synaptogenesis and neuronal plasticity, binds calmodulin, but the protein lacks any canonical calmodulin-binding domain.	10207003:2
GNAI2 ---> cell differentiation	Regulation	Overexpression of wild-type Galphai2 or its Q205L constitutively activated mutant can induce differentiation in these 3T3-LI cells (3).	10807916:1043

Relation	Type	Sentence	MedLine Reference
GNAI2 ---> cell differentiation	Regulation	These results indicate that suppression of Galphai2 expression is required for all-trans-retinoic acid-induced F9 differentiation.	9857033:1267
GNAI2 ---> cell differentiation	Regulation	In contrast, retinoic acid-induced differentiation of F9 embryonal teratocarcinoma cells to the primitive endoderm is inhibited by expression of Gai2 (7).	15640523:1063
GNAI2 ---> cell differentiation	Regulation	Gai2 and Gas, like insulin, have been shown to be intimately involved in differentiation of 3T3-L1 adipocytes (14,29, 51-53) and stem cells (54, 55, 55-58).	11500506:1290
GNAI2 ---> cell differentiation	Regulation	The downregulation of Galphai2 by retinoic acid in F9 teratocarcinoma stem cells was shown to regulate their differentiation into primitive endoderm (28).	12324654:1307
GNAI2 ---> cell differentiation	Regulation	Sodium butyrate-induced erythroblastic differentiation of K562 cells requires the presence of Gai2, since pertussis toxin or an antisense oligonucleotide to a portion of the Gai2 gene blocks the sodium butyrate-induced effect (7).	11337508:1038
GNAI2 ---> cell differentiation	Regulation	A loss of function in the absence of MHC molecules suggests that Gai2 deficiency affects differentiation of CD4 and CD8 single-positive thymocytes only after the TCRs have made contact with peptides presented by either MHC class I or MHC class II molecule.	15684040:1209
PLP1 ---> endocytosis	Regulation	Colocalization of endocytosed PLP and myelin-associated glycoprotein increases over 10 and 30 minutes of endocytosis (yellow dots).	18303048:1125
PLP1 ---> endocytosis	Regulation	PLP, which forms a signaling complex with integrins in oligodendrocytes, may directly participate in the regulation of its own endocytosis by binding to the extracellular matrix (Gudz et al., 2002[Go], 2006[Go]).	17392472:1326
GNAI2 ---> chemotaxis	Regulation	Instead, we predicted that T cell chemotaxis to these ligands would require Gai2.	17938235:1166
GNAI2 ---> chemotaxis	Regulation	In fact, CXCL12-provoked chemotaxis was diminished in a lack of either Gai2 or Gai3 (Fig. 1F).	17289675:1169
GNAI2 ---> chemotaxis	Regulation	We observed that Gai2, Gao, Ga13, Gas, and Gaq/11 mediate natural killer cell chemotaxis induced by sphingosine 1-phosphate (Kveberg et al., 2002[Go]).	16109839:1300
NFASC ---> cell adhesion	Regulation	Phosphorylation or deletion of just the tyrosine residue in this sequence abolished ankyrin binding and significantly reduced neurofascin-mediated cell adhesion.	11997395:1044
NFASC ---> cell adhesion	Regulation	Finally, the binding of ankyrin G to the L1-family member neurofascin promotes neurofascin-mediated cell adhesion (Tuvia et al., 1997[Go]).	16597699:1060
NFASC ---> cell adhesion	Regulation	Furthermore, inhibition of the ankyrin-neurofascin interaction, either by deleting or phosphorylating the critical tyrosine residue, had an inhibitory effect on neurofascin-mediated cell adhesion (Tuvia et al., 1997).	9660878:1054
PLP1 ---> cell differentiation	Regulation	Proteolipid protein (PLP) has been postulated to play a critical role in the early differentiation of oligodendrocytes in addition to its known role as a structural component of myelin.	9045733:0
PLP1 ---> cell differentiation	Regulation	Mutations within the gene for myelin proteolipid protein (PLP), a major myelin structural protein, result in abnormal glial differentiation, suggesting that the PLP gene products play some other functional roles.	1374119:0
PLP1 ---> cell differentiation	Regulation	Oligodendrocyte death and other early jimpy abnormalities may be due to the presence of abnormal PLP message which may interfere with glial differentiation.	1705211:8

Relation	Type	Sentence	MedLine Reference
PLP1 ---> cell differentiation	Regulation	The demonstration of transcription of the PLP gene, long before the beginning of the myelination process, suggests that in addition to a structural function in myelin compaction, some of the products of the PLP gene (DM-20) may have a role during the compartmentalization and differentiation of the neural tube.	1737990:5
PLP1 ---> cell differentiation	Regulation	Among these are genes that are expressed only in mature myelin-producing oligodendrocytes, such as MAG, proteolipid protein 1 (PLP1), and MBP, but also genes involved in the differentiation and maturation of oligodendrocytes, like SOX10 and transferrin (17).	16641098:1055
PLP1 ---> cell differentiation	Regulation	An underlying theme of many earlier studies has been the suggestion that dysmyelination in the central nervous system of these mutants stems from an absence of functional DM-20/PLP, which arrests the differentiation of virtually all oligodendrocyte progenitors at an immature stage, resulting in the accumulation of premyelinating cells that subsequently die (Webster and Sternberger, 1980; Skoff and Knapp, 1992; Nadon and Duncan, 1995).	9472043:1219
RALA ---> cell differentiation	Regulation	These data functionally support the hypothesis that RGL1 and RGL2 are exchange factors for RAL. (iii) The activation of RAL required for the differentiation of sensory bristles might be independent of RAS1 signaling.	12529414:1258
RALA ---> cell differentiation	Regulation	Recent reports (20, 21) suggest that two other members of the Ras-like small GTPase family, namely RalA and RalB, possess pivotal roles in the control of cell proliferation, migration, differentiation, cytoskeletal organization, vesicular transport, and receptor endocytosis.	12215457:1039
RALA ---> cell differentiation	Regulation	Recent reports (20, 21) suggest that two other members of the Ras-like small GTPase family, namely RalA and RalB, possess pivotal roles in the control of cell proliferation, migration, differentiation, cytoskeletal organization, vesicular transport, and receptor endocytosis.	12218045:1039
RALA ---> endocytosis	Regulation	Recent reports (20, 21) suggest that two other members of the Ras-like small GTPase family, namely RalA and RalB, possess pivotal roles in the control of cell proliferation, migration, differentiation, cytoskeletal organization, vesicular transport, and receptor endocytosis.	12215457:1039
RALA ---> endocytosis	Regulation	Recent reports (20, 21) suggest that two other members of the Ras-like small GTPase family, namely RalA and RalB, possess pivotal roles in the control of cell proliferation, migration, differentiation, cytoskeletal organization, vesicular transport, and receptor endocytosis.	12218045:1039
RALA ---> endocytosis	Regulation	The fact that active RalA, which enhances the membrane delivery of newly synthesized proteins, localizes to recycling endosomes, a compartment also known to be involved in endocytosis, highlights the idea that RalA and its targets may function at the junction of exocytosis and endocytosis regulation.	15199131:1302
GNAI1 ---> apoptosis	Regulation	Using a neonatal rat myocyte model, it was shown that ß2AR/Gai-mediated protection from apoptosis occurs through phosphatidylinositol 3-kinase and Akt/protein kinase B pathways (8).	12065589:1246
GNAI1 ---> apoptosis	Regulation	The inhibition of palmitic acid- or lysophosphatidylcholine-induced Chang cell apoptosis by pertussis toxin or a dominant-negative Gai mutant (18) suggests that certain G-protein-coupled receptor/Gai is involved in apoptosis by endogenous or exogenous lysophosphatidylcholine (55).	17951222:1327
GNAI1 ---> apoptosis	Regulation	In addition, the inhibition of cardiac Gai-2 increased infarct size and apoptosis in transgenic mice expressing a Gai-2 inhibitor peptide, when the mice were subjected to ischemia/reperfusion indicating an important role of this isoform in	19719783:1376

Relation	Type	Sentence	MedLine Reference
		cardioprotection.	
SV2A ---> exocytosis	Regulation	These findings demonstrate that SV2A is an essential protein and implicate it in the control of exocytosis.	10611374:9
SV2A ---> exocytosis	Regulation	Alternatively, Synaptic vesicle protein 2 could modulate exocytosis by interacting with synaptotagmin I (Schivell et al., 1996[Go]; Lazzell et al., 2004[Go]) or act as a scaffold protein that regulates vesicle shape (Janz et al., 1998[Go]).	16306227:1066
BASP1 --+> neurite outgrowth	Regulation	BASP1 overexpression stimulated neurite outgrowth in both cell types.	18438920:2
BASP1 --+> neurite outgrowth	Regulation	BASP1 is also important for neurite outgrowth and regulates nerve sprouting.	17938642:1179
BASP1 --+> neurite outgrowth	Regulation	BASP1 is known to control neurite outgrowth and in the same way may control the development of the neurite-like structures observed in germinal centre B cells.	18172440:1043
BASP1 --+> neurite outgrowth	Regulation	Overall, according to this model, the expression of proteins such as GAP43, MARCKS, or CAP23 would promote and regulate cell-surface dynamics, phagocytosis, cell attachment, and regulated morphogenic processes such as neurite outgrowth (Fig 10).	10871285:1350
BASP1 --+> regeneration	Regulation	Increased expression of growth-associated proteins (e.g., GAP-43 and CAP23) and inactivation of the Rho signaling pathway also promote regeneration (33-35).	16275900:1203
BASP1 --+> regeneration	Regulation	CAP-23/NAP-22 binds to calmodulin in a myristoylation-dependent manner (Hayashi et al., 2000[Go]; Takasaki et al., 1999[Go]), and stimulates neuronal competence for axon regeneration (Bomze et al., 2001[Go]).	17046994:1221
GNAI2 ---> cell migration	Regulation	Altered GTP?S Incorporation in the Absence of Gai2 or Gai3-Our migration data show involvement of both Gai2 and Gai3 in CXCR3-mediated signaling, presumably with Gai2 activating downstream effectors to drive T cell migration.	17289675:1193
GNAI2 ---> cell migration	Regulation	Alternatively, Gai2 deficiency may impair chemorepulsion or cell migration away from a stimulus so that the duration of the interaction between the TCRs and peptide/MHC complexes would be extended on the cortical epithelial cells, where positive selection occurs.	15684040:1263
GNAI1 ---> cell motility	Regulation	In addition, genetic approaches have led to the conclusion that Gai was required for normal B cell motility within LNs (9).	17485513:1055
SV2A ---> endocytosis	Regulation	Thus, Synaptic Vesicle Protein 2 could influence the trafficking of proteins to synaptic vesicles by regulating clathrin-mediated endocytosis of vesicle proteins.	19381277:1305
PLP1 ---> cell motility	Regulation	PLP appeared to play an important role in neurotransmitter-induced stimulation of oligodendrocyte precursor cell motility.	16510724:1178
GNAI1 ---> neurite outgrowth	Regulation	We have previously shown that CB1 receptor-coupled Gai/o mediates neurite outgrowth through the activation of Rap1 (9).	16046413:1198
GNAI1 ---> neurite outgrowth	Regulation	This promotion of neurite outgrowth by Thy-1 is dependent on Gai and L- and N-type calcium channel activation (27) .	16770003:1074
PSAP ---| Ischemia	Regulation	These findings suggest that prosaposin possesses neurotrotrophic activity to protect hippocampal CA1 neurons from lethal ischemic damage.	7980569:4
PSAP ---| Ischemia	Regulation	The relative abundance of the exon 8-containing prosaposin isoform was recently shown to sharply decline following ischemia and stab wound in rat brain (19).	15743835:1299

Relation	Type	Sentence	MedLine Reference
PSAP ---| Ischemia	Regulation	A significant portion of prosaposin is glycosylated, leading to a 70-kDa secreted form that is found in several extracellular fluids, such as cerebrospinal fluid, maternal milk, seminal plasma, and pancreatic secretions,24 25 26 27 28 and in the human29 and rat30 brain, where it is predominantly found in neurons.25 This secreted form can act as a neurotrophic, neuroprotective, reparative, and myelinotrophic factor.31 32 33 34 35 36 37 Prosaposin stimulates neurite outgrowth and prevents programmed cell death of a variety of neuronal cells.31 38 39 Moreover, prosaposin can protect neurons against ischemic damage.40 41 Direct application of prosaposin to transected sciatic nerves promotes nerve regeneration and/or prevents retrograde neuronal peripheral cell death after injury.32 These data suggest that prosaposin is an endogenous modulator of neuronal sprouting and regeneration.	15111580:1071
AHSG --+> endocytosis	Regulation	The ability of alpha 2HS glycoprotein to promote the endocytosis of radiolabelled DNA and radiolabelled latex particles by mouse macrophages was investigated.	7439929:1
AHSG --+> endocytosis	Regulation	AHSG promotes endocytosis, possesses opsonic properties, and is a negative acute-phase protein.32,33 The upregulated expression of AHSG in uveitic sera could be connected to the role of AHSG as a modulator of immune responses and could demonstrate a reaction to the inflammatory process and therefore deserves closer investigation.	19696180:1207
SV2A ---> transmission of nerve impulse	Regulation	We found that synaptic vesicle protein 2 selectively enhances low-frequency neurotransmission by priming morphologically docked vesicles.	16436618:1
SV2A ---> transmission of nerve impulse	Regulation	Synaptic vesicle protein 2A (SV2A) has been identified as the binding site for the antiepileptic drug levetiracetam and is thought to decrease neuronal excitability.	20167814:0
SV2A ---> transmission of nerve impulse	Regulation	Synaptic vesicle protein 2A (SV2A), the binding site for the antiepileptic drug levetiracetam, has been shown to be involved in the control of neuronal excitability.	19220410:0
SV2A ---> transmission of nerve impulse	Regulation	Introduction of the amino terminus of SV2A or SV2C into cultured superior cervical ganglion neurons inhibited neurotransmission, whereas the amino terminus of SV2B did not.	15866046:6
SV2A ---> transmission of nerve impulse	Regulation	The results presented here demonstrate that SV2A is required for normal neurotransmission.	10611374:1282
SV2A ---> transmission of nerve impulse	Regulation	Both inhibitory (16) and excitatory (V. Lopantsev and S.M.B., unpublished results) neurotransmission is reduced in the absence of SV2A.	15210974:1208
SV2A ---> transmission of nerve impulse	Regulation	The synaptic vesicle protein Synaptic vesicle protein 2 is a membrane glycoprotein common to all synaptic vesicles and is essential for normal neurotransmission.	10747945:1256
SV2A ---> transmission of nerve impulse	Regulation	The decrease in the readily releasable pool size and the absence of changes in the release probability observed in SynI and SV2A knocked out neurons suggest that both SynI and SV2A physiologically sustain low-frequency neurotransmission by selectively enhancing priming of SVs and regulating readily releasable pool size.	18057210:1365
PLP1 --+> cell migration	Regulation	PLP contributed to oligodendrocyte precursor cell migration on fibronectin, because an antibody against an extracellular domain of PLP (PLP1) reduced cell migration.	16510724:1135
GNAI1 ---> exocytosis	Regulation	In contrast, inhibition of G alpha i-2 stimulated exocytosis and allowed cAMP to stimulate CFTR GCl in cells isolated from patients with cystic fibrosis .	7519398:4

Relation	Type	Sentence	MedLine Reference
GNAI1 ---> exocytosis	Regulation	The pertussis toxin-sensitive G-proteins Gai control exocytosis and release of preformed mediators, such as prostaglandins and leukotrienes.	12881705:1179
GNAI1 ---> exocytosis	Regulation	PAFR-Gai3 and PAFR-Gaq, however, mediated lower phosphoinositide hydrolysis and exocytosis relative to PAFR (Figs. 2 and 3).	16920964:1270
PSAP ---> regeneration	Regulation	The notion that prosaposin is likely involved in brain development and regeneration led us to explore its expression in stem/progenitor neural cells and its fate after cell differentiation.	18346466:0
PSAP ---> regeneration	Regulation	A significant portion of prosaposin is glycosylated, leading to a 70-kDa secreted form that is found in several extracellular fluids, such as cerebrospinal fluid, maternal milk, seminal plasma, and pancreatic secretions,24 25 26 27 28 and in the human29 and rat30 brain, where it is predominantly found in neurons.25 This secreted form can act as a neurotrophic, neuroprotective, reparative, and myelinotrophic factor.31 32 33 34 35 36 37 Prosaposin stimulates neurite outgrowth and prevents programmed cell death of a variety of neuronal cells.31 38 39 Moreover, prosaposin can protect neurons against ischemic damage.40 41 Direct application of prosaposin to transected sciatic nerves promotes nerve regeneration and/or prevents retrograde neuronal peripheral cell death after injury.32 These data suggest that prosaposin is an endogenous modulator of neuronal sprouting and regeneration.	15111580:1071
GNAI1 ---> transmission of nerve impulse	Regulation	Gai/o subunits modulate the level of cAMP by regulating adenylate cyclase activities at postsynaptic sites, and inhibition of neuronal excitability (Simonds, 1999[Go]; Billinton et al., 2001[Go]).	20071540:1047
PLP1 ---> oxidative stress	Regulation	TABLE 1 Effect of PLP-dependent enzyme inhibition and oxidative stress on the concentration of cytoplasmic glycine and serine1.	19244383:1143
GNAI2 --+> ROS generation	Regulation	Interestingly, overexpression of Gai2 increased both fMLF- and phorbol myristate acetate-induced O2- generation (Fig. 7A and B).	16782902:1241
PSAP ---> cell differentiation	Regulation	These findings suggest that prosaposin may be involved in the development and maintenance of the male reproductive organs, as well as, in cellular differentiation.	10864364:8
PSAP ---> cell differentiation	Regulation	In addition, pertussis toxin inhibited prosaptide-induced neurite outgrowth, as well as prosaptide-enhanced ganglioside concentrations in NS20Y cells, suggesting that prosaposin acted via a G protein-mediated pathway, affecting both ganglioside content and neuronal differentiation.	9832129:6
PSAP ---> cell differentiation	Regulation	Prosaposin is also an important factor in development, maintenance, and differentiation of male reproductive organs (40, 42).	15743835:1053
PSAP ---> cell differentiation	Regulation	Prosaposin is also thought to have specific effects on the development, maintenance, and differentiation of the male reproductive organs and may play a role in lysosomal residual body degradation in Sertoli cells.42 43 .	15111580:1072
PSAP ---> cell differentiation	Regulation	At least in rodents, PSAP is thought to have specific effects on the development, maintenance and differentiation of male reproductive organs and may also play a role in lysosomal residual body degradation in Sertoli cells (36).	11309366:1078
PSAP ---> cell differentiation	Regulation	Prosaposin and prosaptides appear to induce both differentiation of neuronal cells (2, 3, 8) and synthesis of gangliosides (19); the present study indicates that Schwann cells and oligodendrocytes respond similarly.	9114068:1148
PSAP ---> cell differentiation	Regulation	In a variety of neuro-glial derived cells, synthetic peptides encompassing a trophic sequence of saposin C and/or prosaposin have been found to induce growth, survival, and/or differentiation, or to prevent apoptotic cell-death in vitro and in vivo [11,18-20].	15548330:1186

Relation	Type	Sentence	MedLine Reference
UQCRC1 ---| ROS generation	Regulation	Expression of two genes whose products are involved in the production of Reactive oxygen species was decreased by MnTBAP, ubiquinol-cytochrome c reductase core protein 1, and P450 oxidoreductase (17-19).	14657380:1116
GNAI1 ---> endocytosis	Regulation	Gia1-mediated inhibition of endocytosis is partially reversed by C3 exoenzyme.	9560227:1139
AHSG --+> Inflammation	Regulation	Fetuin-A inhibits inflammation and has a protective effect against myocardial ischemia.	17702860:0
AHSG --+> Inflammation	Regulation	In the presence of inflammation, cardiovascular event-free survival was influenced by common variants in the AHSG gene.	21527649:9
AHSG --+> Inflammation	Regulation	Plasma concentrations of alpha2-HS glycoprotein decrease significantly following infection, inflammation and malignancy.	11922920:1
AHSG --+> Inflammation	Regulation	Fetuin-A, a protein almost exclusively secreted by the liver, induces insulin resistance and subclinical inflammation in rodents.	19029462:0
AHSG --+> Inflammation	Regulation	We provide novel evidence that the secreted liver protein fetuin-A induces low-grade inflammation and represses adiponectin production in animals and in humans.	18335040:10
AHSG --+> Inflammation	Regulation	In addition, fetuin-A induced low-grade inflammation (24), which is also associated with the metabolic syndrome and an atherogenic lipid profile (1, 5).	18728159:1187
AHSG --+> Inflammation	Regulation	As fetuin-A may have important functions in inflammation, such as limitation of cytokine production by macrophages32 and protection against TNF33, further studies are needed to investigate the role of fetuin-A in the inflamed end-stage renal disease patient.	15882283:1202
AHSG --+> Inflammation	Regulation	Although TNF-a and IL-6 are among the cytokines potentially capable of down-regulating hepatic fetuin-A expression it should also be noted that fetuin-A may have important functions in inflammation, such as limitation of cytokine production by macrophages and protection against TNF118.	15780075:1284
AHSG --+> Inflammation	Regulation	In a parallel study, we found that repetitive administration of fetuin-A (100 mg/kg) at 24, 48, and 72 h after the onset of peritoneal infection (induced by cecal ligation and puncture) promoted a long-lasting protection against lethal systemic inflammation at 14 day after cecal ligation and puncture (Wang et al, unpublished observations).	19953099:1160
GNAI2 --+> exocytosis	Regulation	Activation of betagamma subunits of G(i2) and G(i3) proteins by basic secretagogues induces exocytosis through phospholipase Cbeta and arachidonate release through phospholipase Cgamma in mast cells.	11673483:100
GNAI2 --+> exocytosis	Regulation	Chem. 271, 23458-23463 Medline Crossref 1st Citation . 22 Ferry, X., Eichwald, V., Daeffler, L. and Landry, Y. (2001) Activation of betagamma subunits of G(i2) and G(i3) proteins by basic secretagogues induces exocytosis through phospholipase Cbeta and arachidonate release through phospholipase Cgamma in mast cells.	17492941:1300
PSAP ---| apoptosis	Regulation	Prosaposin binding induces U937 cell death prevention, reducing both necrosis and apoptosis.	15242760:2
PSAP ---| apoptosis	Regulation	All these findings indicated that Fibrocystin/polyductin and prosaposin may play significant roles in regulation of cell proliferation and apoptosis.	20709014:12
PSAP ---| apoptosis	Regulation	Prosaposin may play a role in cerebellar development during programmed cell death of cerebellar neurons.	9748612:9
PSAP ---| apoptosis	Regulation	In addition, prosaptide TX14A, saposin C, or prosaposin decreased the growth-inhibitory effect, caspase-3/7 activity, and apoptotic cell death induced by etoposide.	15548330:7

Relation	Type	Sentence	MedLine Reference
PSAP ---| apoptosis	Regulation	We report that prosaposin treatment induced extracellular signal-regulated kinases and sphingosine kinase activity, increased DNA synthesis, and prevented cell apoptosis.	11156962:0
PSAP ---| apoptosis	Regulation	In addition, unprocessed prosaposin functions as a neurotrophic factor in the central and peripheral nervous systems by acting to prevent neuronal apoptosis, to elongate neurites and to facilitate myelination.	15927723:1
PSAP ---| apoptosis	Regulation	Histone-associated DNA fragmentation enzyme-linked immunosorbent assay, showed a 10- and 14-fold increase in apoptosis after 4 and 24 hr in low serum medium, respectively, that was reduced by prosaposin, TX14(A), or insulinlike growth factor-I.	10412024:4
PSAP ---| apoptosis	Regulation	Moreover, their precursor, prosaposin, plays a role in the field of apoptosis regulation.	15992358:1168
PSAP ---| apoptosis	Regulation	Prosaposin in the secretome of marrow stroma-derived neural progenitor cells protects neural cells from apoptotic death.	20050969:100
PSAP ---| apoptosis	Regulation	More recently, prosaposin and Prosaptide TX14(A) have been shown to prevent apoptosis of cerebellar granule cells (Tsuboi et al., 1998) and Schwann cells (Campana et al., 1999).	10773009:1037
PSAP ---| apoptosis	Regulation	For example, prosaposin is a natural component of milk and a key regulatory factor in the ceramide-S-1-P rheostat, which mediates its effects by promoting DNA synthesis and inhibiting apoptosis (40).	16293640:1219
PSAP ---| apoptosis	Regulation	In particular, CTGF, activin A, epithelin/granulin, and galectin-3 were reported to act as mitogens (36-39), whereas galectin-3 and prosaposin inhibit apoptosis (40, 41). p21 also induced intracellular proteins SOD2 and R-Ras with reported antiapoptotic activity (42, 43), as well as tissue transglutaminase and cathepsin B ascribed a proapoptotic function (27).	10760295:1138
PSAP ---| apoptosis	Regulation	A significant portion of prosaposin is glycosylated, leading to a 70-kDa secreted form that is found in several extracellular fluids, such as cerebrospinal fluid, maternal milk, seminal plasma, and pancreatic secretions,24 25 26 27 28 and in the human29 and rat30 brain, where it is predominantly found in neurons.25 This secreted form can act as a neurotrophic, neuroprotective, reparative, and myelinotrophic factor.31 32 33 34 35 36 37 Prosaposin stimulates neurite outgrowth and prevents programmed cell death of a variety of neuronal cells.31 38 39 Moreover, prosaposin can protect neurons against ischemic damage.40 41 Direct application of prosaposin to transected sciatic nerves promotes nerve regeneration and/or prevents retrograde neuronal peripheral cell death after injury.32 These data suggest that prosaposin is an endogenous modulator of neuronal sprouting and regeneration.	15111580:1071
PLP1 ---> Inflammation	Regulation	Further studies will be needed to plot a quantitative time course for PLP-induced inflammation and axonal pathology and assess its impact on the expression of the neurological deficit and spinal cord transmission.	14566007:1247
PLP1 ---> Inflammation	Regulation	Immunization with self-neuronal antigens, such as MBP, myelin-associated glycoprotein, proteolipid protein or myelin oligodendrocyte glycoprotein (3, 4), results in inflammation within the central nervous system primarily mediated by CD4+ Th1 cells (1, 2).	16415102:1058
SCRN1 ---> exocytosis	Regulation	We have used this as the basis of a bioassay to purify Secernin 1, a novel 50-kDa cytosolic protein that appears to be involved in the regulation of exocytosis from peritoneal mast cells.	12221138:3
SCRN1 ---> exocytosis	Regulation	Secernin 1 has dipeptidase activity and has been demonstrated to play a role in exocytosis; it has been shown to be overexpressed in certain types of cancer and has also been suggested as a potential neurotoxicologically relevant target.	20069063:6

Relation	Type	Sentence	MedLine Reference
NFASC ---| transmission of nerve impulse	Regulation	Genetic ablation of genes encoding the critical paranodal proteins Caspr, contactin , and the myelinating glia-specific isoform of Neurofascin (Nfasc(NF155)) results in the disruption of the paranodal axo-glial junctions, loss of ion channel segregation, and impaired nerve conduction, but the mechanisms regulating their interactions remain elusive.	20371806:1
NFASC ---| transmission of nerve impulse	Regulation	Thus, dispersion of neurofascin-186 away from the axon initial segment through loss of ankyrinG and ßIV spectrin might also disrupt GABAergic neurotransmission at this site.	19846712:1479
RALA ---> neurite outgrowth	Regulation	Hence we anticipated that the downregulation of RalA and RalB inhibits NGF-induced neurite outgrowth.	17202486:1387
ATP6V0A1 ---> apoptosis	Regulation	Morpholino knockdown of the atp6v0a1 subunit in zebrafish leads to deficiencies in microglial-mediated mediated neuronal degradation and clearance of apoptotic neurons within the zebrafish brain.24 Loss of atp6v0a1 function was not reported to lead to increased levels of apoptosis in the brain, however.	18836174:1233
ATP6V0A1 ---> apoptosis	Regulation	Morpholino knockdown of the atp6v0a1 subunit in zebrafish leads to deficiencies in microglial-mediated mediated neuronal degradation and clearance of apoptotic neurons within the zebrafish brain.24 Loss of atp6v0a1 function was not reported to lead to increased levels of apoptosis in the brain, however.	18836173:1233
GNAI2 ---> cell motility	Regulation	Rgs1 and Gnai2 regulate the entrance of B lymphocytes into lymph nodes and B cell motility within lymph node follicles.	15780991:100
GNAI2 ---> cell motility	Regulation	H. (2005) Rgs1 and Gnai2 regulate the entrance of B lymphocytes into lymph nodes and B cell motility within lymph node follicles.	19159344:1446
GSTA1 ---| apoptosis	Regulation	Human GSTA1-1 reduces c-Jun N-terminal kinase signalling and apoptosis in Caco-2 cells.	16836488:100
GSTA1 ---| apoptosis	Regulation	Human GSTA1-1 reduces c-Jun N-terminal kinase signalling and apoptosis in Caco-2 cells.	20596078:1236
calcium channel --+> voltage-gated Ca2+ channel	Regulation	Ca2+ channel antagonist drugs inhibit voltage-gated Ca2+ channels in many different cell types.	1321525:0
calcium channel --+> voltage-gated Ca2+ channel	Regulation	Rat brain synaptosomes are shown to contain functional voltage-sensitive Ca2+ channels that are inhibited by organic Ca2+ channel blockers.	2579220:0
calcium channel --+> voltage-gated Ca2+ channel	Regulation	Voltage-sensitive calcium channel activity was blocked by organic Ca2+ channel antagonists (nanomolar range) both before and after KCl treatment and also by divalent metal cations (micromolar range).	2452233:6
calcium channel --+> voltage-gated Ca2+ channel	Regulation	In contrast to those Voltage-sensitive calcium channels involved in neurotransmitter release, the Voltage-sensitive calcium channels described here appear to be blocked by organic calcium channel antagonists at very low concentrations.	6202853:11
calcium channel --+> voltage-gated Ca2+ channel	Regulation	Here we report a novel mechanism for G protein-mediated modulation of neuronal voltage-dependent calcium channels that involves the destabilization and subsequent removal of calcium channels from the plasma membrane.	16293615:1
calcium channel --+> voltage-gated Ca2+ channel	Regulation	The data suggest that hormonal stimulation of Na+/K+-ATPase activity interferes with activation of voltage-sensitive calcium channels by either membrane hyperpolarization or some unknown interaction between the sodium pump and calcium channels.	10650930:6

Relation	Type	Sentence	MedLine Reference
calcium channel --+> voltage-gated Ca2+ channel	Regulation	Because the voltage-dependent Ca2+ channel is inhibited by Ca2+ channel blockers, contractions elicited by high K+ are inhibited by this type of blocker.	9228665:1159
calcium channel --+> voltage-gated Ca2+ channel	Regulation	Nonselective Ca2+ channel blockers like La3+ block the Ca2+ influx produced by high-K+ stimulation by blocking voltage-dependent Ca2+ channels.	12968012:1190
calcium channel --+> voltage-gated Ca2+ channel	Regulation	Dissection of the calcium channel domains responsible for modulation of neuronal voltage-dependent calcium channels by G proteins.	10414293:100
calcium channel --+> voltage-gated Ca2+ channel	Regulation	Canti, Dissection of the calcium channel domains responsible for modulation of neuronal voltage-dependent calcium channels by G proteins, Ann.	10920007:1755
calcium channel --+> voltage-gated Ca2+ channel	Regulation	1,4-dihydropyridine Ca2+ channel blockers are commonly used at concentrations as high as 10 μM to block voltage-gated Ca2+ channel, although they are specific for this purpose only in the nanomolar range (Triggle, 2003).	14718582:1295
calcium channel --+> voltage-gated Ca2+ channel	Regulation	Calcium channel blockers inhibit calcium influx through membrane-bound voltage-dependent calcium channels, resulting in decreased intracellular calcium levels and vasodilation (83).	11772917:1320
calcium channel --+> voltage-gated Ca2+ channel	Regulation	Calcium channel blockers inhibit calcium influx through membrane-bound voltage-dependent calcium channels, resulting in decreased intracellular calcium levels and vasodilation (83).	11772914:1320
calcium channel --+> voltage-gated Ca2+ channel	Regulation	Admittedly, organic Ca2+ channel antagonists produce vasorelaxation predominantly by inhibiting the influx of Ca2+ into smooth muscle cells via the so-called voltage-gated Ca2+ channels [2].	14553819:1021
calcium channel --+> voltage-gated Ca2+ channel	Regulation	The latter involves a complex interplay between different Ca2+ channels: AVP promotes opening of L-type voltage-gated Ca2+ channels (38), and it can reciprocally regulate capacitative Ca2+ entry and a noncapacitative Ca2+ entry pathway (39, 40).	15632122:1081
calcium channel --+> voltage-gated Ca2+ channel	Regulation	calcium channel blockers inhibit the movement of calcium into cells by interfering with the action of the voltage-gated calcium channels.2 As calcium entry into cardiac myocytes is reduced, the result is negative inotropy, chronotropy, and dromotropy.	19224785:1118
calcium channel --+> voltage-gated Ca2+ channel	Regulation	Thus, Ca2+ influx via voltage-gated Ca2+ channels is mostly likely depressed during the action potential due to the concomitant decrease in the number of available Ca2+ channels following depolarization of the resting potential expected under these conditions.	14990678:1365
calcium channel --+> voltage-gated Ca2+ channel	Regulation	Traditionally, agonist-induced intracellular [Ca2+] ([Ca2+]i) elevation in airway smooth muscle was thought to depend on Ca2+ influx through the plasma membrane with the L-type voltage-gated Ca2+ channels being implicated as the main Ca2+ channels.	17170384:1065
calcium channel --+> voltage-gated Ca2+ channel	Regulation	The light peak of the electrooculogram, which is a hallmark diagnostic feature of Best vitelliform macular dystrophy, is apparently dependent on voltage-gated Ca2+ channels in mouse, because the light peak is reduced by the Ca2+ channel blocker nimodipine and is abolished in ß4 (Marmorstein et al., 2006[Go]) and CaVa1.3 knock-out (Wu et al., 2007[Go]) mice.	18509027:1302

Relation	Type	Sentence	MedLine Reference
calcium channel --+> voltage-gated Ca2+ channel	Regulation	High-K+ depolarization has been shown to increase [Ca2+]i by activating voltage-dependent Ca2+ channels, which are inhibited by Ca2+ channel blockers. 17ß-Estradiol (30 µmol/L) completely inhibited the high-K+-induced increase in [Ca2+]i and contraction to resting levels (Figs 2[Up] and 4a[Up]).	7743625:1166
calcium channel --+> voltage-gated Ca2+ channel	Regulation	Accordingly, organic calcium channel antagonists, which block passage of the ion through voltage-gated Ca2+ channels, are potent preglomerular vasodilators (2) and inhibit a broad spectrum of afferent arteriolar vasoconstrictor events, without markedly altering efferent arteriolar resistance or contractile responsiveness (1).	8958219:1051
calcium channel --+> voltage-gated Ca2+ channel	Regulation	Ang II-induced Ca2+ influx in vascular smooth muscle cells is reported to involve voltage-dependent calcium channels which are directly or indirectly activated by Ang II, Ca2+-permeable nonspecific dihydropyridine-insensitive cation channels, receptor-gated Ca2+ channels, Ca2+-activated Ca2+ release, and activation of the Na+/Ca2+ exchanger [10].	15639476:1060
calcium channel --+> voltage-gated Ca2+ channel	Regulation	Exact mechanisms whereby Ang II stimulates Ca2+ influx are unclear but may involve voltage-dependent calcium channels, which are directly or indirectly activated by Ang II, Ca2+-permeable, nonspecific dihydropyridine-insensitive cation channels, receptor-gated Ca2+ channels, Ca2+-activated Ca2+ release channels, and activation of the Na+/Ca2+ exchanger (Arnaudeau et al., 1996; Lu et al., 1996).	11121512:1191
calcium channel --+> voltage-gated Ca2+ channel	Regulation	This conclusion is supported by the quite similar responses generated by acetylcholine, which indirectly depolarizes the chromaffin cells (Douglas et al., 1967[Go]) and activates exocytosis by enhancing Ca2+ entry through Ca2+ channels (Douglas and Poisner, 1961[Go]) or by high K+ concentrations that cause direct cell depolarization (Douglas et al., 1967[Go]) and recruitment of voltage-dependent Ca2+ channels in both control and spontaneously hypertensive rats cells.	17962518:1251
voltage-gated Ca2+ channel ---> calcium channel	Regulation	The beta subunits of voltage-dependent calcium channels bind the pore-forming alpha(1) subunit and play an important role in the regulation of calcium channel function.	17618603:0
voltage-gated Ca2+ channel ---> calcium channel	Regulation	The objective of the present study was to design new protocols to experimentally separate three Ca(2+)-elevating pathways involved in the noradrenaline-induced contractile response, intracellular Ca2+ release, Ca2+ influx through the voltage-dependent (VDCCs) and the receptor-operated (ROCCs) Ca2+ channels with Ca2+ channel blockers in the isolated rat aortic rings.	8902298:1
voltage-gated Ca2+ channel ---> calcium channel	Regulation	In contrast the L-type voltage-dependent calcium-channel has been characterised in great detail and provides the principal planned target of most calcium-channel blockers [2,32].	10728311:1053
voltage-gated Ca2+ channel ---> calcium channel	Regulation	Membrane depolarization caused Ca2+ entry via voltage-dependent Ca2+ channels, which then activated Ca2+ channels and induced Ca2+ release from internal stores.	12205130:1281
voltage-gated Ca2+ channel ---> calcium channel	Regulation	In the cleavage-arrested ascidian muscle blastomere, voltage-dependent calcium channel currents show Ca2+-dependent inactivation as one of the general features of the high-threshold, long-lasting Ca2+ channels.	10066898:1362
voltage-gated Ca2+ channel ---> calcium channel	Regulation	Accordingly, several types of Ca2+ channels have been implicated in the mechanical stress-induced signaling pathways, particularly L-type voltage-dependent Ca2+ channel, and a hypothetical mechanosensitive cation channel that is blocked by gadlinium ions (Gd3+) (6).	15383527:1183
voltage-gated Ca2+ channel ---> calcium channel	Regulation	It has been shown in neurons that Ang II-activated Ca2+ channels are inhibited by the nonspecific voltage-sensitive Ca2+ channel blocker Cd2+.9 As such, cultures were pretreated for 5 minutes with CdCl2 (125 µmol/L) before Ang II stimulation and [Ca2+]i measurements.	15699459:1107

Relation	Type	Sentence	MedLine Reference
voltage-gated Ca2+ channel ---> calcium channel	Regulation	In summary, these data show that the COOH terminus of the human voltage-gated calcium channel (Cav) 1.2b is importantly involved in calcium channel regulation by c-Src kinase and that nitrosylation of Src kinase regulatory sites, as occurs during inflammation, results in marked downregulation.	17942635:1315
voltage-gated Ca2+ channel ---> calcium channel	Regulation	In accord with early findings by Blackmore et al. (34), we found that concentrations of blockers of L-type voltage-gated Ca2+ channels like dihydropyridines and benzothiazepines in excess of what is required to block classical Ca2+ channels had no significant effect on progesterone- and PGE1-induced Ca2+ transients.	9501206:1219
voltage-gated Ca2+ channel ---> calcium channel	Regulation	Since N-methyl-D-aspartate- and KCl-induced depolarization led to an increase in [Ca2+]i, it seemed possible that the increased DNA synthesis was also due to increased Ca2+ influx into the neural progenitor cells through voltage-gated Ca2+ channels as well as N-methyl-D-aspartate-receptor-mediated Ca2+ channels.	17389682:1084
voltage-gated Ca2+ channel ---> calcium channel	Regulation	The novelty of our present findings are that 1) cerebral microvascular endothelial cells in primary culture express receptor-operated and L-type voltage-dependent Ca2+ channels, 2) breakdown products of blood induce elevation of [Ca2+]i in endothelial cells via activation of both receptor- and voltage-operated Ca2+ channels, and 3) increases in ET-1 production from cerebral microvascular endothelial cells caused by structurally dissimilar vasoactive agents found in blood hemolysates are attenuated by Ca2+-free medium, L-type voltage-dependent, and receptor-operated Ca2+ channel blockade.	12388093:1205
GNAI1 --+> mitogen-activated protein kinase	Regulation	RGS13 blocks MAPK activity induced by Galpha(i)- or Galpha(q)-coupled receptors.	11875076:6
GNAI1 --+> mitogen-activated protein kinase	Regulation	ERbeta enhanced NNK-induced cyclic AMP accumulation as well as Galphai-mediated mitogen-activated protein kinase/extracellular signal-regulated kinase (ERK) 1/2 activation.	17638897:5
GNAI1 --+> mitogen-activated protein kinase	Regulation	G alpha i activation leads to the reduction in cAMP (cyclic adenosine monophosphate) levels and to the activation of mitogen activated protein kinases, Erks (extracellular signal-regulated kinases) and p70 S6 kinase.	9917518:11
GNAI1 --+> mitogen-activated protein kinase	Regulation	These data suggest that tyrosine phosphorylation regulates RGS16 function and that EGFR may potentially inhibit Galpha(i)-dependent MAPK activation in a feedback loop by enhancing RGS16 activity through tyrosine phosphorylation.	11602604:10
GNAI1 --+> mitogen-activated protein kinase	Regulation	GAP1(IP4BP)/RASA3 mediates Galphai-induced inhibition of mitogen-activated protein kinase.	18952607:100
GNAI1 --+> mitogen-activated protein kinase	Regulation	Mitogen-activated protein kinase is also activated by Gai (Jo et al., 1997).	12087073:1250
GNAI1 --+> mitogen-activated protein kinase	Regulation	For example, IGF-1-mediated MAP kinase phosphorylation is dependent on Gai/ß? signaling (Luttrell et al, 1995; Dalle et al, 2001).	15241473:1052
GNAI1 --+> mitogen-activated protein kinase	Regulation	Gai and Gao subunits can lead to activation of mitogen-activated protein kinase via a protein kinase C-dependent pathway (12).	11022047:1048

Relation	Type	Sentence	MedLine Reference
GNAI1 --+> mitogen-activated protein kinase	Regulation	Second, Gai activates the MAPK, ERK (24), which has been shown to increase NHE1 activity via p90RSK (35).	17913870:1191
GNAI1 --+> mitogen-activated protein kinase	Regulation	Our data suggest that EG-VEGF receptor might be a G protein-coupled receptor or at least that Gai is required to mediate MAPK activation.	11751915:1255
GNAI1 --+> mitogen-activated protein kinase	Regulation	Consistent with these findings, it has been reported that mitogen-activated protein kinases including p38 can be activated by stimulation of several G proteins including Gai (27, 42, 43).	12093796:1277
GNAI1 --+> mitogen-activated protein kinase	Regulation	However, Ras activation and subsequent engagement of the ERK/mitogen-activated protein kinase can be mediated by the Gai subunit independently of ß? subunits and Ras activation (Hedin et al. 1999).	17158754:1157
GNAI1 --+> mitogen-activated protein kinase	Regulation	To test the hypothesis that resveratrol's activation of Src, MAPK, and eNOS is mediated by Gai, human umbilical vein ECs were pretreated with pertussis toxin (Fig. 4D, E).	18296501:1232
GNAI1 --+> mitogen-activated protein kinase	Regulation	Interestingly, our data contradict these studies because we have found no evidence to support the theory of GnRH-induced activation of these MAPKs by Gai/o, even when Gai is artificially overexpressed in our cell systems.	18801931:1187
GNAI1 --+> mitogen-activated protein kinase	Regulation	Moreover, RGSZ1 suppressed a2A-adrenergic receptor/Gai-mediated MAP kinase activity in PC12 cells and D2R/Gai-mediated serum response element activation in CHO cells.	12379657:1254
GNAI1 --+> mitogen-activated protein kinase	Regulation	Inhibition of Gai/o proteins by pretreatment with pertussis toxin attenuates hypoxia-induced stimulation of DNA synthesis and selectively inhibits mitogen-activated protein kinase activation.	11278727:1296
GNAI1 --+> mitogen-activated protein kinase	Regulation	The inhibition of Gai/o proteins by pretreatment with pertussis toxin attenuates hypoxia-induced stimulation of DNA synthesis and selectively inhibits mitogen-activated protein kinase activation.	12475810:1162
GNAI1 --+> mitogen-activated protein kinase	Regulation	These results clearly indicate that in 3T3L1 adipocytes, IGF-I signaling leading to MAP kinase phosphorylation requires Gai, Gß, and ß-arrestin-1, whereas insulin-induced MAP kinase signaling does not.	11278773:1211
GNAI1 --+> mitogen-activated protein kinase	Regulation	Because both Gas (2, 15, 17) and Gai (15, 18) have been shown to contribute to mitogen-activated protein kinase activation by the conventional agonist isoproterenol, we sought to determine whether this was also the case for drugs with dual efficacy like propranolol or ICI118551.	13679574:1116
GNAI1 --+> mitogen-activated protein kinase	Regulation	Since Gai and Gß? subunits are essential for the activation of mitogen-activated protein kinase by lysophosphatidic acid (30), we examined the expression of Gai and Gß? protein and found that the levels were the same with or without insulin treatment (Fig. 4A).	12167719:1160
GNAI1 --+> mitogen-activated protein kinase	Regulation	In addition, recent studies have shown that RGS4 and Ga-interacting protein block Gai-mediated inhibition of adenylyl cyclase (24), whereas RGS1, RGS2, regulators of G protein signaling3T, and RGS4 attenuate Gai- or Gaq-regulated activation of the ERK group of MAPK (25-27).	9915820:1039

Relation	Type	Sentence	MedLine Reference
GNAI1 --+> mitogen-activated protein kinase	Regulation	In these studies, insulin-induced changes in ß-arrestin1 were associated with increased ßß2-adrenergic receptor/Gas signaling (due to loss of Gas signal desensitization) and with impaired Gai-mediated MAP kinase activation (due to defective ß-arrestin/Src and ß-arrestin/clathrin interaction).	15520010:1075
GNAI1 --+> mitogen-activated protein kinase	Regulation	Signals generated by the activation of G protein-coupled receptors can also be transmitted through Gai and Gas, which can stimulate Mitogen-activated protein kinase by regulating the small GTPase Rap1 (12-14), and by Gaq, which can activate Pyk2 and Src (15, 16), and can stimulate Raf through protein kinase C (17).	10781600:1038
GNAI1 --+> mitogen-activated protein kinase	Regulation	Thus, insulin treatment leads to ß-arrestin-1 Ser412 phosphorylation, ubiquitination, and degradation, all of which impair mitogen-activated protein kinase phosphorylation mediated by Galpha i-coupled receptors, such as the lysophosphatidic acid receptor, ß2-adrenergic receptor (ß2-AR), and the insulin-like growth factor I (IGF-I) receptor (4).	15456867:1035
GNAI1 --+> mitogen-activated protein kinase	Regulation	Role of Shc, G-protein, GPCR, and caveolins The adapter protein Shc, the G-proteins (Gas and Gai), the G-protein-coupled receptor 30 (GPR30), and caveolin-1 have all been involved in estrogen-induced mitogen-activated protein kinase activation by association with estrogen receptora (Migliaccio et al. 1996, Kousteni et al. 2001, Song et al. 2002, Razandi et al. 2003, Revankar et al. 2005).	17259556:1192
GNAI1 --+> mitogen-activated protein kinase	Regulation	The activated calcium-sensing receptor is capable of binding to a number of different G proteins, with preferential activation of Gaq/11 and Gai, which leads to a range of cellular responses, such as stimulation of phospholipase Cß, production of inositol 1,4,5-triphosphate, release of intracellular Ca2+, stimulation of MAPKs, and an inhibition of adenylate cyclase, causing a decrease in cAMP levels (18, 25).	19237714:1132
GNAI1 --+> mitogen-activated protein kinase	Regulation	In addition, H3 receptors activate a number of signal transduction pathways including the Gai/o protein-dependent inhibition of adenylate cyclase activity (Lovenberg et al., 1999[Go]; Drutel et al., 2001[Go]) and the isoform dependent activation of mitogen-activated protein kinase and arachidonic acid release by the rat H3 receptor (Drutel et al., 2001[Go]), perhaps influencing the clinical potential of H3 receptor antagonists.	15608078:1064
GNAI1 --+> mitogen-activated protein kinase	Regulation	Some reports have linked oxidants with a direct activation of the nonreceptor tyrosine kinase Src in mouse fibroblasts and erythrocytes (Abe et al., 1997[Go]; Mallozzi et al., 1999[Go]), whereas others have shown that small G proteins like Gai and Gao contribute to Reactive oxygen species-induced mitogen-activated protein kinase activation in cardiomyocytes (Nishida et al., 2000[Go]).	15574683:1066
GNAI1 --+> mitogen-activated protein kinase	Regulation	The Gß? subunit, by virtue of its ability to interact with certain pleckstrin homology domains, might influence the activity of either mSOS or Ras-GEF, both of which have pleckstrin homology domains.37 Gß? generated by stimulation of muscarinic receptors activates ERK2 via a Ras-dependent pathway, possibly involving Shc.28 Phosphatidyl inositol 3 kinase-?, which is activated by Gß?,38 has been implicated in Gai-mediated mitogen-activated protein kinase activation.39 Phosphatidyl inositol 3 kinase-? has also been shown to mediate shear stress-dependent activation of JNK.40 However, the specific mechanism by which Gß? activates Ras in fluid flow-stimulated endothelial cells remains to be clarified.	12714438:1198
GNAI2 --+> mitogen-activated protein kinase	Regulation	However, under identical experimental conditions, activated forms of Gai2, Gaq, Gs, or G12 were not able to induce MAPK activation (35).	9442012:1077
GNAI2 --+> mitogen-activated protein kinase	Regulation	It might be that activation of extracellular signal-regulated kinases through a Gi-dependent pathway was dependent on the abundance of Gai2 protein in C2C12 and NFB4 cells.	9415407:1160
GNAI1 ---> phospholipase A2	Regulation	G alpha i-3 regulates epithelial Na+ channels by activation of phospholipase A2 and lipoxygenase pathways.	2174882:100

Relation	Type	Sentence	MedLine Reference
GNAI1 ---> phospholipase A2	Regulation	A. (1990) G alpha i-3 regulates epithelial Na+ channels by activation of phospholipase A2 and lipoxygenase pathways.	14563210:1209
GNAI1 ---> phospholipase A2	Regulation	B2 receptor activation liberates prostaglandins and other arachidonic acid metabolites38,39 and involves first Gai protein leading to downstream activation of phospholipase A2.	16246972:1195
BASP1 --+> caspase	Regulation	Taken together, these data suggest the involvement of caspases and mitochondria in the cell death process activated by BASP1 overexpression. [Figure 6.] View larger version: In this window.	20110383:1145
calcineurin ---> voltage-gated Ca2+ channel	Regulation	Together, the results provide the first evidence that calcineurin activity, but not increased expression, plays a selective and necessary role in the aging-related increase in available L-type voltage sensitive Ca(2+) channels, possibly by direct activation.	18471936:9
calcineurin ---> voltage-gated Ca2+ channel	Regulation	In neurons, we have found that calcineurin stabilizes or enhances L-voltage-sensitive Ca2+ channel activity in an aging-dependent manner, both in culture and in vivo (87, 88).	18541537:1362
calcineurin ---> voltage-gated Ca2+ channel	Regulation	Finally, It is noteworthy that calcineurin is an important modulator of other vascular smooth muscle channels, including, but not limited to, calcium-activated chloride-channels and voltage-gated Ca2+ channels.	19630834:1146
calcineurin ---> voltage-gated Ca2+ channel	Regulation	For example, calcineurin enhances the inactivation of voltage-gated Ca2+ channels in molluscan neurons (Chad and Eckert 1986), and modulates glutamatergic synaptic transmission via a presynaptic mechanism of action in rat cortex (Nichols et al. 1994; Victor et al. 1995).	9307145:1038
calcineurin ---> voltage-gated Ca2+ channel	Regulation	The down-regulation of voltage-dependent Ca2+ channels is prevented by treatment with a dihydropyridine voltage-dependent Ca2+ channels blocker (29) or by pretreatment with the calcineurin inhibitor cyclosporin A (42), suggesting that the changes in voltage-dependent Ca2+ channels expression are Ca2+-dependent and that the Ca2+-dependent modulation of voltage-dependent Ca2+ channels expression may be regulated by NFAT.	11278965:1242
voltage-gated Ca2+ channel --+> calcineurin	Regulation	We confirmed that a rise in intracellular cyclic AMP concentration stimulated cells to increase their neurite numbers, and that this increase of neurites was suppressed by activation of calcineurin induced by a Ca2+ influx through voltage-dependent Ca2+ channels.	12957367:2
voltage-gated Ca2+ channel --+> calcineurin	Regulation	The Ca(2+)/calmodulin-dependent protein phosphatase, calcineurin, modulates a number of key Ca(2+) signaling pathways in neurons, and has been implicated in Ca(2+)-dependent negative feedback inactivation of N-methyl-D-aspartate receptors and voltage-sensitive Ca(2+) channels.	11958864:0
voltage-gated Ca2+ channel --+> calcineurin	Regulation	calcineurin/NFAT activation in astrocytes selectively depends on L-voltage-sensitive Ca2+ channels.	18541537:1239
voltage-gated Ca2+ channel --+> calcineurin	Regulation	In excitable cells, an increase in intracellular calcium via voltage-gated calcium channels activates calcineurin resulting in the dephosphorylation and nuclear translocation of transducer of regulated CREB activity.	20172974:1074
voltage-gated Ca2+ channel --+> calcineurin	Regulation	Importantly, it has been shown that L-type voltage-sensitive Ca2+ channel-dependent activation of calcineurin phophatase dephosphorylates and in turn non-sumoylates MEF2A and promotes synapse disassembly.	16793900:1109

Relation	Type	Sentence	MedLine Reference
voltage-gated Ca2+ channel --+> calcineurin	Regulation	These results indicate that free calcium ion influx through voltage-gated calcium channels activates PP2B, which in turn is involved in the dephosphorylation of Glycogen synthase kinase 3ß following KCl-induced depolarization.	15799972:1229
voltage-gated Ca2+ channel --+> calcineurin	Regulation	Since blocking ryanodine receptor would be expected to lead to membrane depolarization due to diminished BK channel activity (45), it is conceivable that the activity of Ca2+/calmodulin-dependent calcineurin might be enhanced by virtue of an increased Ca2+ flux through voltage-dependent Ca2+ channels.	12145283:1208
CNRIP1 --+> voltage-gated Ca2+ channel	Regulation	Furthermore, in superior cervical ganglion neurons coinjected with CB1 and CRIP1a or CRIP1b cDNA, CRIP1a, but not CRIP1b, suppresses CB1-mediated tonic inhibition of voltage-gated Ca2+ channels.	17895407:4
CNRIP1 --+> voltage-gated Ca2+ channel	Regulation	CRIP1a can interact with the distal C terminus of CB1R (Niehaus et al., 2007) and may attenuate CB1R-mediated inhibition of voltage-gated Ca2+ channels.	20962221:1193
GSTA1 ---| caspase	Regulation	Surprisingly, GSTs M1, P1, as well as GST A1, inhibited ASK1-induced DEVD-AMC caspase activity.	12370186:1235
GSTA1 ---| caspase	Regulation	Surprisingly, GSTs M1, P1, as well as GST A1, inhibited ASK1-induced DEVD-AMC caspase activity.	10781611:1235
RALA --+> mitogen-activated protein kinase	Regulation	We have previously reported that forced expression of RalA prolongs growth hormone-stimulated p44/42 MAP kinase activity (29).	12734187:1401
RALA --+> mitogen-activated protein kinase	Regulation	Thus, RalA is required for full activation of p44/42 MAP kinase activity by hGH in NIH-3T3 cells. [Figure 5] View larger version: In this window.	12215457:1278
RALA --+> mitogen-activated protein kinase	Regulation	Thus, RalA is required for full activation of p44/42 MAP kinase activity by hGH in NIH-3T3 cells. [Figure 5] View larger version: In this window.	12218045:1278
GNAI2 --+> phospholipase A2	Regulation	Activation of cytoplasmic phospholipase A2 is inhibited by pertussis toxin and G alpha i2 mutants.	7601096:2
GNAI2 --+> phospholipase A2	Regulation	This hypothesis of dual regulation of cytosolic phospholipase A2 by mitogen-activated protein kinase and protein kinase C[alpha] for maximal activation is consistent with other observations, such as the demonstration that cytosolic phospholipase A2 possesses distinct phosphorylation sites for mitogen-activated protein kinase and protein kinase C (6), and that in Chinese hamster ovary cells, transfection of a mutant G[alpha]i2 inhibits cytosolic phospholipase A2-mediated arachidonic acid release by P2-purinergic receptors without affecting mitogen-activated protein kinase activation and cytosolic phospholipase A2 phosphorylation by mitogen-activated protein kinase (40).	9045886:1261
PSAP --+> mitogen-activated protein kinase	Regulation	We report that prosaposin treatment induced extracellular signal-regulated kinases and sphingosine kinase activity, increased DNA synthesis, and prevented cell apoptosis.	11156962:0
PSAP --+> mitogen-activated protein kinase	Regulation	This effect was inhibited by mitogen-activated protein ERK kinase (MEK) and sphingosine kinase inhibitors, indicating that prosaposin prevents cell apoptosis by activation of extracellular signal-regulated kinases and sphingosine kinase.	15242760:3
PSAP --+> mitogen-activated protein kinase	Regulation	It was also shown that prosaposin stimulates the phosphorylation of mitogen-activated protein kinase [26].	9895286:1057

<table>
<tr><th>Relation</th><th>Type</th><th>Sentence</th><th>MedLine Reference</th></tr>
<tr><td>PSAP --+> mitogen-activated protein kinase</td><td>Regulation</td><td>In neuro-glial derived cells, neurotrophic activity, cell-death protection and the activation of mitogen-activated protein kinase by prosaptides (i.e., TX14A), saposin C, or prosaposin are mediated by their binding to a pertussis toxin-sensitive G-protein coupled receptor [6,7,12,13].</td><td>15548330:1151</td></tr>
<tr><td>PSAP --+> mitogen-activated protein kinase</td><td>Regulation</td><td>O'Brien et al. (1994) have postulated the presence of a high-affinity prosaposin receptor on the surface of a neuroblastoma cell line, and prosaposin induces mitogen-activated protein kinase phosphorylation in PC12 cells (Campana et al., 1996) and in Schwann cells and oligodendrocytes (Hiraiwa et al., 1997).</td><td>10078882:1236</td></tr>
<tr><td>PSAP --+> mitogen-activated protein kinase</td><td>Regulation</td><td>Our present demonstration that prosaposin, saposin C, and prosaptides stimulate mitogen-activated protein kinase phosphorylation and increase the sulfatide content in both Schwann cells and in oligodendrocytes suggests that hypomyelination in the prosaposin-deficient human and in the transgenic prosaposin knockout mouse is due to a deficiency of the trophic action of prosaposin on myelination.</td><td>9114068:1146</td></tr>
<tr><td>CNRIP1 ---> voltage-gated ion channel</td><td>Regulation</td><td>CRIP1a may function to keep agonist-independent regulation of voltage-gated ion channels by CB1 receptors in check in neurons in which CRIP1a and CB1 receptors are colocalized.</td><td>17895407:1278</td></tr>
<tr><td>ABAT ---| neuronal plasticity</td><td>Regulation</td><td>Treatment of epilepsy: the GABA-transaminase inhibitor, vigabatrin, induces neuronal plasticity in the mouse retina.</td><td>18412635:100</td></tr>
<tr><td>AHSG ---| ROS generation</td><td>Regulation</td><td>Of these, AHSG was the most active inhibitor of hydroxyapatite-induced neutrophil superoxide release, and this glycoprotein partially (60%) restored inhibitory activity to hydroxyapatite-adsorbed serum.</td><td>2844196:5</td></tr>
<tr><td>BASP1 --+> cell differentiation</td><td>Regulation</td><td>WT1 and BASP1 co-operate to induce the differentiation of K562 cells to a neuronal-like morphology that exhibits extensive arborization, and the expression of several genes involved in neurite outgrowth and synapse formation.</td><td>21269271:5</td></tr>
<tr><td>SPTAN1 ---> neurite outgrowth</td><td>Regulation</td><td>Our results indicate that the phosphorylation-dependent interaction between 14-3-3beta and alphaII spectrin acts as a switch between positive and negative regulation of neurite outgrowth stimulated by NCAM, representing a novel and acute mechanism preventing uncontrolled elongation of neuronal processes.</td><td>20598904:6</td></tr>
<tr><td>PSAP ---> cell adhesion</td><td>Regulation</td><td>Prosaposin down-modulation decreases metastatic prostate cancer cell adhesion, migration, and invasion.</td><td>20132547:100</td></tr>
<tr><td>NFASC ---> neurite outgrowth</td><td>Regulation</td><td>Here, we show a new link between FGFR1 and the cell adhesion molecule neurofascin, which is important for neurite outgrowth.</td><td>19666467:1</td></tr>
<tr><td>NFASC ---> neurite outgrowth</td><td>Regulation</td><td>Neural cell adhesion molecule neurofascin regulates the induction of neurite outgrowth, the establishment of synaptic connectivity and myelination.</td><td>16314110:0</td></tr>
<tr><td>calcium channel --+> glycolysis</td><td>Regulation</td><td>Calcium-channel blocking agents and drugs promoting anaerobic glycolysis are designed to stop the intracellular processes causing ischemia.</td><td>9949976:4</td></tr>
<tr><td>calcium channel --+> glycolysis</td><td>Regulation</td><td>The stimulation of glycolysis-Krebs by [Ca2+]i was inhibited by a mitochondrial calcium channel blocker (Ruthenium red) and persisted over a range of ATP/ADP ratios.</td><td>1721544:6</td></tr>
<tr><td>AHSG ---> cell differentiation</td><td>Regulation</td><td>The AHSG/fetuin gene may have a role in differentiation since it is expressed in mouse limb buds and brain only at certain stages during development.</td><td>1373325:5</td></tr>
<tr><td>AHSG ---| mitogen-activated protein kinase</td><td>Regulation</td><td>This inhibition results in reduced guanine nucleotide exchange in p21ras. alpha 2-HS glycoprotein also inhibits the stimulation of Raf phosphorylation, in response to insulin, leading to inhibition of MEK activity.</td><td>9115849:6</td></tr>
</table>

Relation	Type	Sentence	MedLine Reference
GNAI1 ---| oxidative stress	Regulation	Mutational activation of a Galphai causes uncontrolled proliferation of aerial hyphae and increased sensitivity to heat and oxidative stress in Neurospora crassa.	9872952:100
calcium channel ---> voltage-gated ion channel	Regulation	Adrenergic modulation of calcium channels profoundly influences cardiac function, and has served as a prime example of neurohormonal regulation of voltage-gated ion channels.	6320002:0
PFKP ---> glycolysis	Regulation	With its distinct allosteric properties PFKP is regarded to be the key enzyme for the regulation of glycolysis in this organ.	15716112:2
PLP1 ---> neurite outgrowth	Regulation	One interpretation of these findings is that expression of the mutant DM20 alters signaling between oligodendrocytes and neurons, producing abnormal neurite outgrowth.	10581470:6
GNAI2 ---> cell growth	Regulation	We hypothesized that Galphai2 is expressed in the lung during ontogeny in a growth-dependent manner, and that Galphai2 regulates cell growth.	9870915:3
SPTAN1 ---> cell adhesion	Regulation	AlphaII-spectrin is critical for cell adhesion and cell cycle.	18978357:100
calcineurin ---> voltage-gated ion channel	Regulation	Several other ligand- and voltage-gated ion channels are negatively regulated by calcineurin.	9149541:2
